# Maast: genotyping thousands of microbial strains efficiently

**DOI:** 10.1186/s13059-023-03030-8

**Published:** 2023-08-10

**Authors:** Zhou Jason Shi, Stephen Nayfach, Katherine S. Pollard

**Affiliations:** 1 Chan Zuckerberg Biohub, San Francisco, CA USA; 2 Gladstone Institutes of Data Science and Biotechnology, San Francisco, CA USA; 3 Joint Genome Institute, Department of Energy, Walnut Creek, CA USA; 4 Environmental Genomics and Systems Biology Division, Lawrence Berkeley National Laboratory, Berkeley, CA USA; 5 Department of Epidemiology and Biostatistics, University of California San Francisco, San Francisco, CA USA

## Abstract

Existing single nucleotide polymorphism (SNP) genotyping algorithms do not scale for species with thousands of sequenced strains, nor do they account for conspecific redundancy. Here we present a bioinformatics tool, Maast, which empowers population genetic meta-analysis of microbes at an unrivaled scale. Maast implements a novel algorithm to heuristically identify a minimal set of diverse conspecific genomes, then constructs a reliable SNP panel for each species, and enables rapid and accurate genotyping using a hybrid of whole-genome alignment and k-mer exact matching. We demonstrate Maast’s utility by genotyping thousands of *Helicobacter pylori* strains and tracking SARS-CoV-2 diversification.

**Supplementary Information:**

The online version contains supplementary material available at 10.1186/s13059-023-03030-8.

## Background

Many bacterial and viral species now have thousands of sequenced genomes in public databases, and these numbers are rapidly increasing, fueled by technologies such as metagenome assembly, high-throughput culturing, and single-cell genome sequencing. For many species, genome collections harbor immense genetic variation [1, 2]. Single nucleotide polymorphisms (SNPs) are genomic positions that vary between genomes of the same species with a minimum minor allele frequency (e.g., 1%). Vertically inherited SNPs in conserved genes are commonly used to reconstruct phylogenies and study biogeography [3, 4], while SNPs in pathogenicity loci and antibiotic resistance genes are leveraged for surveillance of medically important strains [5–7]. Compared to multilocus sequence typing methods, whole-genome SNP genotyping generally enables greater phylogenetic resolution [8]. Comparing genomes based on a pre-defined set of SNPs is also more computationally scalable than using whole-genome nucleotide identity [9] and thus especially well-suited for the analysis of large genome collections.

SNPs are often identified from whole genome sequences. For example, Parsnp [10] constructs multiple sequence alignments of high-quality genome assemblies and identifies variable positions directly from the alignments. An alternative method is to call SNPs from sequencing reads without genome assembly. For example, Snippy (https://github.com/tseemann/snippy) identifies SNPs from the alignment of short reads to a reference genome and kSNP [11] identifies SNPs using informative k-mers found on short reads, contigs, or genome assemblies. Assembly-free genotyping and k-mer matching are usually faster but less accurate than genotyping SNPs from whole-genome alignments [12]. With all of these strategies, SNPs of interest can be extracted using thresholds on the minor allele frequency (MAF), site prevalence, or protein-coding change across a set of strains.

Despite the variety of SNP genotyping methods, a rapid increase in the number of sequenced microbial genomes presents a computational challenge for existing tools. Sequence alignment is the major obstacle to analyzing so many genomes, though kSNP also remain largely untested with thousands of strains. A second challenge is the fact that many species have a high level of genome redundancy [13], especially when a biased sample of clonally related genomes has been sequenced, which is common for clinically important pathogens that are under intensive surveillance (e.g., PulseNet [14] and NCBI Pathogen Detection). This redundancy masks the diversity of unevenly sampled species, and it means that strains from poorly sampled lineages contribute little to the discovery of SNPs, especially when a relatively high MAF threshold is used. Redundancy also alters the meaning of MAF and site prevalence; frequency in the sampled genomes is not a good estimate of frequency in the population. Together, these two challenges limit the utility of SNP genotyping methods for many microbial species.

To address this gap, we present Maast (Microbial agile accurate SNP Typer) for accurate genotyping of orders of magnitude more microbial strains than other state-of-the-art methods. Our key innovation is an algorithm to pick a minimal set of maximally diverse genomes. Only these genomes are used for SNP discovery which reduces genomic redundancy and computational cost. We also implement a hybrid method combining whole-genome alignment and optimized k-mer exact match for genotyping SNPs in either assembled genomes or unassembled whole-genome sequencing (WGS) libraries. Maast performs SNP genotyping faster and more accurately than existing methods but sometimes misses rare variants present in genomes not selected for SNP discovery. We apply Maast to a large collection of previously sequenced *Helicobacter pylori* (*H. pylori*) strains and summarize the biogeographic patterns of this species across the globe. We also show that Maast can efficiently track the evolution of SARS-CoV-2 during the COVID-19 outbreak. Maast is available as open-source software with both source code and documentation freely accessible on GitHub (https://github.com/zjshi/Maast).

## Results

### The Maast SNP genotyping pipeline

**Fig. 1:**
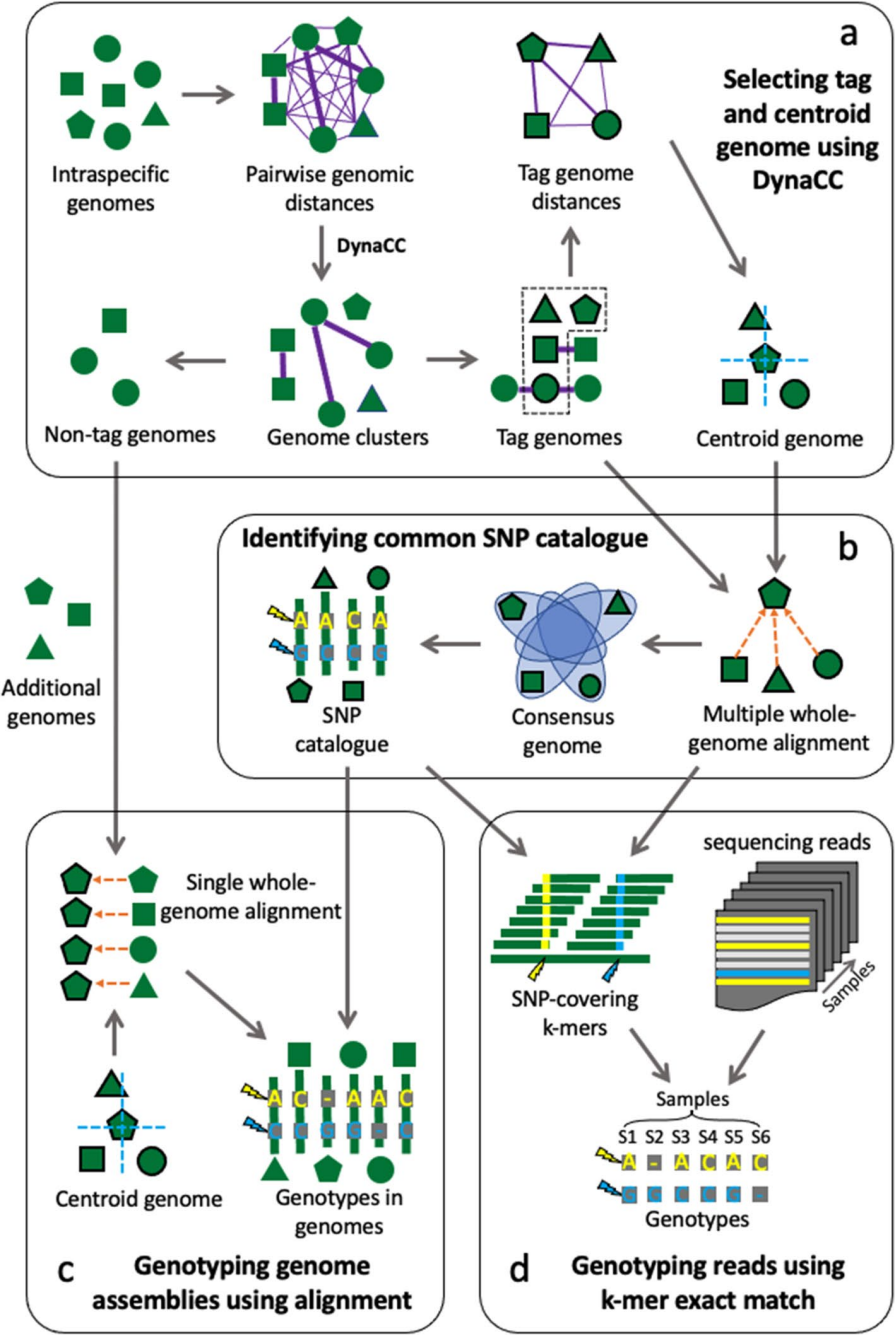
Schematic design of Maast. Maast consists of four major components. **a** Tag genomes and centroid genomes are selected based on Mash distances. A novel algorithm, called DynaCC, is used to automatically choose the genome clustering threshold based on each species’ level of genomic redundancy (see the “Methods” section). **b** A panel of common SNPs is constructed for each species using multiple whole-genome alignment. **c** SNPs in non-tag genomes or other input genomes are genotyped using single whole-genome alignment. **d** SNPs in short reads are genotyped using k-mer exact matching

Maast is an open-source bioinformatics pipeline written in Python and C +  + that fully automates SNP calling and genotyping for microbial species. Maast has two components: (1) constructing a reference panel of SNPs for a microbial species using a reduced set of non-redundant genomes (Fig. 1a and b, Additional file 1: Figure S1), and (2) ultra-fast, in silico genotyping of reference SNPs from large scale genome collections. Genotyping can be performed using draft genome assemblies or unassembled short or long reads (Fig. 1c, d and Additional file 1: Figure S2) and can be applied to any microbial species, such as bacteria, archaea, viruses, or microbial eukaryotes.

In the first step of the pipeline, Maast rapidly builds a reference SNP panel from a diverse, non-redundant subset of input genome assemblies using MUMmer. The genome subset is identified using pairwise genetic distances and a dynamic graph algorithm that we developed and called DynaCC (Dynamic Connected Component search; Additional file 1: Figure S3). DynaCC aims to identify a minimal set of genomes that captures the maximum number of common SNPs with MAF above a user-specified value. It does this heuristically in order to accelerate the computation, and hence the resulting “tag” genomes are not provably a minimal set. From the tag genomes, Maast selects one centroid tag genome to be used as a species reference. Maast then aligns all other tag genomes to the reference using MUMmer, constructs a multiple sequence alignment (Additional file 1: Figure S2), and uses a standard SNP calling workflow (“Methods”) to generate the SNP panel at the user-specified MAF and prevalence (% of genomes containing either SNP allele).

**Fig. 2:**
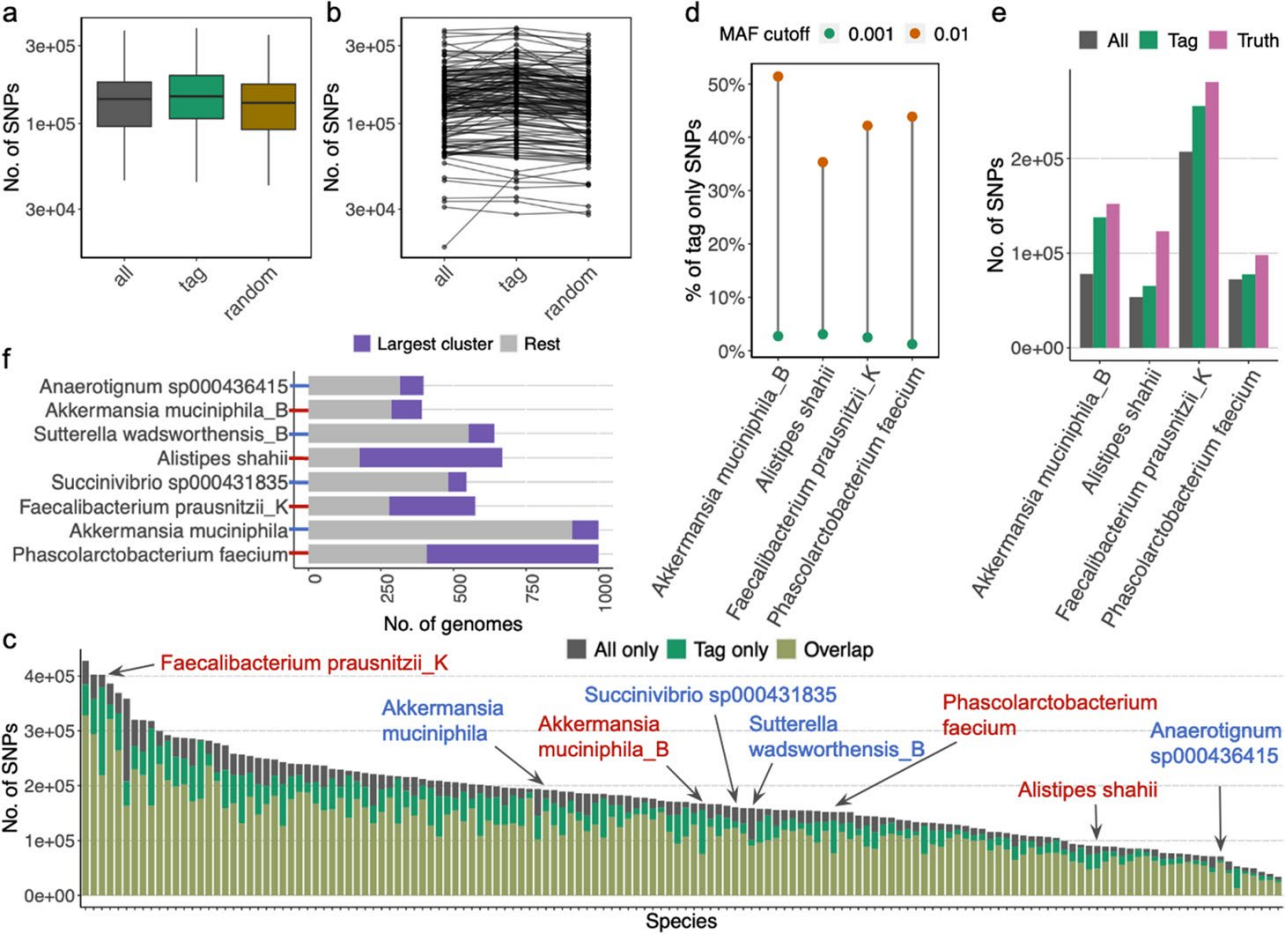
SNP genotyping of 146 human gut bacterial species using tag genomes. **a** and **b** SNP discovery comparison of Maast with all genomes (gray), only tag genomes (green), and a random set of genomes equal in number to the tag genomes (brown) shows that more genomes do not lead to the discovery of more SNPs. Each box in **a** summarizes the number of SNPs across 146 species. Each point in **b** represents a species, with black lines connecting the data for the same species. For computational efficiency, only the 1000 highest quality genomes were included for species with > 1000 genomes. **c** Comparison of SNPs discovered by Maast with all genomes versus only tag genomes. Each bar represents a species, with the height of a bar showing the number of SNPs discovered exclusively with all genomes (gray), exclusively with tag genomes (green), or by both approaches (beige). Arrows point to eight example species (from left to right): *Faecalibacterium prausnitzii_K* (species id: 101300), *Akkermansia muciniphila* (102454), *Akkermansia muciniphila_B* (102453), *Succinivibrio sp000431835* (100412), *Sutterella wadsworthensis_B* (101361), *Phascolarctobacterium faecium* (103439), *Alistipes shahii* (100003), and *Anaerotignum sp000436415* (100177). Species label color indicates whether this species has a high (red) or low (blue) level of tag-only SNPs, which is estimated as a fraction of all SNPs that are discovered with tag genomes and not with all genomes. **d** SNP sites missing from a small percentage of genome assemblies as they fall below the user-specified prevalence threshold due to being absent in a group of redundant genomes. Connected dots represent example species in **c** with a high proportion of tag-only SNPs. The proportion of tag-only SNPs drops if the MAF cutoff for calling SNPs with all genomes is lowered from 0.01 (orange) to 0.001 (green). Most of the SNPs only discovered with tag genomes (tag-only SNPs) are due to MAFs below the 1% threshold in all genomes. **e** For each of the four species where tag genomes and all genomes called different numbers of SNPs (*Faecalibacterium prausnitzii_K*, *Phascolarctobacterium faecium*, *Alistipes shahii*, and *Akkermansia muciniphila_B*), we further investigated the source of this discrepancy by defining a set of true population SNPs. To do so, we built a phylogenetic tree and used it to sample genomes so that they covered the species diversity but came from different subpopulations (essentially removing bias from over-sampling of subpopulations with redundant genomes). SNPs called using this set of genomes were defined as the true SNPs for the population. We ran Maast on each species using tag genomes or using all genomes to call SNPs with a 1% MAF threshold. For each species, tag genomes capture a higher number of the true SNPs than do all genomes, suggesting that redundant genomes bias MAF estimation and reduce SNP calling sensitivity. **f** The single largest genome cluster is larger for species with many tag-only SNPs compared to those with fewer. The largest genome clusters were compared between species from **c** with high (red axis tick) and low (blue axis tick) levels of tag-only SNPs. For three of four species with more SNPs discovered in the tag-only analysis, the largest cluster contains more than half of all genomes, implying a high level of genome redundancy that biases MAF estimation and leads to an undercount of SNPs. Height of bars shows the total number of genomes in a species and the proportion colored in purple indicates the size of the single largest genome cluster of that species. Every two adjacent species have a similar total number of genomes

We assessed whether the Maast strategy of subsampling reduced our power to detect SNPs on a benchmark dataset of 146 common bacterial species from the human gut, each with at least 200 high-quality genomes (Additional file 2: Table S1). These species have different levels of intraspecific diversity (Additional file 1: Figure S4), genomic redundancy (Additional file 1: Figure S5a), and SNP density (Additional file 1: Figure S5b). Overall, we found that the number of SNPs identified with Maast tag genomes (> 1% MAF) was equal to or greater than the number of SNPs identified using the full set of genomes (Fig. 2a, b), with high overlap between the approaches (median = 87.9%, Fig. 2c). Our results demonstrate that more genomes do not necessarily lead to the discovery of more common SNPs (> 1% MAF). The presence of many highly related genomes also leads to biased estimation of SNP frequencies in the overall population and may reduce SNPs discovered at a given MAF threshold (Fig. 2d, e, f, S6). These analyses highlight that the Maast strategy of subsampling captures the most common genetic variation and successfully represents genomes from poorly sampled lineages (Additional file 1: Figure S7, S8). Users also have the option to run Maast with all genomes rather than tag genomes, which may enable more of the SNPs present in pairwise genome comparisons to be included in the SNP panel, especially for rare SNPs present in less divergent pairs of genomes (Fig. 2d, Additional file 1: Figure S8), but at the cost of greater computational requirements. Maast, run using either tag genomes or all genomes, misses many variable sites between pairs of genomes (Additional file 1: Figure S7, S8), because these are largely rare variants whereas Maast was designed to capture common SNPs above a MAF threshold.

In the second step of the pipeline, Maast performs reference based in silico genotyping of common, bi-allelic SNPs in genomes, which may be non-tag genomes from the initial reference collection or totally new genomes. Like other methods that genotype using a single reference genome, Maast cannot genotype sites that are absent from the reference genome, and in its current implementation, Maast also does not aim to genotype rare SNPs or SNPs with more than two alleles. The workflow can be executed using genome assemblies or unassembled WGS reads. For genome assemblies, Maast aligns each query genome to the centroid genome for that species using MUMmer and directly genotypes alleles for each of the reference SNPs. For WGS reads, Maast performs exact matching between k-mers extracted from the sequencing reads and a database of k-mers covering each SNP in the reference panel that has been curated to only include k-mers that do not occur anywhere else in the entire reference genome collection of tag and non-tag genomes (Additional file 1: Figure S2). The algorithm leverages the fact that most query k-mers have no chance to match sck-mers in the database. It screens subsequences of colex-ordered k-mers up front, which is faster and more RAM-efficient than using a hash table (“Methods”). This strategy is an extension of our previous method GT-Pro [15] for genotyping SNPs in metagenomic sequencing libraries, specifically optimized for WGS of microbial isolates.

### Maast is orders of magnitude faster than existing tools

We evaluated the computational performance of Maast with kSNP3 and Parsnp, two other commonly used methods for microbial SNP calling. All methods were run for a single bacterial species, *Agathobacter rectalis* (Additional file 3: Table S2)*,* using 500, 1000, or 5000 randomly sampled genome sequences (Methods). Only SNP calling was benchmarked, and not downstream steps such as tree building. Surprisingly, both kSNP3 and Parsnp failed to run through all the *A. rectalis* genomes. kSNP3 was manually terminated after running for a maximum allowed time (48 h) and consuming more than 1 TB disk space, while Parsnp failed to run even for 1,000 genomes, apparently due to high intraspecific genetic diversity. To check that these results were not specific to *A. rectalis*, we ran both tools on two other human gut species (Additional file 3: Table S2): *Alistipes putredinis* (*n* = 3646) and *Bacteroides_B dorei* (*n* = 3170) and obtained the same result. We conclude that, to our knowledge, Maast is the only tool able to call SNPs in species with thousands of diverse genomes.

**Fig. 3:**
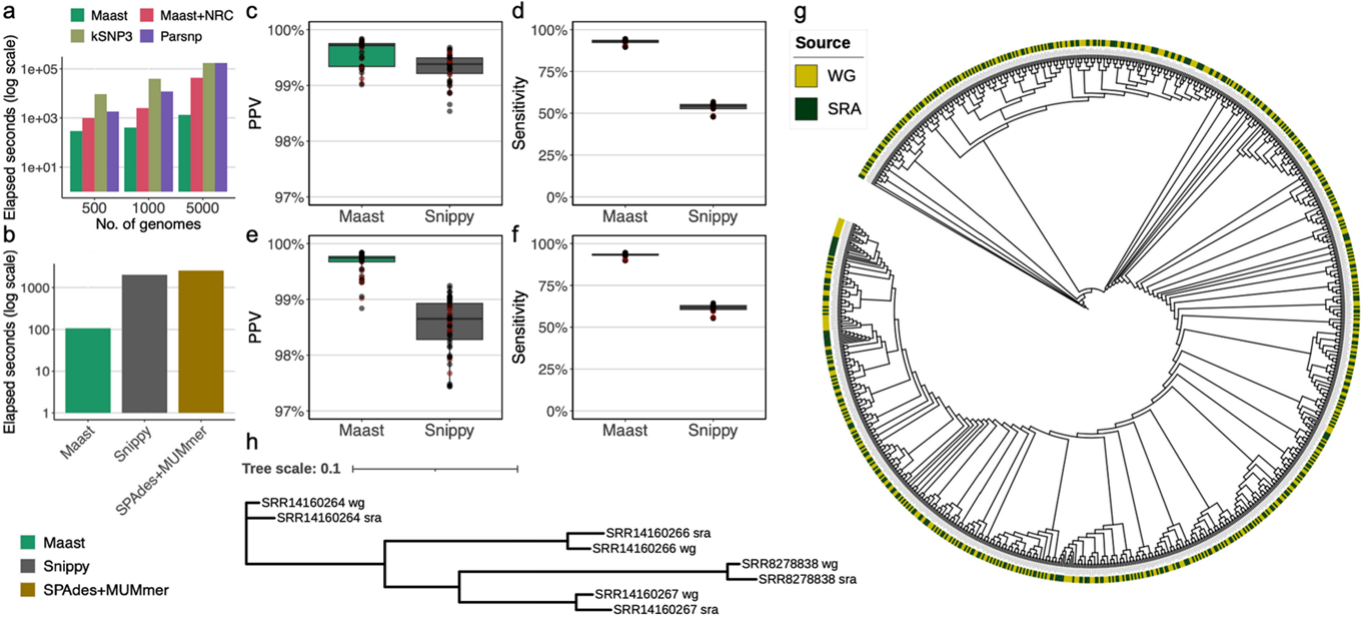
Evaluation of Maast computational performance and accuracy. **a** Comparison of genome genotyping speed between Maast, Maast without redundancy collapsing (Maast + NRC), kSNP3, and Parsnp. All methods were run on 500, 1000, and 5000 simulated *A. rectalis* genomes. **b** Comparison of short-reads genotyping speed between Maast, Snippy, and SPAdes. All three methods were run on 63 strains of *B. uniformis*, whose whole genome sequencing reads (~ 150 million) were downloaded from the Culturable Genome Reference (CGR) study. The *y*-axis of both **a** and **b** indicates elapsed seconds of running in log scale. Fewer elapsed seconds indicate better performance (faster processing speed). **c**–**f** Comparison of Maast and Snippy genotyping accuracy at non-reference alleles of SNPs in the Maast SNP panel, based on short reads **c**, **d** simulated from isolate genomes with sequencing error (15 x coverage) and **e**,** f** downloaded from isolate whole-genome sequencing projects. Both Maast and Snippy were run with default settings. **c**, **e** Positive predictive value (PPV; 1- false discovery rate) comparison, where false discoveries are genotype calls that do not match the genome. **d**, **e** Sensitivity from the simulations in **c** and downloaded reads in **e**. Sensitivity is the probability of detecting genotypes present in the genome. Color of points in **c**–**f** indicates whether the data comes from tag genomes (black) or not (red). Samples colored in red are regarded as novel to Maast databases. **g**, **h** Maast genotype concordance between **g** genome and short reads or **h** genome and long reads. In **g**, strain population structure of *H. pylori* was reconstructed using SNPs from 473 strains. Each strain has a whole genome sequence (WG) and a short read sample (SRA) as indicated in the stacked color rings. In **h**, the population structure of *H. pylori* was reconstructed from 4 strains with whole genome sequence (WG) and long reads (SRA). * kSNP3 and Parsnp runs > 48 h on 5000 genomes and were manually terminated, and we plot a runtime of 48 h with the note that no output was produced

To evaluate computational performance when genomic divergence is more limited and in the absence of structural variation, we simulated 5000 whole genome sequences of *A. rectalis* by randomly introducing SNPs across a representative genome (“Methods”). While Parsnp ran to completion, kSNP3 again exceeded our 48-h maximum time window. Overall, Maast was 6.3 to > 127-fold faster than Parsnp and 31 to > 127 fold faster than kSNP3 (Fig. 3a). In addition, Maast required only 7.2 GB of RAM to process the 5,000 genomes, which was substantially less than what Parsnp used to process 100 genomes and similar to what kSNP3 used to process 1000 genomes (Additional file 4: Table S3). Maast also only used a moderate amount (~ 1.2 GB) of disk space. We attribute Maast’s high speed and RAM efficiency mainly to the strategy of subsampling genomes for SNP discovery. Other factors included compact data structures and parallel processing. These results demonstrate that Maast can easily run on a personal computer.

We also evaluated the computational performance of Maast when calling SNPs in WGS reads. As a benchmark, we selected 63 whole genome sequencing samples of *Bacteroides uniformis* from the Culturable Genome Reference (CGR) study (“Methods”, Additional file 5: Table S4, mean = 2.38 million reads per sample). Performance was compared with Snippy as well as an assembly-based strategy using a combination of SPAdes and MUMmer. Maast was ~ 19–24 fold faster than these methods (Fig. 3b, d) taking < 2 s to process each WGS sample and requiring 11.8 GB RAM. Altogether, we conclude that Maast greatly accelerates SNP genotyping in both assemblies and short reads and is friendly to personal computing devices.

### Maast SNP genotypes are highly accurate

To validate the accuracy of Maast SNP genotypes, we first compared Maast to Parsnp and kSNP on 1000 *A. rectalis* genomes with simulated SNPs (“Methods”). Erroneous alignments can in theory produce both incorrect genotypes (false positives) and missing genotypes (false negatives). False positives were only observed for kSNP (Additional file 6: Table S5) while false negatives were very low for Maast (*n* = 1) and Parsnp (*n* = 4) but much higher for kSNP (*n* = 5073). These results suggest that Maast is at least as accurate as currently available methods.

To evaluate the genotyping accuracy of Maast on short reads, we compared Maast to Snippy by running both tools with their default settings on Illumina short reads simulated from 45 isolate genomes of a single species (*B. uniformis*, Additional file 5: Table S4, 15 x coverage per genome). For each method, predicted genotypes were compared to a set of ground truth genotypes determined from whole-genome alignment of the 45 isolate genomes to the Maast reference genome. Across simulations from the 45 isolates, the median SNP positive predictive value (PPV) was very high for both methods (Maast median = 99.72%, Snippy median = 99.38%; Fig. 3c and Additional file 1: Figure S9a). Meanwhile, the sensitivity of Maast was consistently higher (median = 93.2%) compared to Snippy (median = 54.3%) (Fig. 3d, Additional file 1: S9c and S10), since Maast is less subject to false negatives due to reference bias [13] and coverage filtering (i.e., minimum 1 × by default compared to minimum 10 × for Snippy). Because simulated reads may capture less contamination and sequencing errors than real WGS data, we repeated the evaluation with 63 short read libraries of the same species downloaded from the CGR study (*B. uniformis*, Additional file 5: Table S4). Compared to Snippy, Maast had slightly higher PPV (Maast median = 99.74%, Snippy median = 98.65%) and much higher sensitivity (Maast median = 93.3%, Snippy median = 61.7%) (Fig. 3e, f, Additional file 1: S9b, d, and S11). Higher sequencing coverage (100 x) or using paired-end reads only resulted in minor changes to the genotyping accuracy of both Maast and Snippy (Additional file 1: Figure S11, 12, and 13). We conclude that Maast is a more accurate and sensitive tool compared to Snippy, reflecting the advantages of 31-mer exact matching over read alignment for SNP genotyping, especially for sequencing data with relatively more contamination or sequencing errors.

In addition, we compared Maast to a powerful pangenome SNP calling method, Cortex [16], with the 45 *B. uniformis* strains. We used the Maast reference genome plus four additional, randomly selected genomes to generate the Cortex index. We observed that Cortex calls ~ 2.5 times more variants than does Maast. However, the runtime of Cortex was 250 times longer than Maast. We also found that genotyping results generated by Maast and Cortex were highly concordant: > 93% of the SNPs called by Maast were also called by Cortex. Thus, more SNPs can be discovered with pangenome methods compared to methods like Maast that use a single reference genome. But this additional power for SNP discovery comes at a large computational cost that prohibits applications to genotyping thousands of strains.

### Maast reveals global genetic structure of 3178 *H. pylori* isolates

**Fig. 4:**
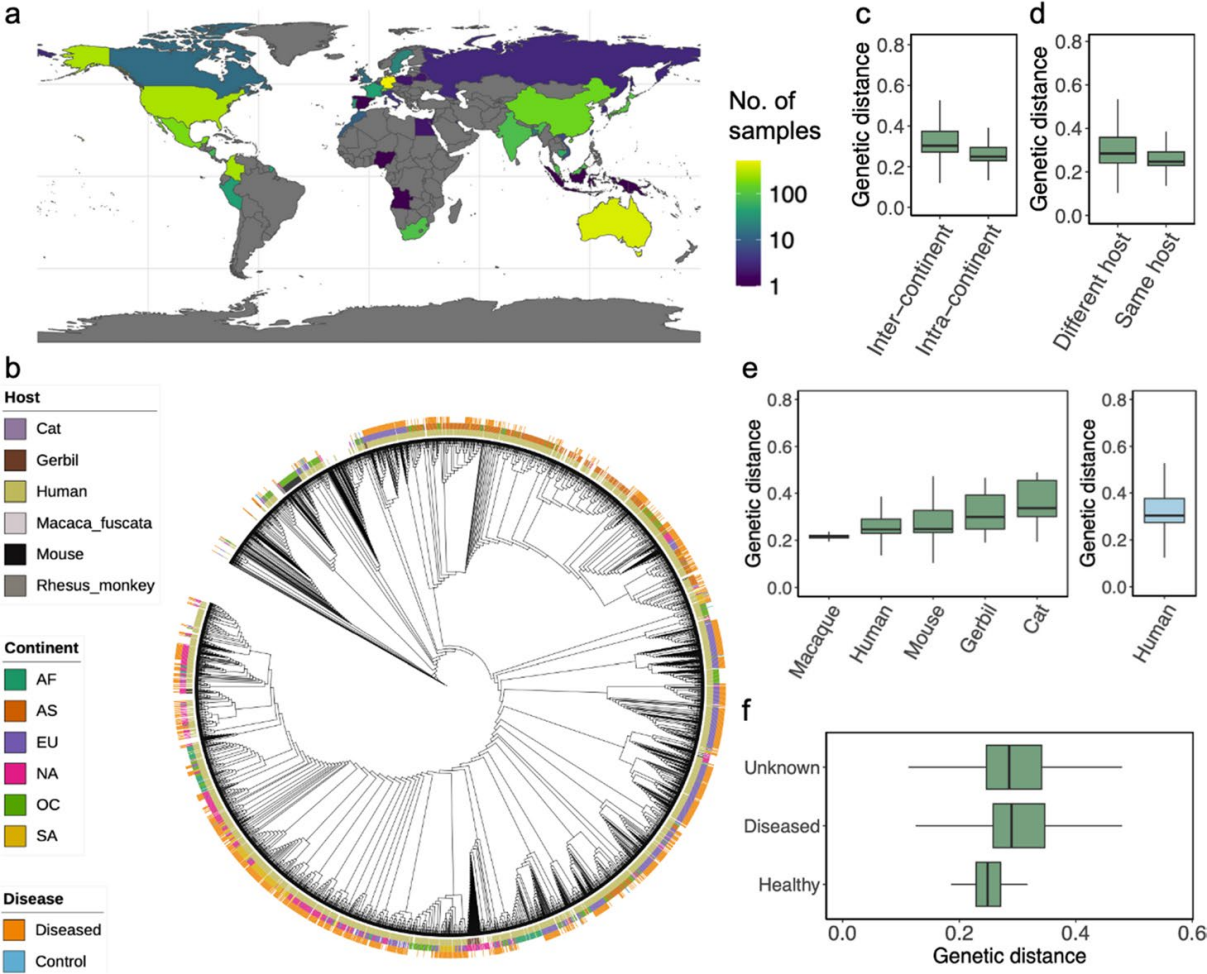
Global genetic structure of *H. pylori* genomes. **a** Geographic distribution of 3068 *H. pylori* strains across 39 countries with color indicating the number of strains from each country. **b** Strain population structure of *H. pylori* strains reconstructed from their Maast SNP genotypes. Stacked color rings indicate the host species (inner), continent (middle), and human host disease status (outer). **c**–**f** Comparison of genetic distances between pairs of strains from **c** same versus different continent, **d** same versus different host species within the same continents, **e** human versus other host species, and **f** control vs diseased human hosts. In **e**, genetic distances are calculated between pairs of strains from the same continent where one is from a human host and the other is from the indicated host species (left) or between pairs of strains from human hosts from different continents (blue box at right). The *H. pylori* strains that infected Rhesus monkeys were inoculated [19] and thus excluded

To demonstrate its scalability and utility, we leveraged Maast to analyze 3178 *H. pylori* whole-genome sequences from 39 countries across six continents (Fig. 4a, Additional file 1: Figure S8, Additional file 7: Table S6, Additional file 8: Table S7). These included isolates from five animal species in addition to humans (Additional file 1: Figure S14). Overall, we identified 74,962 common SNPs in the core genome of *H. pylori* using Maast (“Methods”), which was > 10 times more than a recent study [17]. Using these genotypes, we measured genetic distance between strains across continents and by host species, sex, and disease status (Fig. 4b, Additional file 1: Figure S15, S16). We observed clear associations between *H. pylori* genotypes and geography (Fig. 4b and c) which supported the finding that *H. pylori* has distinct populations across the globe [18]. Within the same continent, *H. pylori* strains from the same host species were more similar than those from the different host species (*p*-value < 2.2e − 16, Wilcoxon rank sum test). Interestingly, we observed *H. pylori* strains from different host species but the same continent were more similar than those from human hosts living in different continents (Fig. 4e). This is likely due to *H. pylori* populations being highly heterogenized across continents, suggesting geography is a transcending factor over host species. We also observed that genetic distances between *H. pylori* strains were on average lower (*p*-value = 2.27e − 13) between pairs of healthy human hosts compared to diseased pairs (Fig. 4f and Additional file 1: Figure S17), implying greater genomic commonality between non-pathogenic *H. pylori* strains.

Next, we analyzed 478 *H. pylori* isolates downloaded from NCBI (Additional file 9: Table S8) to assess whether we would obtain the same results using Maast with unassembled sequencing reads instead of whole genome sequences (“Methods”). For these strains, we observed the expected similarity between isolates and their unassembled WGS reads, except for a few highly similar isolates which cannot be distinguished based on common SNPs (Fig. 3g and Additional file 1: Figure S18). Next, we extended this analysis to four distinct *H. pylori* strains that had both whole genome assembly and long-read data. We found perfect clustering of assemblies with the long-read data from the same strain (Fig. 3h). Altogether our evaluation suggests that the two Maast genotyping workflows, although using distinct algorithms, are highly concordant. This means that genomic data from different sources can be pooled for analysis with Maast without assembly or other preprocessing.

### Maast enables fast tracking of SARS-CoV-2 variants

**Fig. 5:**
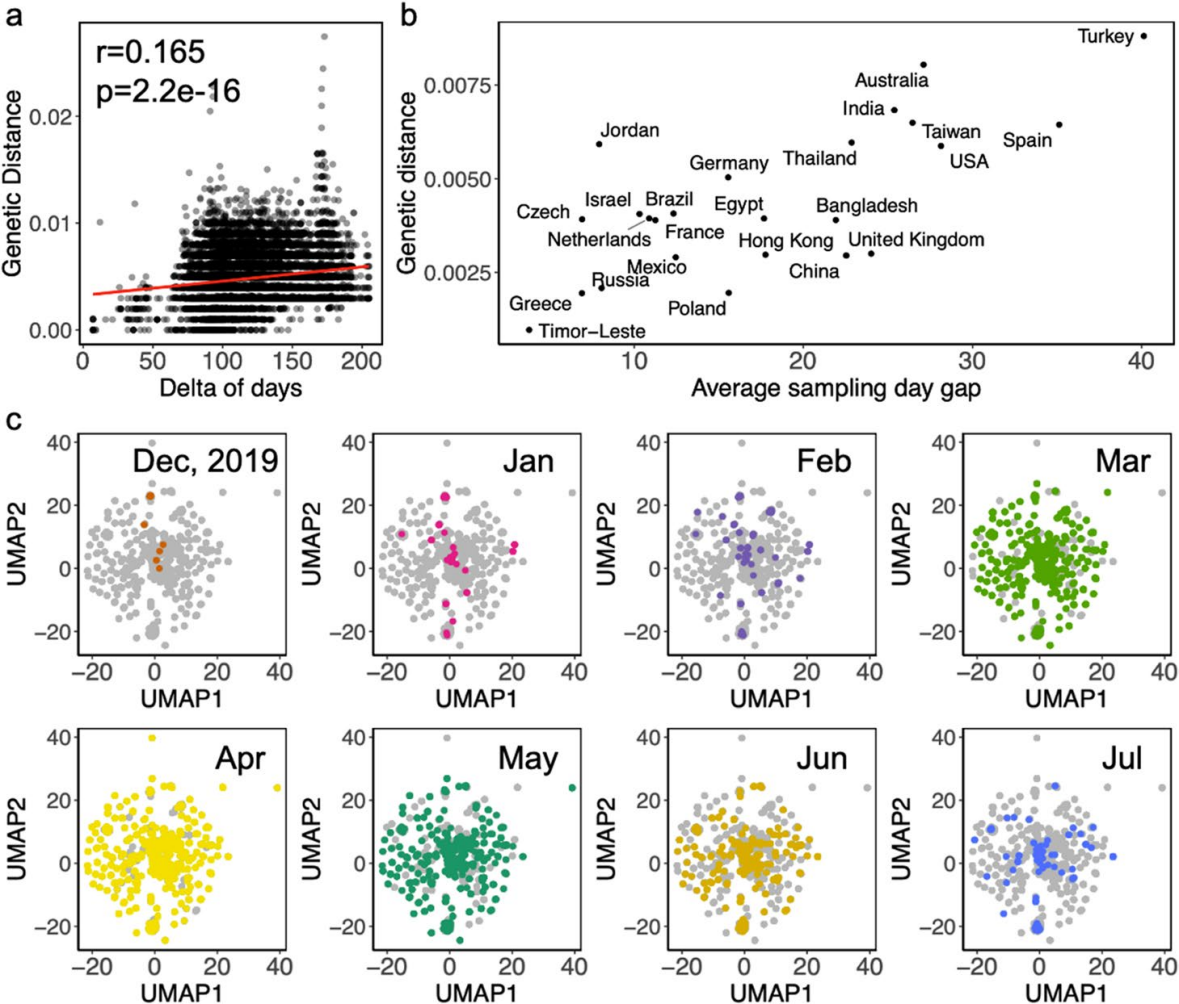
Application of Maast to track *SARS-CoV-2* diversification from Dec 2019 to July 2020. **a** Genetic distances of SARS-CoV-2 strains over time compared to one of the earliest *SARS-CoV-2* strains (Accession #: NC_045512). **b** Median genetic distance of *SARS-CoV-2* in a country is strongly correlated with average sampling day gap. **c** Subspecies structure of *SARS-CoV-2* over time. Each dot is a *SARS-CoV-2* strain, colored by sampling month with other time points in gray. Dimension reduction and visualization were performed with UMAP. Nearby samples in UMAP space have similar genotypes

To apply Maast for global pathogen surveillance, we analyzed 37,096 SARS-CoV-2 (SC2) strains, including 8734 isolate genome assemblies and 28,362 WGS read samples, collected from 60 countries over a span of ~ 200 days (downloaded from NCBI on July 19, 2020, Additional file 10: Table S9, Additional file 1: Figure S19, S20). Maast was able to process the data in < 9 h with 36 threads and a peak RAM use of 20 GB on an AWS EC2 instance (r5.16xlarge). Analyzing patterns in these genotypes, we first observed a clear divergence of SC2 strains relative to strain NC_045512 which was one of the first sequenced SC2 strains (Fig. 5a). Furthermore, we found that genetic distance between SC2 strains was slightly lower within countries than between countries (Figure S21). Within-country genetic distance differed across countries, likely due to differences in temporal adjacency of samples (Fig. 5b and Additional file 1: Figure S22). However, we also observed exceptions where geographically adjacent countries with similar temporal sampling patterns had different levels of within-country genetic distance, such as Poland and Germany (Fig. 5b and Additional file 1: S22), suggesting other underlying factors such as single versus multiple introduction sources. To track the differentiation of SC2 strains, we next used SC2 SNPs to perform dimension reduction and observed changing subspecies genetic structure over time with the emergence and fading of SC2 strain clusters (Fig. 5f). These results suggest that Maast is computationally efficient and accurate enough to be used to monitor the dynamics of the genetic structure of emerging pathogens at a global scale.

## Discussion

In this study, we present Maast, a new software tool for discovering SNPs in conspecific genomes that also includes pipelines for genotyping the panel of discovered SNPs in sequencing reads (long or short) and genome assemblies. In terms of SNP discovery, Maast is faster than existing methods due to our DynCC algorithm that identifies a subset of diverse genomes that can be used for the computationally intensive step of comparing all pairs of genomes without losing sensitivity. In fact, using these tag genomes improves our ability to identify SNPs at a given MAF and prevalence in the broader population by reducing bias due to uneven sampling of sequenced genomes. In terms of SNP genotyping, Maast uses less RAM and disk than existing methods while being about two orders of magnitude faster due to a combination of efficient data structures and novel genotyping algorithms for both genomes and unassembled reads.

The computational efficiency and accuracy of Maast enables a broad array of potential applications. In this study, we demonstrated how Maast could be used to study the genetic structure of *H. pylori*, a bacterial species with strong biogeographic patterns. We also used the early months of the SARS-CoV-2 pandemic as a case study to illustrate that Maast could be applied to emerging viral pathogens. Maast’s ability to process thousands of genomes makes it a useful tool for tracking microbial evolution in real time. By constructing and maintaining a SNP panel and k-mer database for any medically important species over time, Maast can be applied to rapidly genotype thousands of new samples without the need for genome assembly, enabling new variants to be monitored as they spread. Beyond these applications, Maast is also ideally suited for population genetic investigations of any species with many assembled or unassembled genome sequences. Its genotypes can be utilized to measure rates and patterns of selection, recombination, drift, and migration among lineages of a species.

Despite its advantages, Maast has several limitations. One challenge for SNP discovery is that Maast, like Parsnp and other methods requiring whole genome sequences, cannot be applied to species with very few high-quality assembled genomes. This is a common scenario for uncultured prokaryotic species plus many eukaryotes and viruses beyond the best-studied pathogens, although metagenome-assembled genomes and single-cell sequencing methods are closing this gap [20–22]. The main trade-off of Maast’s genotyping algorithm is that it uses SNPs discovered in an initial collection of assembled genomes and hence may fail to detect novel variants in short reads or genomes, including rare variants and SNPs that are common in lineages not well-represented in the SNP panel. This can be problematic in some settings, such as tracking the evolution of closely related pathogenic strains. Pangenome methods [16, 23] have the potential to overcome this limitation, because they utilize more of the accessory genome. However, our benchmark using the Cortex algorithm demonstrated that the computing requirements of current pangenome implementations do not permit analysis with datasets of the scale explored in this study. Accelerating these algorithms is an exciting area for future research. In the meantime, Maast enables large-scale applications to uncover the vast genetic structure of Earth’s microbiomes using common SNPs.

## Conclusions

Maast is an open-source software tool that provides a highly efficient method for discovering and genotyping common, bi-allelic SNPs using either whole-genome sequences or unassembled sequencing reads. It includes a novel algorithm that dynamically collapses thousands of genomes into a diverse, representative set of tag genomes that can be used to discover the most common SNPs with reduced computational resources and less bias than using all genomes for a species. This SNP discovery method is combined with a genotyping strategy that is a hybrid of whole-genome alignment and k-mer exact matching. Maast’s genotyping method achieves higher speed and accuracy than existing tools, scaling up population genetic analysis to an unrivaled number of strains compared to state-of-the-art methods. We have demonstrated that Maast could efficiently reconstruct the genetic structure of *H. pylori* and track SARS-CoV-2 variants during the COVID-19 outbreak, applications that involved thousands of strains across the globe. As the number of species with multiple high-quality, near-complete genome sequences continues to grow, Maast is poised to catalog this vast genetic variation.

## Methods

### Maast overview

Maast is an open-source bioinformatics tool for fast and accurate SNP genotyping from conspecific genome assemblies and sequencing reads. The development of Maast is motivated by the following observations: (1) redundant genomes are common in genome databases and these contribute little to SNP discovery while imposing a computational burden, (2) the level and pattern of redundancy vary across species, (3) the choice of reference genome affects SNP discovery, and (4) for sequencing reads, genotyping by assembly first or read alignment does not scale with increasing amounts of data.

Maast implements a catalog-first-genotype-later scheme. It was designed in order to enable genotyping to be scaled up for very large populations. Maast also synchronizes microbial genotyping for both genome assemblies and sequencing reads. The key innovations of Maast are its efficiency and novel workflow. We note that the underlying algorithms in this workflow leverage previously existing methods for whole genome alignment and k-mer exact matching.

Maast takes a set of intraspecific genomes as input and has the following major steps per species: (1) estimates the pairwise genomic distance between genomes and identifies genome clusters, (2) collapses clusters to a single tag genome per cluster, (3) picks a centroid genome as reference genome, (4) performs multiple whole-genome alignment, constructs consensus genome and calls SNPs to generate a SNP panel, and (5) leverages the SNP panel to perform in silico genotyping for genome assemblies or sequencing reads.

### Pairwise genomic distance calculation

Maast estimates the genomic distance between a pair of genomes using Mash [24] (version 2.2). Mash distance can be used to approximate whole-genome average nucleotide identity (ANI) between similar genomes (e.g., conspecific genomes). For input genomes, Maast first builds 20-mer profiles or Mash sketches using its “sketch” subcommand with default parameter except for option “-s 5000” (Additional file 1: Figure S1). Next, Maast calls the “dist” subcommand in Mash with default options to compute pairwise genomic distances.

### Identify genome clusters and tag genomes

Maast automatically determines the number of genome clusters (n) based on the user-defined minor allele frequency (MAF): n = 1/MAF. By default, SNPs are defined as genomic sites with MAF > 1%. In this case, at least 100 genomes are required to enable an effective resolution of 1%, that is 1 in 100 genomes has the minor allele. With higher MAF thresholds, fewer genomes are needed, and vice versa.

We implemented a dynamic graph algorithm (Dynamic Connected Component search (DynaCC)) in Maast to identify tag genomes based on pairwise Mash distances. The goal is to identify a minimal set of genomes that captures as much genetic variation as possible through a heuristic search. DynaCC starts with a complete graph where nodes are genomes and edges are weighted by distance. The algorithm applies a distance cutoff (d-cut) strategy to prune edges, where pairs of genomes (edges) with a distance higher than a d-cut are deemed sufficiently dissimilar and removed. By applying a stringent d-cut, many edges are deleted, which reduces the complete genome graph to connected components and isolated nodes. By doing so, Maast avoids loading a complete connected graph into RAM as well as performing operations on it, which would be computationally expensive when the number of genomes is large. A connected component here is analogous to a genome cluster which is defined as a maximum set of nodes in which a path exists between every pair of nodes, and a path is a sequence of edges which joins a pair of nodes. The algorithm identifies tag genomes as a union of hub genomes from connected components and all isolated nodes, and by default, it chooses a hub genome as the genome with the most edges in a connected component (i.e., the highest degree centrality after filtering out edges below the distance cutoff). Maast also allows users to identify hub genomes using weighted edges through the flag “–edge-weighted” as well as more sophisticated node centrality estimation methods (such as eigenvector centrality, closeness, information, betweenness, and load centrality) through the flag “–tag-centrality”. Users can also run Maast using all genomes, rather than with tag genomes, as long as they have sufficient computing power given the number of genomes.

Next, we sought to implement an algorithm that solves the problem of identifying ~ n tag genomes from N genomes, where N > n. Since the level and pattern of redundancy within species vary, an identical d-cut can result in detected n (n′) drastically different across species. When n′ is lower than the target n, SNPs are called with a de facto higher MAF, resulting in a lower sensitivity for detecting SNPs. When n′ is much higher, the benefit of computing efficiency will diminish. It is also unrealistic to predict a static d-cut per species, due to the unknown diversity of input genomes. Here DynaCC searches and determines an optimal d-cut per species dynamically with respect to the input genomes. It first uses a range factor (rf; default 1.2) designating a critical range of n (n to n*rf) that is acceptable, since the exact n may never exist in the search space. It then applies an initial d-cut (default 0.01) and if n′ is lower than n, it searches the lower bound of d-cut with an exponential decay by a factor of 10 until an n′ higher than n is found or a hard bound (default 0.000001) is reached. Between the initial d-cut and lower bound, the algorithm performs a binary search until an n′ falling in the critical range is found or the maximum searching step (default 10) runs out. When no optimal n′ is found, the algorithm settles with a suboptimal n′ which is both higher than and nearest to n. The resulting n′ tag genomes are used for the downstream workflow.

DynaCC presents two advantages for clustering whole genome sequences compared to other clustering tools, such as CD-HIT, vsearch, and MMSeqs2. First, DynaCC leverages a fast approximation of ANI, making it better suited for processing a large number of whole genome sequences. For example, we ran DynaCC, CD-HIT, vsearch (UCLUST equivalent), and MMSeqs2 on 3,646 whole genomes of *Alistipes putredinis* (Species ID: 101302). All programs were run in an identical computing environment (AWS EC2 r5.16xlarge) for a maximum of 48 h. DynaCC successfully divided these genomes into 110 clusters in ~ 3 min, while the same task took MMSeqs2 ~ 4 h. Both CD-HIT and vsearch ultimately did not run through the task: the CD-HIT main program was terminated by signal 11 (reached 498 GB RAM cap) and vsearch did not complete within 48 h. Second, DynaCC is more efficient at dividing whole genome sequences into a fixed number of clusters. Other algorithms will usually take one single distance (or similarity) cutoff as input and use this same cutoff across all species to divide genome sequences into a smaller untargeted number of clusters. DynaCC automatically searches a large space of cutoffs around a target minimum number of clusters (i.e., tag genomes) and finds an  optimal cutoff per species to generate at least the targeted number of clusters. For example, DynaCC automatically divided genomes of *A. putredinis* (*n* = 3646) and *A. rectalis* (*n* = 5214), two example species in Additional file 1: Figure S5, into ~ 100 clusters (110 clusters for *A. putredinis* and 106 for *A. rectalis*), while using a static genomic distance cutoff of 0.01 resulted in 46 genome clusters for *A. putredinis* and 2650 clusters for *A. rectalis*. In contrast, MMSeqs2 by default generated 3646 *A. putredinis* clusters and 5214 *A. rectalis* clusters, barely collapsing any redundant genomes. Thus, DynaCC rapidly prunes a large set of genomes to a fairly consistent target size across diverse and heterogeneous species. We implemented a method with this behavior because we found that it enabled efficient downstream SNP calling.

### Identify the centroid of tag genomes

The choice of reference genome is important to SNP discovery. Maast objectively picks a reference genome for each species as the centroid of tag genomes. Maast calculates all pairwise genomic distances between tag genomes using Mash. For simplicity, the tag genome with the lowest average L-1 distance to all other tag genomes is selected as the centroid genome for the species. Maast also allows users to select the centroid genome using L-2 and L-Inf distance. By default, Maast uses the centroid genome as the reference genome for multiple sequence alignments as well as SNP calling. Unless otherwise mentioned, we used the centroid genome for each SNP analysis in this study.

### Multiple sequence alignment and SNP calling

For each species, we performed whole-genome alignment by aligning each intraspecific genome to the centroid or otherwise specified reference genome using MUMmer [25] (version 4.0.0beta2) with the default parameters. Unreliable and repeat-induced alignments were removed using the delta-filter program from MUMmer with options ‘-q -r’ and the remaining alignments were then extracted using the show-coords program with default parameters. To promote the quality of multiple whole-genome alignment, we removed regions that are overly short (< 500 bp) or poorly aligned (alignment ANI < 95% of whole-genome ANI). Maast horizontally concatenates all qualified alignments and calls SNPs as sites with the following characteristics: 1) two or more nucleotides, 2) present in at least a user-supplied percentage of genomes (prevalence threshold, default ≥ 90%), and 3) minor allele frequency greater than a user-supplied value (default ≥ 1%). Finally, Maast organizes the called SNPs into a panel and outputs it in a standard VCF format. We note that Maast does not identify insertion and deletion variants (INDELs) or structural variants, even though it produces a multiple sequence alignment in a multi-FASTA format.

### Maast in silico genotyping of SNPs

For genome assemblies, Maast leverages the SNP panel from upstream steps to perform genotyping. Maast aligns each query genome, which could be non-tag genomes from SNP discovery or other additional input genomes, to the centroid genome using MUMmer (version 4.0.0beta2) with consistent quality control steps and parameters identical to those used in SNP calling. For each SNP in the panel, Maast then searches all best alignments to locate the genomic position of the SNP. If the genomic position of a SNP is found in an alignment, Maast reads and reports the allele on the query genome; otherwise, Maast reports the SNP as missing.

For sequencing reads, Maast uses a k-mer exact matching algorithm for efficient and accurate genotyping. Maast leverages the SNP panel to extract short unique genomic regions (k-mers) as probes for detecting alleles that distinguish highly similar genomes and uses these k-mers to rapidly genotype reads. For k-mer extraction, Maast identifies any 31-base SNP-covering k-mers (sck-mers) that cover (in any of the 31 bases) each of the SNP alleles. We chose 31 for k based on analyses in our previous study. For each SNP, Maast first extracts all possible 31-mers containing the SNP site from the representative genome (sck-mers for reference allele). Next, Maast extracts sck-mers by sliding a fixed-size window along the multiple sequence alignment. At any given position, the window allows extraction of n sck-mers, where n equals the number of rows in the multiple sequence alignment (i.e., the number of aligned genomes). Next, Maast reduces these n sck-mers to m unique sck-mers (m $\le$ n) and selects the one from these m with the highest frequency across the n genomes. INDELs near (within 30 bp of) a SNP could affect the number of sck-mers extracted. Maast accounts for three scenarios: no INDELs, INDELs at one side of a SNP site, and INDELs at both sides of a SNP site. When no INDELs are present, all 31 sck-mers will be extracted (note that the SNP site can be at any base on a 31-mer). When INDELs only occur at one side of a SNP site, 1 to 30 sck-mers will be extracted, depending on the location of the nearest INDEL to the SNP site. In rare cases where two or more INDELs flank a SNP site, no sck-mers will be extracted. To exclude sck-mers from repetitive genomic regions, Maast searches every candidate sck-mer in all input genomes, not only tag genomes, and removes those that occur anywhere else in any genome. We retrieved the reverse complements of all sck-mers. In this way, for every SNP site, there will be up to 62 sck-mers targeting the reference or alternative allele.

To facilitate efficient storage and match of sck-mers, we use 64-bit integers to represent the sck-mers with 00 for “A,” 01 for “C,” 10 for “G,”and 11 for “T”and discarded the sck-mers with wildcards (e.g. “N”). We sort sck-mers in colex order which is essentially a reflection of lexicographic order that reads a sequence from the right to the left (versus left to the right). For example, given a set of unsorted sequences, {“214”, “123”, “134”, “125”}, the corresponding lexicographic order is {“123”, “125”, “134”, “214”} and the colex order is {“123”, “214”, “134”, “125”}. Colex order enables fast matching of suffixes, because related suffixes are near each other in a list. Specifically, we build an *l-*index to quickly locate all sck-mers that share a given suffix by simply pointing to the first and last entries for each suffix. This is possible because sck-mers that end with a suffix *s* of length *l* will occupy consecutive entries in the colex sorted list. We empirically selected *l* = 36 as default. For each input read, Maast first breaks it down into 31-mers and encodes them into 64-bit integers. Our goal is to find exact matches of these 31-mers with sck-mers and the following exact-match algorithm is implemented: (1) look up query suffix in the *l-mer* index, if found, (2) examine all sck-mer entries identified by the *l-mer* index one by one and report exact matches. After generating all k-mers in each metagenomic sequencing read, Maast recruits an L-bit index (L-index; last L bits/suffix of encoded k-mer) to locate a bucket of pre-sorted sck-mers in the database containing all possible exact matches to the full k-mer. The algorithm invokes a sequential search for exact matches between the full k-mer and only the sck-mers in this bucket. We note that both alleles may be detected for one SNP site due to the presence of more than one strain, de novo mutations, or sequencing errors in the sample. In the default output of Maast, if multiple alleles are detected, the counts of both alleles will be reported. Further processing of the alleles is delegated to downstream analysis. Throughout this study, we assumed there was only one dominant strain per sample and when multiple alleles were detected for a SNP site within one sample, we proceeded with the major allele (allele frequency > 0.5) and ignored the minor allele for all analyses unless otherwise mentioned. The count of reads matching an allele will be incremented by one as long as at least one k-mer was detected in the read being processed.

The output format of in silico genotyping is similar for both genome assemblies and sequencing reads. It uses a concise table-shaped format for its output, in which every row represents a bi-allelic SNP site. Each row has exactly 8 fields: species, SNP ID, contig, contig position, allele 1, allele 2, and coverage of allele 1 and coverage of allele 2. Currently, the algorithm only supports bi-allelic SNPs which are the vast majority of the SNPs.

### UHGG whole genome sequences and species

We downloaded genome sequences from the Unified Human Gastrointestinal Genomes [2] (UHGG) at http://ftp.ebi.ac.uk/pub/databases/metagenomics/mgnify_genomes as of September 2019. The UHGG database is a very large collection of gut microbial whole genome sequences, which are originally from both isolate assemblies and metagenome-assembled genomes (MAGs). The inclusion of MAGs from diverse human populations and geographic locations is critical for capturing natural genetic variation within human gut species. From the UHGG database, we selected 146 species, each with 200 or more high-quality (completeness >  = 90% and contamination rate <  = 5%) whole genome sequences, which accounted for a total of 109,365 genomes. Twenty-nine of them had more than 1000 genomes, and *Escherichia coli_D* (species id: 102506) had the most genomes (*n* = 6645).

### Comparing Maast to related methods

We compared Maast to several other methods in a series of simulations and data analyses designed to evaluate different aspects of computational performance and accuracy. For SNP calling in whole genome sequences, we included two methods, Parsnp and kSNP, which represented two distinct methods. Parsnp reports SNPs in the coordinates of the specified reference genome, and kSNP does not. We selected ParSNP as opposed to other methods for calling SNPs through multiple whole genome alignment, such as Mugsy [26] and Mauve [27], due to its improved computing performance and similar accuracy [10]. For SNP calling in short reads, we included Snippy, a representative of a widely used three-step workflow: short reads first are aligned to reference genomes (read mapping), mapped reads are then piled up for counting coverage per site (pile-up), and SNPs are called from site coverage profiles (SNP calling). Specifically, Snippy (https://github.com/tseemann/snippy; June 2020) uses BWA mem [28] (version 0.7.17-r1188) for read mapping, samtools [29] (version 1.12) to sort and filter BAM files, and freebayes [30] (version v1.3.5) for pile-up and SNP calling. Here, we chose Snippy for comparisons and analyses as it was recently reported to be the best method overall among methods that follow a similar strategy [31]. To control for possible biases in using the Maast tag genomes as representative genomes, we instead used the UHGG representative genomes with all methods unless otherwise specified.

In all comparisons, we ran Maast, Parsnp, kSNP, and Snippy with default parameters except for a flag of “-c” for Parsnp to indicate all genomes are from the same species. For all paired-end samples, we processed only forward reads (fastq 1) for the simplicity of comparison and analysis. We skipped a sample if it did not have a forward read sample as extracted from a SRA file using fastq-dump in the SRA toolkit. However, we note that using the reads of both directions can effectively increase the coverage, which thus should be recommended especially for species with low abundance.

### Computing performance evaluations

We compared Maast with kSNP3 and Parsnp to evaluate its computing performance on SNP calling with whole genome sequences. We downloaded a total of 5214 high-quality genomes for one of the most well-sequenced bacterial species, *Agathobacter rectali*, from UHGG genome colletion (Additional file 3: Table S2). We randomly sampled 500, 1000, or 5000 genome sequences and ran all three methods on these genome sequences to assess the scalability of these methods. Since both kSNP3 and Parsnp failed to run through all the *A. rectalis* genomes, we downloaded high-quality genomes from UHGG for two other well-sequenced species, i.e., 3646 genomes for *Alistipes putredinis* and 3170 genomes for *Bacteroides_B dorei* (Additional file 3: Table S2), and repeated the evaluation with both species to ensure the observed issue was not limited to a single species. To enable the comparison between Maast, Parsnp, and kSNP as well as the performance evaluation of each method, we simulated genomes to control the level of genomic divergence within a species. For the simulation, we took a random genome (UHGG id: GUT_GENOME143713) from *Agathobacter rectalis* as a simulation template and randomly selected 10,000 genomic positions on the template to be SNP sites. For each simulated genome, 10% of the SNP sites (1000 SNPs) were randomly selected to be non-reference alleles, which we inserted in silico. For simplicity, we only simulated bi-allelic SNPs. To evaluate the scalability of the methods, we used three levels of input size (500, 1000, and 5000 genomes).

To evaluate SNP genotyping in short reads, we downloaded a total of 63 samples of short reads for an arbitrary species (*Bacteroides uniformis*) from the Culturable Genome Reference (CGR) study [32]. Each sample provided a distinct sequenced strain of that species. Altogether these samples accounted for a total of ~ 150 million reads.

We evaluated the computing performance of Maast and other tools on an AWS EC2 instance with the following specifications: AWS r5.16xlarge, 32 physical CPU cores (64 vCPU), Intel 8175 M CPU @ 2.50 GHz, 512 GB RAM and EBS gp2 RAID array providing 13,600 Mbps bandwidth. We measured both speed and peak RAM consumption using the GNU time (version 1.7) command with option “-v”.

To ensure a fair comparison between different methods, all methods were run on all of the input data with the same reference genome whenever one was used. Only the step of SNP calling was evaluated; if a method had downstream steps such as SNP tree-building we did not benchmark these. Each method was run using all cores of an environment whenever possible.

We manually terminated the running of any tool after both tools had been running over a designated maximum running time window (48 h) and had not finished. When terminated, we estimated the speed of Parsnp and kSNP with the maximum time use of 48 h and projected peak RAM use linearly as a function of the number of genomes.

For the example species of *Bacteroides uniformis*, the end-to-end runtime breakdown of Maast is ~ 7 min for picking tag genomes and generating SNP panel, ~ 13 min for building sck-mer database, and ~ 19 min for genotyping 2746 whole-genome assemblies or ~ 90 s for genotyping 63 samples of whole-genome sequencing short reads.

### Accuracy evaluation of SNPs from simulated and downloaded reads

To evaluate the accuracy of SNP genotyping by Maast in short reads and to compare it to Snippy, we simulated reads from whole genome sequences. For these simulations, we downloaded a total of 45 isolate genomes for a species (*Bacteroides uniformis*) from the CGR study. These genomes were cultivated from fecal samples of healthy humans and characterized as non-redundant and high-quality draft genomes. We used InSilicoSeq [33] (version 1.4.2) with the options “–model HiSeq” to simulate up to 5.3 million reads per genome with Illumina length and error characteristics. This generated two paired-end read files each containing ~ 1 million 126 bp-long reads from each genome. For simplicity, we proceeded only the forward reads. We used genome coverage of 15 x for simulations by randomly drawing reads from the simulated metagenomes. The number of reads required for a level of coverage c was estimated by the following formula: number of reads = c × genome length / 126. For example, to provide a 15 x coverage for a genome with a size of 6 M bp, a rough number of 714,285 (15 × 5,000,000/126) 126-bp reads are needed. To complement the simulations above, we extended the accuracy analyses to the reads simulated from the same genomes at 100 × coverage as well as the 63 samples of short reads used in the computing performance evaluation. For these analyses, the reads were processed as both paired-end reads, in which forward and reverse reads were both supplied and aligned as pairs, and as single-end reads, in which forward and reverse reads were merged and supplied as a single file.

To ensure a fair comparison, we ran Maast and Snippy on both the simulated and downloaded reads with default parameters and the same high-quality reference genome (i.e., UHGG species representative genome) which was arbitrarily selected. We did not use the centroid genome as the reference genome here to control possible biases. Our evaluations focused on the correct identification of SNPs and on the accuracy of the alleles in the typed SNPs. For each strain, ground truth genotypes were determined by aligning the whole sequence of that strain to the reference genome. We focused our evaluations on the SNPs that were potentially able to be genotyped by both Maast and Snippy, i.e., SNPs in the Maast panel. True positives (TP) and false positives (FP) were the correct and incorrect genotypes compared to ground truth; false negatives (FN) were sites with no genotype or an incorrect genotype. In this way, we conveniently calculated the Positive predictive value (PPV) as the ratio between the sum of TP sites and the sum of all reported sites and the sensitivity as TP/(TP + FN) or the ratio of TP sites to the total number of sites in the Maast SNP panel. These values were calculated for each method on non-reference sites only as Snippy only reported them.

### Reconstruction of genetic structure of* H. pylori*

To show Maast is powerful for exploring genetic structure within microbial species, we downloaded a total of 3524 whole genome sequences of *H. pylori* from NCBI and PATRIC [34] websites as of March 2021. We kept 3428 high-quality genomes (completeness >  = 90% and contamination rate <  = 5%) as determined using checkM, which included 1672 and 1756 genomes from NCBI and PATRIC, respectively (Additional file 7: Table S6). We ran the SNP calling module of Maast with default parameters (site prevalence > 0.9 and MAF > 0.01) on the whole genome sequences, resulting in 107 tag genomes and a panel of 275,116 SNPs. We then ran the database building module of Maast with default settings and generated a sck-mer database with 3,566,180 sck-mers and 74,962 SNPs that were covered by at least one sck-mer.

Next, we sought to determine whether the common SNPs discovered and typed by Maast could be used for biogeographic analyses of diverged *H. pylori* strains. To maximize the number of strains in this analysis, we downloaded an additional 1522 short-read samples from NCBI SRA as of April 2021, each representing a distinct strain. We then combined these short-read samples together with 1756 PATRIC genomes to form one of the largest *H. pylori* strain collections (*n* = 3178) to date with diverse host and geographic information (Additional file 8: Table S7). We did not include NCBI whole genome sequences due to less richness in metadata. We ran Maast on these strains and used the RAxML [35] algorithm to calculate pairwise genetic distance as well as construct a phylogenetic tree based on concatenated alleles of SNP sites genotyped. We uploaded the resulting RAxML tree file to the iTol [36] website for visualization. To account for possible bias due to less covered SNPs, we applied two filters to both the SNPs and strains: (1) we excluded a SNP from this analysis if it was present in fewer than 5 strains, and (2) we excluded a strain if it had < 1000 genotyped SNPs or < 50% of total SNPs genotyped.

To further validate consistency between Maast SNP genotyping of whole genome sequences and short reads, we identified 478 *H. pylori* strains that had both raw whole-genome sequencing reads and assembled genomes available in NCBI records (Additional file 9: Table S8). We used similar steps to run Maast on these strains and reconstructed a phylogenetic tree.

### Tracking early outbreak of SARS-CoV-2

We analyzed SARS-CoV-2 strains from the early outbreak. In contrast to the long evolutionary history of *H. pylori*, genomic tracking of SARS-CoV-2 was quite recent and extensive, which provided a good example to evaluate how well Maast could be used to track the genetics of a burst of highly similar genomes. We downloaded a total of 37,096 SARS-CoV-2 strains from diverse geographic regions (Additional file 1: Figure S19), including 8734 whole genome sequences from the NCBI *SARS-CoV-2* Data Hub as well as 28,362 short read samples available from the NCBI SRA as of July 2020 (Additional file 10: Table S9). As expected, the Mash distances between SARS-CoV-2 genomes were extremely low due to the short evolution history (Additional file 1: Figure S20). To effectively differentiate these strains, we included rare SNPs in this application by running Maast with site prevalence > 0.95 and MAF > 0.001 on the whole genome sequences of SARS-CoV-2, which resulted in 1077 tag genomes and a panel of 1114 SNPs. With Maast, we built a sck-mer database, which included 128,416 sck-mers and 1045 SNPs that were covered by at least one sck-mer. Then, we genotyped SNPs for the rest of the strains with Maast and computed SNP-based genetic distance and a RAxML tree as in the *H. pylori* analysis.

To visually track the genetic change of SARS-CoV-2 over time, we identified the major allele (allele with the highest frequency across strains) of each SNP, generated a binary matrix of major allele presence/absence per strain, and performed dimension reduction on this matrix with UMAP (umap package version 0.2.3.1) in R. We plotted all strains in the resulting UMAP coordinates. We visually identified 1,073 outliers (< 3% of total strains) and removed them from the plot. To account for possible bias due to less covered SNPs, we applied two filters to both genomes and metagenomes: (1) we excluded a SNP in both genomes and metagenomes if it was present in fewer than 5 metagenomes, and (2) we excluded a metagenome if it had < 1000 genotyped SNPs or < 50% of total SNPs genotyped.

### Supplementary Information

**Additional file 1.** Supplementary Figures S1 to S22.

**Additional file 2: Supplementary Tables S1.** Summary of intra-specific genomes and common SNPs for 146 microbial species associated with the human gut.

**Additional file 3: Supplementary Tables S2.** List of UHGG genomes for 3 CGR species that were used for evaluating the computational performance of Maast.

**Additional file 4: Supplementary Tables S3.** Detailed computational performance of Maast, Parsnp and kSNP based on running each tool on 500, 1000 and 5000 genomes.

**Additional file 5: Supplementary Tables S4.** List of accession numbers for the whole genome assemblies and sequencing reads of *B. uniformis*.

**Additional file 6: Supplementary Tables S5.** Detailed comparison of SNP calling accuracy between Maast, Maast + NRC, kSNP and Parsnp.

**Additional file 7: Supplementary Tables S6.** List of accession numbers for 3,428 whole genome assemblies of *H. pylori*.

**Additional file 8: Supplementary Tables S7.** Accession numbers and metadata of 3,178 *H. pylori* strains.

**Additional file 9: Supplementary Tables S8.** List of 475 *H. pylori* strains with paired whole genome assemblies and sequencing reads.

**Additional file 10: Supplementary Tables S9.** Accession numbers and metadata of 37,096 SARS-CoV-2 strains downloaded from the NCBI *SARS-CoV-2* Data Hub and SRA.

**Additional file 11.** Review history.

## Data Availability

All described datasets are publicly available through the corresponding repositories. Genome assemblies and whole genome sequencing samples are available at UHGG, NCBI, and PATRIC database with accession numbers in supplementary tables: genome assemblies for *Alistipes putredinis*, *Bacteroides_B dorei*, and *Agathobacter rectalis* (Additional file 3: Table S2), and both genome assemblies and whole genome sequencing samples for *Bacteroides uniformis* (Additional file 5: Table S4), *Helicobacter pylori* (Additional files 7, 8 and 9: Table S6, S7, and S8) and SARS-CoV-2 (Additional file 10: Table S9). Maast is written in C +  + and Python, and it is released as open-source software under the MIT license. The source code and documentation of Maast is available on Zenodo and GitHub (https://github.com/zjshi/Maast [37, 38]).
