# Supplementary material for: Maast: genotyping thousands of microbial strains efficiently
Source: Genome Biol. 2023 Aug 10;24:186. doi: 10.1186/s13059-023-03030-8 (PMC10416524; doi:10.1186/s13059-023-03030-8)

Additional file 1: Supplementary figure S1-22 for “Maast: genotyping thousands of microbial strains efficiently”

Zhou Jason Shi^1,2^, Stephen Nayfach^3,4^ and Katherine S. Pollard^1,2,5^

^1^Chan Zuckerberg Biohub, San Francisco, CA, ^2^Gladstone Institutes, Data Science and Biotechnology, San Francisco, CA, ^3^Department of Energy, Joint Genome Institute, Walnut Creek, CA, ^4^ Lawrence Berkeley National Laboratory, Environmental Genomics and Systems Biology Division, Berkeley, CA, ^5^University of California San Francisco, Department of Epidemiology and Biostatistics, San Francisco, CA

**Supplementary Figure legends**

**Figure S1. Choosing default Mash sketch size for Maast.** Violin plot shows the distribution of Spearman correlations between whole genome ANI and Mash distance across 146 bacterial species. Correlation is calculated between all pairs of genomes in each species and the median is plotted. Three different Mash sketch sizes (1,000, 5,000 and 10,000) are evaluated.

**Figure S2. Constructing a reference panel of SNPs and genotyping sequencing reads using k-mer exact matching.** (a) Scheme whereby a Multiple Sequence Alignment is built from pairwise genome alignments. Each line represents a conspecific genome assembly. Each rectangle represents an aligned region identified through pairwise genome alignments. The lightning bolt symbol indicates SNP sites. (b) Scheme whereby SNP-covering k-mers (sck-mers) are extracted from multiple sequence alignments. Each colored rectangle represents a SNP site and the cross color indicates distinct alleles. (c). Conceptual workflow of the k-mer exact match algorithm in Maast. All sck-mers have been binary encoded, pooled, and sorted in Colex order. The L-bit suffixes of sck-mers is used to build an index (L-index; green) whose entries point to consecutive records in the sck-mer table (cyan). Efficient exact-matching of k-mers from metagenomes starts with extracting all k-mers present in each sequencing read (input). To determine what they are, the range of entries in the L-index corresponding to each L-bit hit is queried with an exact-match algorithm. Dashed black arrows indicate processes occurring during database development, and solid gray arrows indicate the Maast genotyping workflow.

**Figure S3. Flowchart of DynaCC algorithm for collapsing redundancy in the collection of whole genome sequences.** The workflow is presented sequentially from top to bottom in three steps (grey boxes).The algorithm (a) first determines the acceptance range of number of tag genomes using the user-defined minor allele frequency threshold and a range factor (>1), (b) determines the search space of distance cuts (d-cuts) that can produce an acceptable set of tag genomes, and (c) identifies the best d-cut that produces the number of tag genomes in the acceptance range or as close as possible using binary search (white box). The algorithm terminates without output if no d-cut can be found to produce the number of tag genomes higher than the lower bound of the acceptance range. Although unlikely, it is possible that a good d-cut is accepted in step (b), which results in a successful early exit.

**Figure S4. Intraspecific diversity varies across 146 human gut species.** For each species, intraspecific diversity is indicated by the median Mash distance between pairs of conspecific genomes.

**Figure S5. Species vary in their genomic redundancy.** (a) When the same d-cut (0.01) is applied to all 146 human gut species, the species vary substantially in their number of clusters with a single-linkage clustering strategy due to different levels of genomic redundancy. Circle size indicates the number of clusters identified by Maast. If levels of redundancy were invariable across species, the ratio of the number of clusters versus the number of genomes of each species would be constant. The opposite is observed here as many pairs of species with similar numbers of genomes have drastically different numbers of clusters. Arrows point to two example species (*Agathobacter rectalis* and *Alistipes putredinis*) with different redundancy levels. (b) Rarefaction curves of common SNP (minor allele frequency >= 1%, prevalence >= 90% across intraspecific genomes) discovery for two example species: *A. rectalis* (upper, high redundancy, 46 clusters based on Mash) and *A. putredinis* (lower, low redundancy, 2650 clusters). SNP discovery increases with the number of genomes and then levels off for both species. SNP discovery levels off much more quickly for *A. putredinis*, underscoring how genomic redundancy limits SNP discovery at a given MAF threshold. The top 1,000 genomes of each species with highest quality (i.e., completeness and contamination) were included for the rarefaction analysis. The curve is made by counting the number of SNPs that can be discovered using down-sampled sets of fewer genomes from 10 to 1,000 genomes increasing by 10 genomes at each step. Each down-sampling was repeated 10 times and the mean value is shown.

**Figure S6. Relationship between Mash distances and SNP discovery with Maast.** Distribution of intra-specific Mash distances for eight example species, including (a) *Anaerotignum sp000436415* (100177), (b) *Sutterella wadsworthensis_B* (101361), (c) *Succinivibrio sp000431835* (100412), (d) *Akkermansia muciniphila* (102454), (e) *Akkermansia muciniphila_B* (102453), (f) *Alistipes shahii* (100003), (g) *Faecalibacterium prausnitzii_K* (species id: 101300) and (h) *Phascolarctobacterium faecium* (103439). Species in the upper and bottom panel are the ones with a high and low level of tag-only SNPs, respectively. Species in the same column have a similar number of genomes. The level is estimated as a fraction of all SNPs that are discovered with tag genomes but not with all genomes.

**Figure S7. The number of SNPs identified in the same eight species as Figure 2c.** The total number of SNPs identified in pairwise genome-to-genome alignments and the number of these that are found in the SNP panel that was called with tag genomes (green) or with all genomes (grey). The color of the x axis label indicates the level of redundancy in the conspecific genomes of the species: high (red) versus low (blue).

**Figure S8. The median proportion of SNPs from a genome pair that are present in a reference SNP panel.** Each genome pair consists of a reference genome and a conspecific genome. The reference SNP panel is generated with a similar SNP calling workflow using tag genomes (green) or all input genomes (gray). The left panel included all genome pairs and the right panel shows the top 20 most divergent pairs, which are the pairs generating the most SNPs. The color of the y axis label indicates the level of redundancy in the conspecific genomes of the species: high redundncy (red) or low redundancy (blue).

**Figure S9. Accuracy comparison between Maast and Snippy.** Individual simulated (a and c) and (b and d) isolate sequencing samples are shown. (a and b) False positive rate at SNP sites in simulated reads (a) and isolate sequencing samples (b). (c and d) Sensitivity across the simulated reads (c) and isolate sequencing samples (d) is calculated as the probability of detecting SNPs present in the isolate genome. Only variable sites are included in the calculations.

**Figure S10. Comparison of Maast and Snippy genotyping accuracy at non-reference alleles of SNPs based on simulated whole-genome sequencing.** Both Maast and Snippy were run with default settings. (a and b) Positive predictive value (PPV) comparison, where true positives are genotype calls that match the genome. (c and d) Sensitivity is the probability of detecting genotypes present in the SNP panel generated with Maast (tag genomes, left), all input genomes (middle), or the union of these two panels (right). The analysis is based on short reads (a and c) simulated at 15x coverage from isolate genomes with sequencing error and (b and d) downloaded from isolate whole-genome sequencing projects. Both Maast and Snippy were run with default settings. Left panels are identical to the corresponding panels in Figure 3c-f and are included here for side-by-side comparison.

**Figure S11. Alternative ways to quantify Maast genotyping accuracy. (a)** Comparison of Maast genotypes from simulated forward and reverse reads. Concordance is shown as the Jaccard similarity between each pair of samples. **(b)** Comparison of Maast genotyping accuracy with and without including reference alleles. We compared performance estimated two ways: using both reference and non-reference alleles (all) versus using only non-reference alleles (variant). Comparisons were performed based on both simulated (left) and downloaded (right) whole-genome sequencing reads. SNPs in the Maast SNP panel were called in forward reads only by running Maast with default settings. (upper) Positive predictive value (PPV) comparison, where true positive discoveries are genotype calls that match the genome. (lower) Sensitivity from simulations with the Maast SNP panel as reference. Sensitivity is the probability of detecting genotypes present in the genome.

**Figure S12. Comparison of Maast and Snippy genotyping accuracy at non-reference alleles of SNPs based on downloaded whole-genome sequencing.** SNPs in the Maast SNP panel were called in short reads downloaded from isolate whole-genome sequencing projects based on forward reads only (left) and paired-end reads (right). Both Maast and Snippy were run with default settings. (upper) Positive predictive value (PPV) comparison, where true positive discoveries are genotype calls that match the genome. (lower) Sensitivity from simulations with the Maast SNP panel as reference. Sensitivity is the probability of detecting genotypes present in the genome. Left panels are the same as the corresponding panels in Figure 3e and f and are included here for side-by-side comparison.

**Figure S13. Comparison of Maast and Snippy genotyping accuracy at non-reference alleles of SNPs at different simulated sequencing coverages.** SNPs in the Maast SNP panel were genotyped based on short reads simulated from isolate genomes with sequencing error at 15x (left) and 100x (middle and right) coverage. Short reads simulated at 100x coverage were processed as both single (middle) and paired end (right) reads. Both Maast and Snippy were run with default settings. (upper) Positive predictive value (PPV) comparison, where true positive discoveries are genotype calls that match the genome. (lower) Sensitivity from the simulations with the Maast SNP panel as reference. Sensitivity is the probability of detecting genotypes present in the genome. Left panels are the same as the corresponding panels in Figure 3c and d and are included in order to enable side-by-side comparisons.

**Figure S14. Geographic distribution of *H. pylori* strains by host species.** For each host species (cat, mouse, gerbil, rhesus monkey and macaca fuscata), the number of strains is indicated (colors) across 39 countries.

**Figure S15. *H. pylori* population structure is not associated with host gender.** (a) Strain population structure of *H. pylori* reconstructed from Maast SNP genotypes. Stacked color rings indicate host gender. (b) Pairwise genetic distances between strains from human hosts that are of same or different gender.

**Figure S16. *H. pylori* strain diversity is elevated in diseased hosts*.*** Strain population structure of *H. pylori* reconstructed from Maast SNP genotypes. Stacked color rings indicate the disease status, the diagnosis of inflammation, ulcer and cancer of the human host of *H. pylori* strains.

**Figure S17. *H. pylori* strain diversity as a function of host species and geography.** Pairwise genetic distances between *H. pylori* strains from (a) the same or different host species, (b) human hosts from the same or different continents, (c) specific host species and (d) diseased or healthy hosts. (c and d) only included strains from the same continent.

**Figure S18. Pairwise genetic distances between *H. pylori* strains from the same and different data sources.** Two types of data source are compared, including whole genome assembly and sequencing reads.

**Figure S19. Distribution of Mash distances of 8,734 sequenced SARS-CoV-2 strains.**

**Figure S20. Distribution of SARS-CoV-2 WGS projects across countries.** Only countries with >30 projects were shown.

**Figure S21. Distribution of SARS-CoV-2 WGS projects over time in different countries.** Comparison of genetic distances between pairs of SARS-CoV-2 strains from (a) same versus different countries and (b) same versus different months.

**Figure S22. Genetic distances of SARS-CoV-2 strains across countries.** Left panel shows the distribution of sampling day of SARS-CoV-2 strains. Right panel shows the distribution of genetic distance of strain pairs in each country.

**Supplementary Figures**

Figure S1


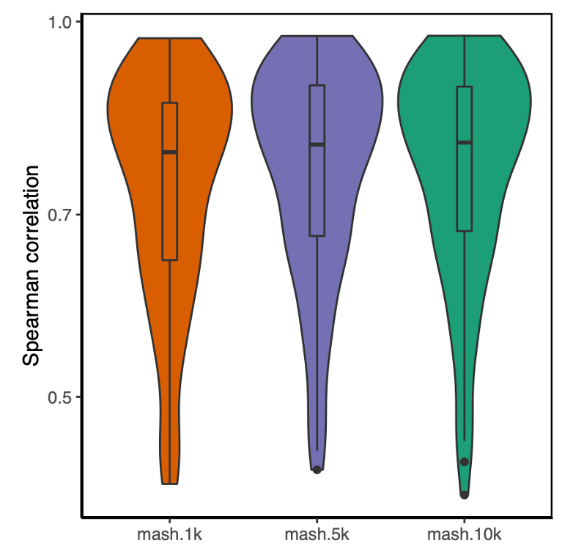


Figure S2


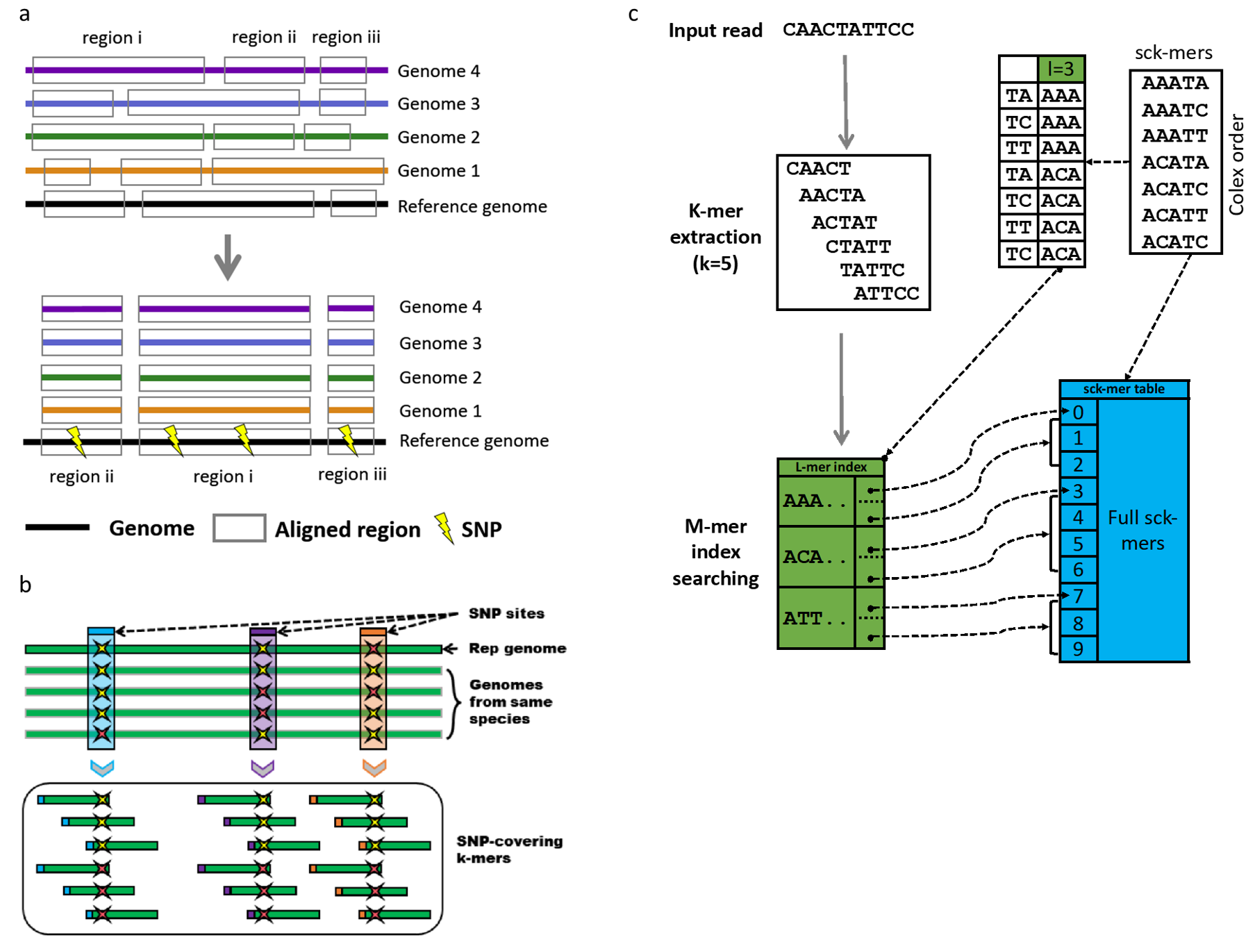


Figure S3


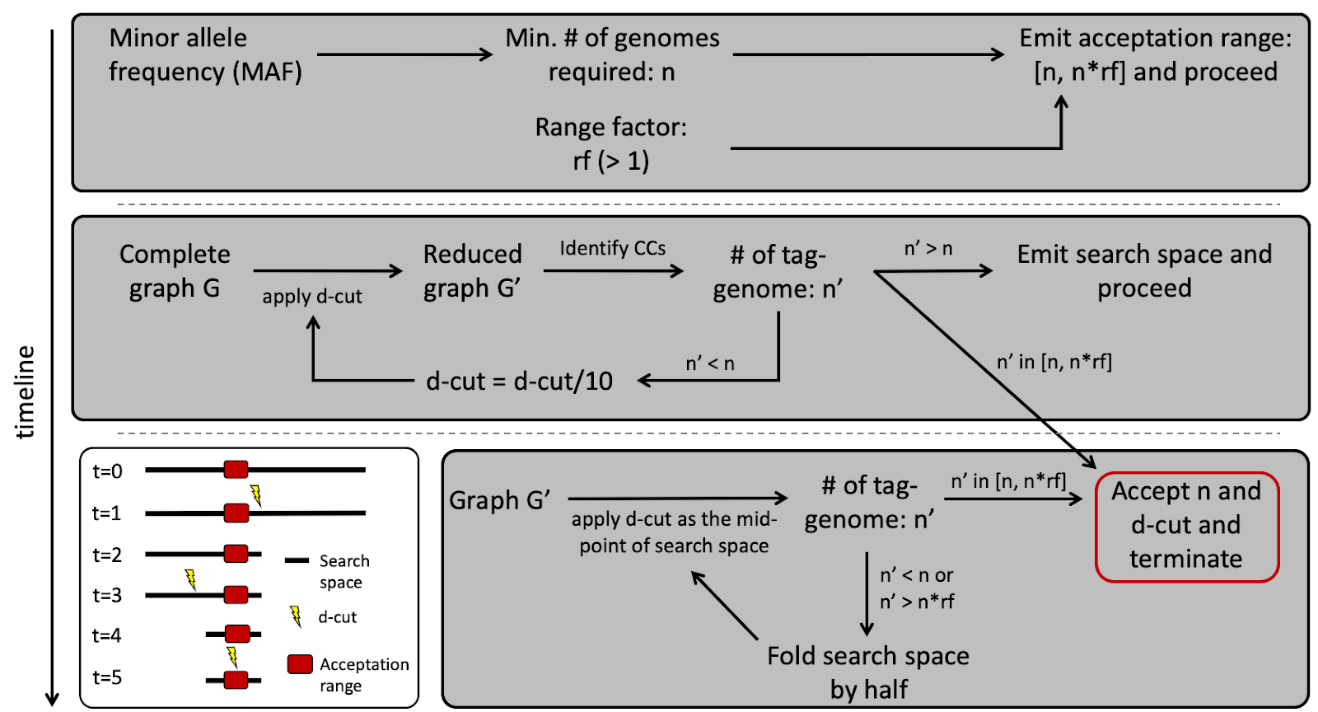


Figure S4


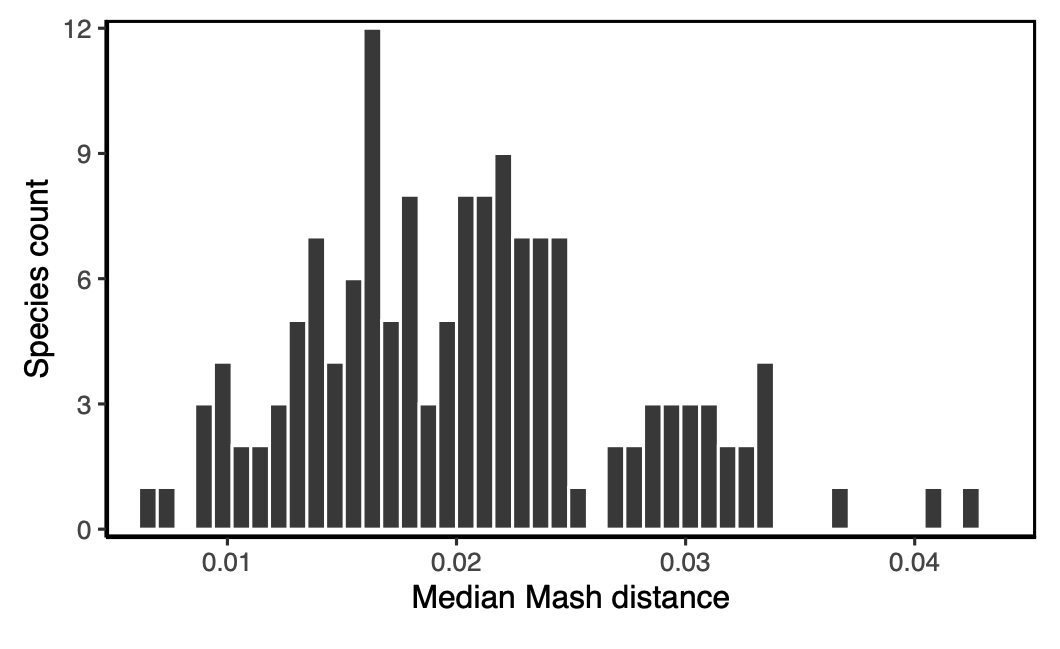


Figure S5


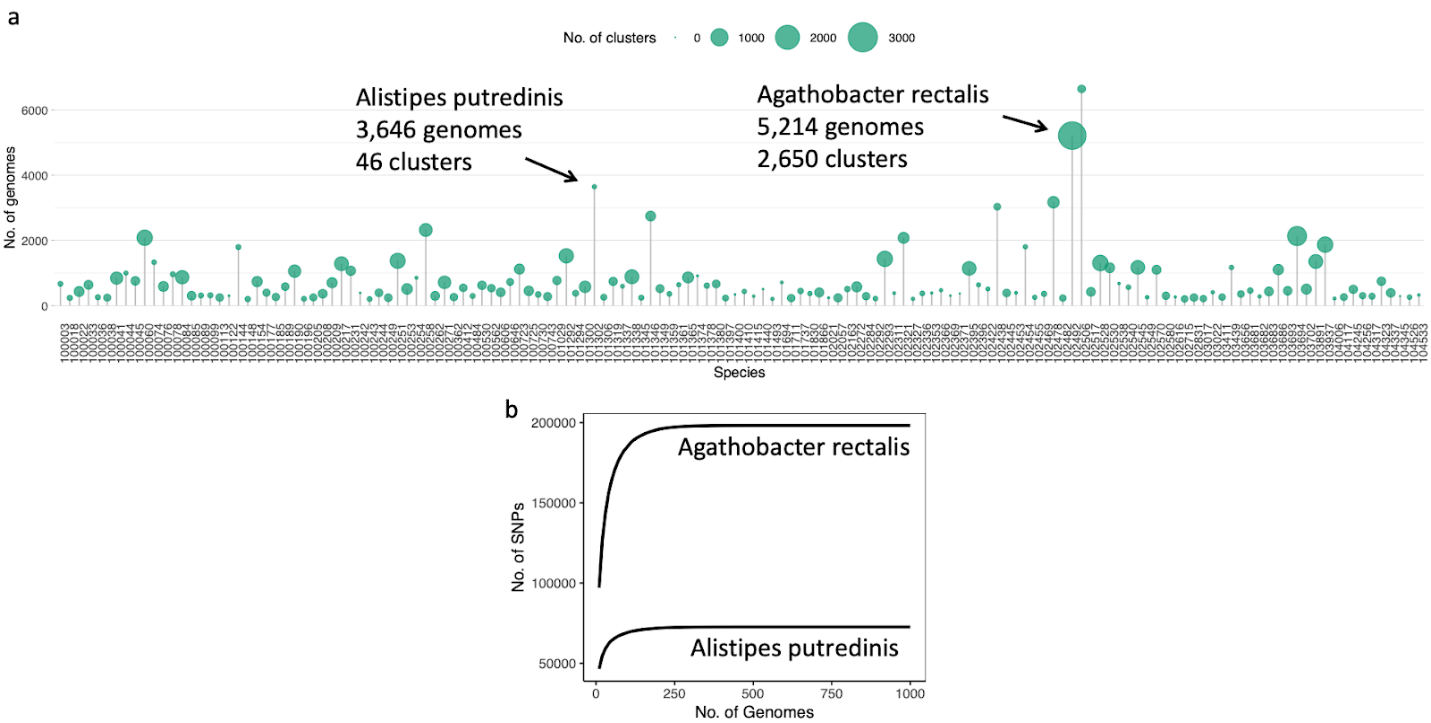


Figure S6


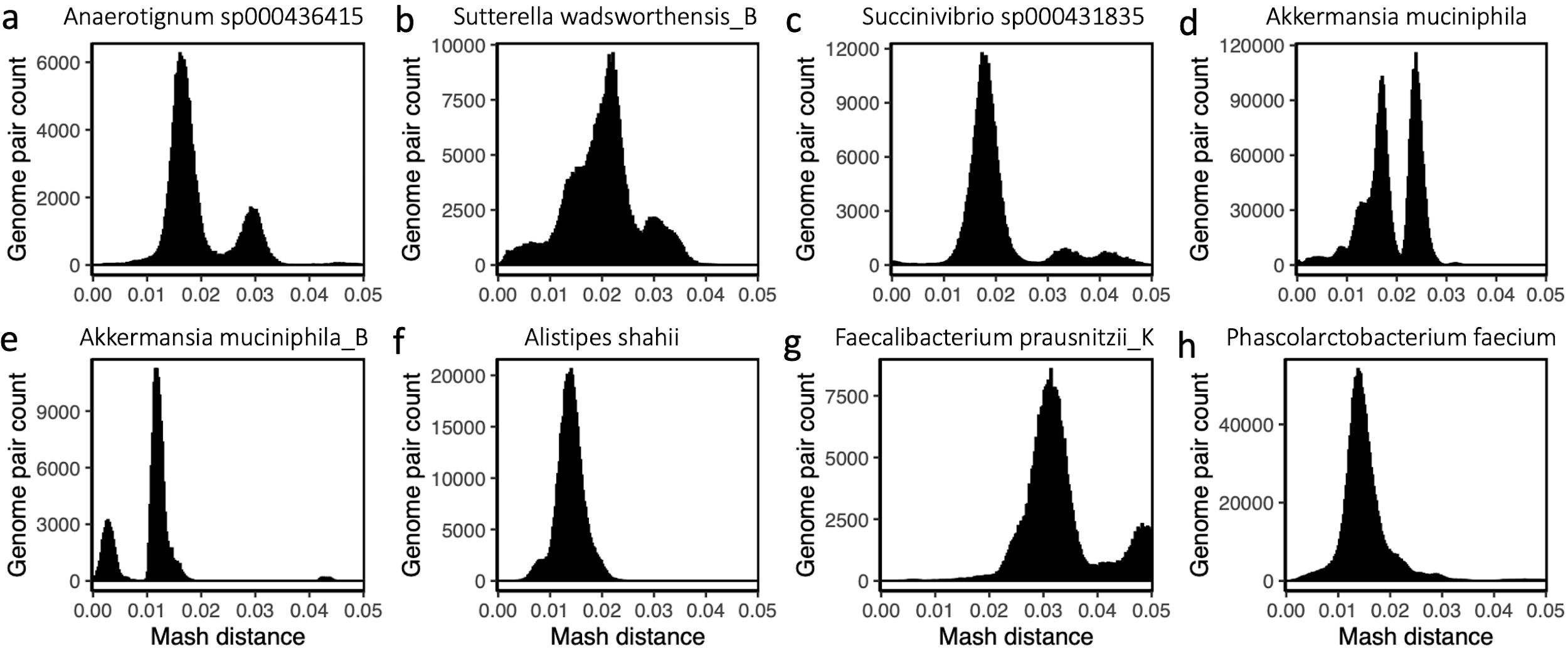


Figure S7


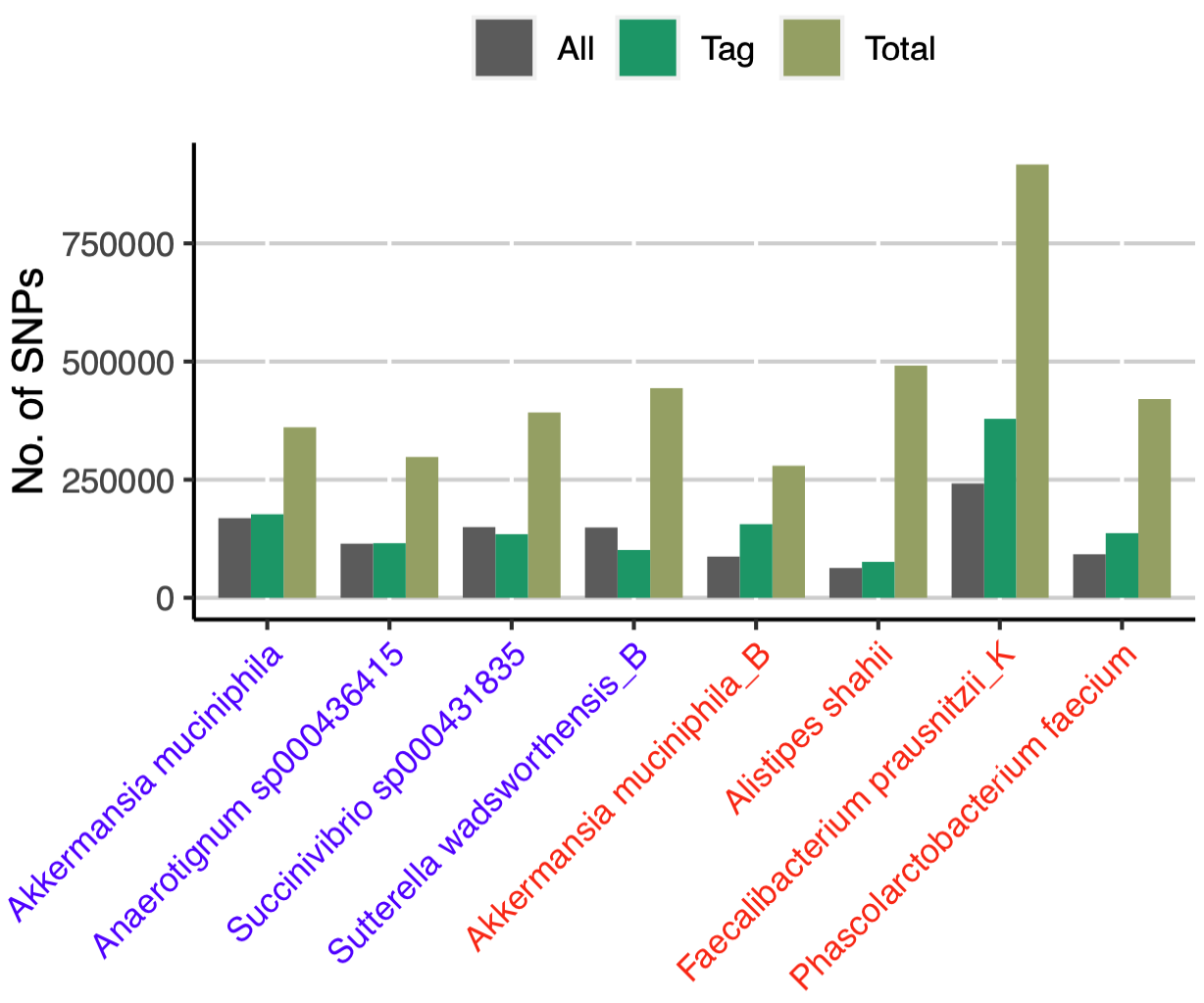


Figure S8


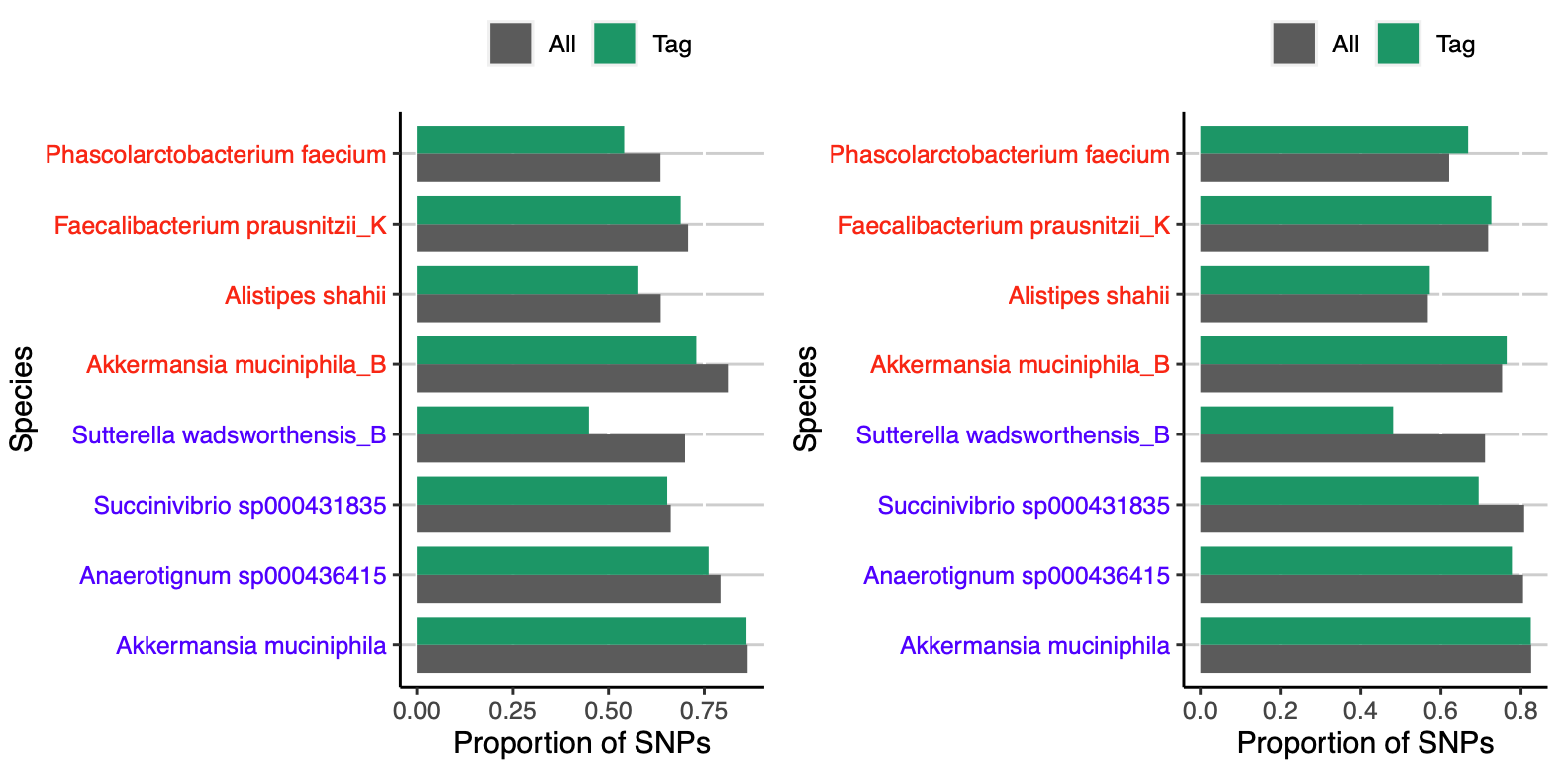


Figure S9


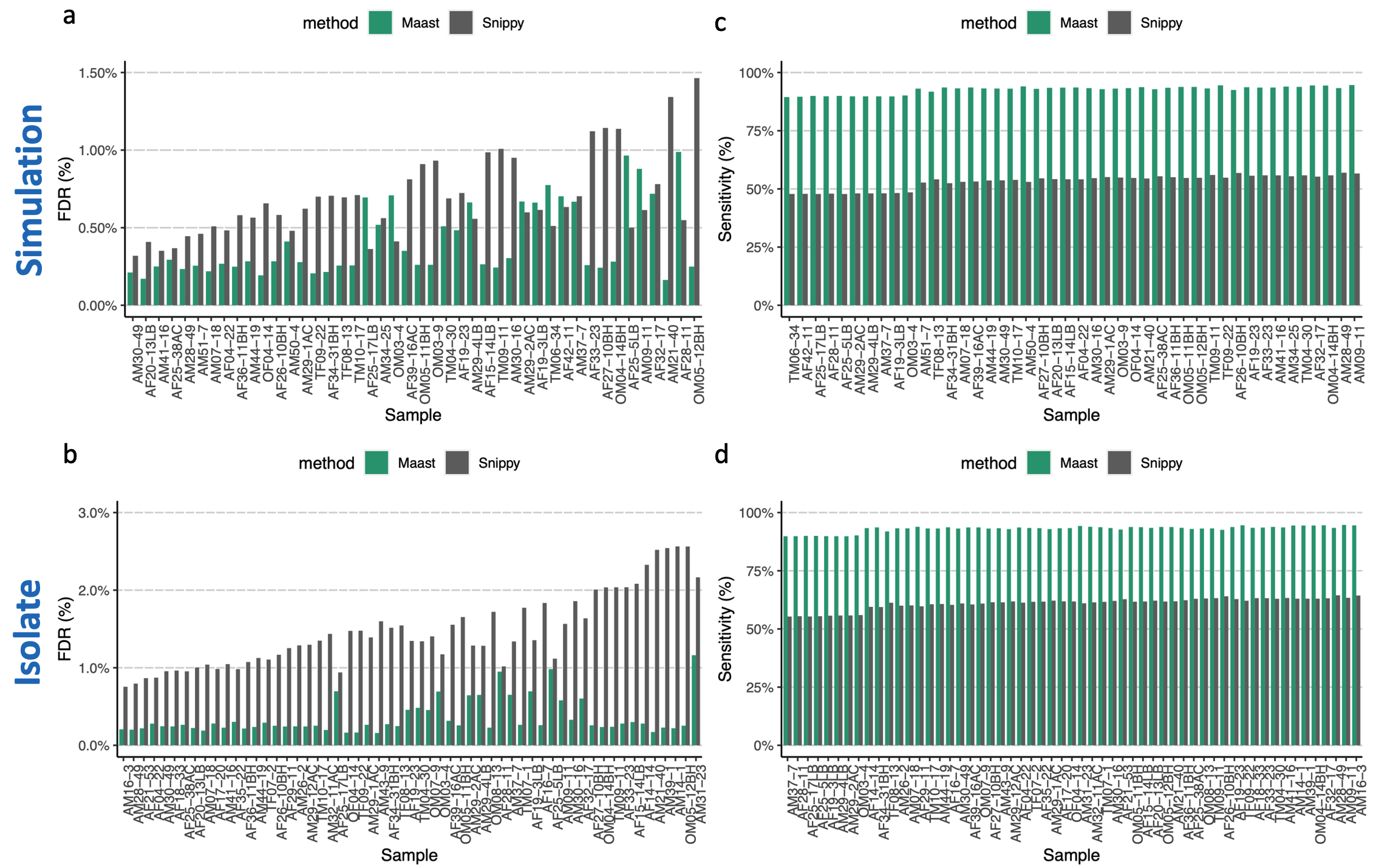


Figure S10


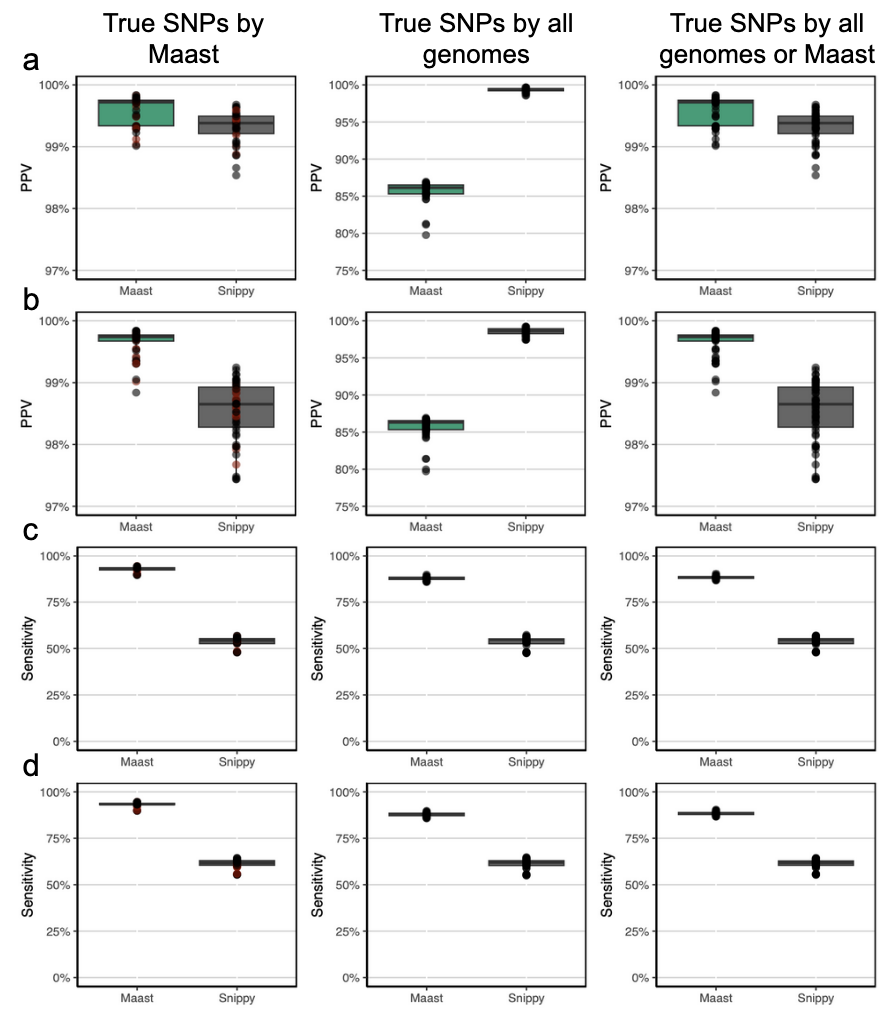


Figure S11


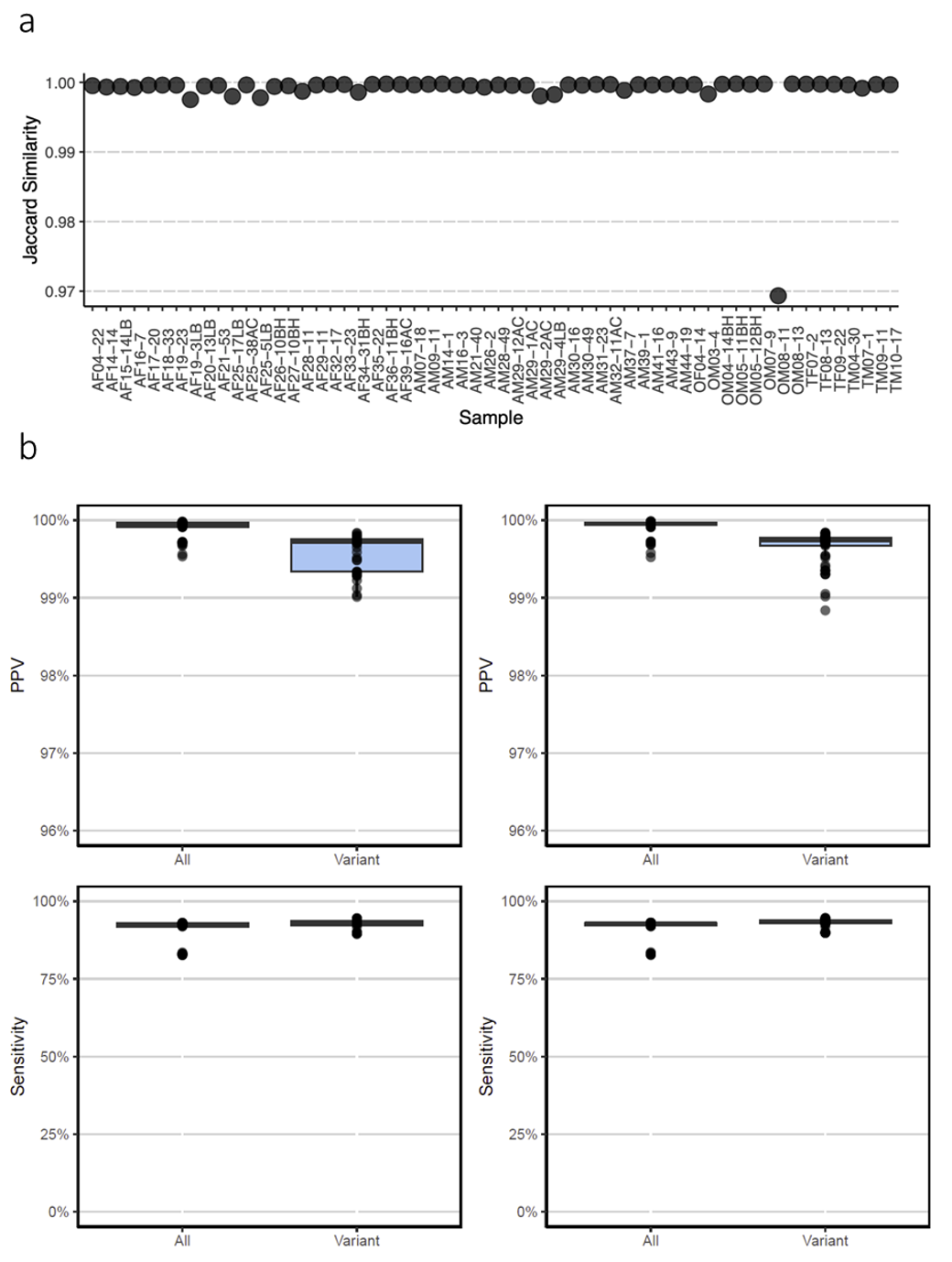


Figure S12


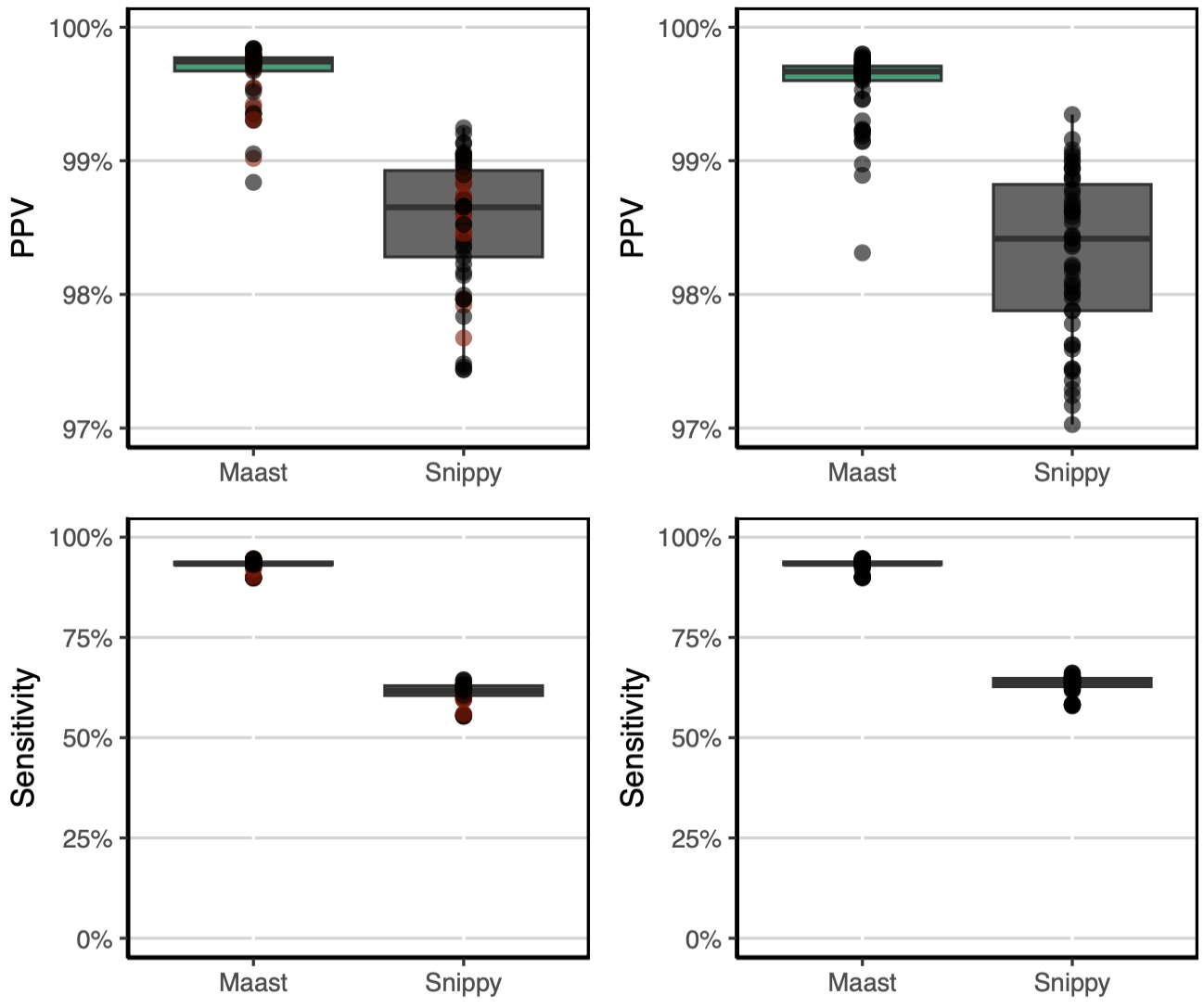


Figure S13


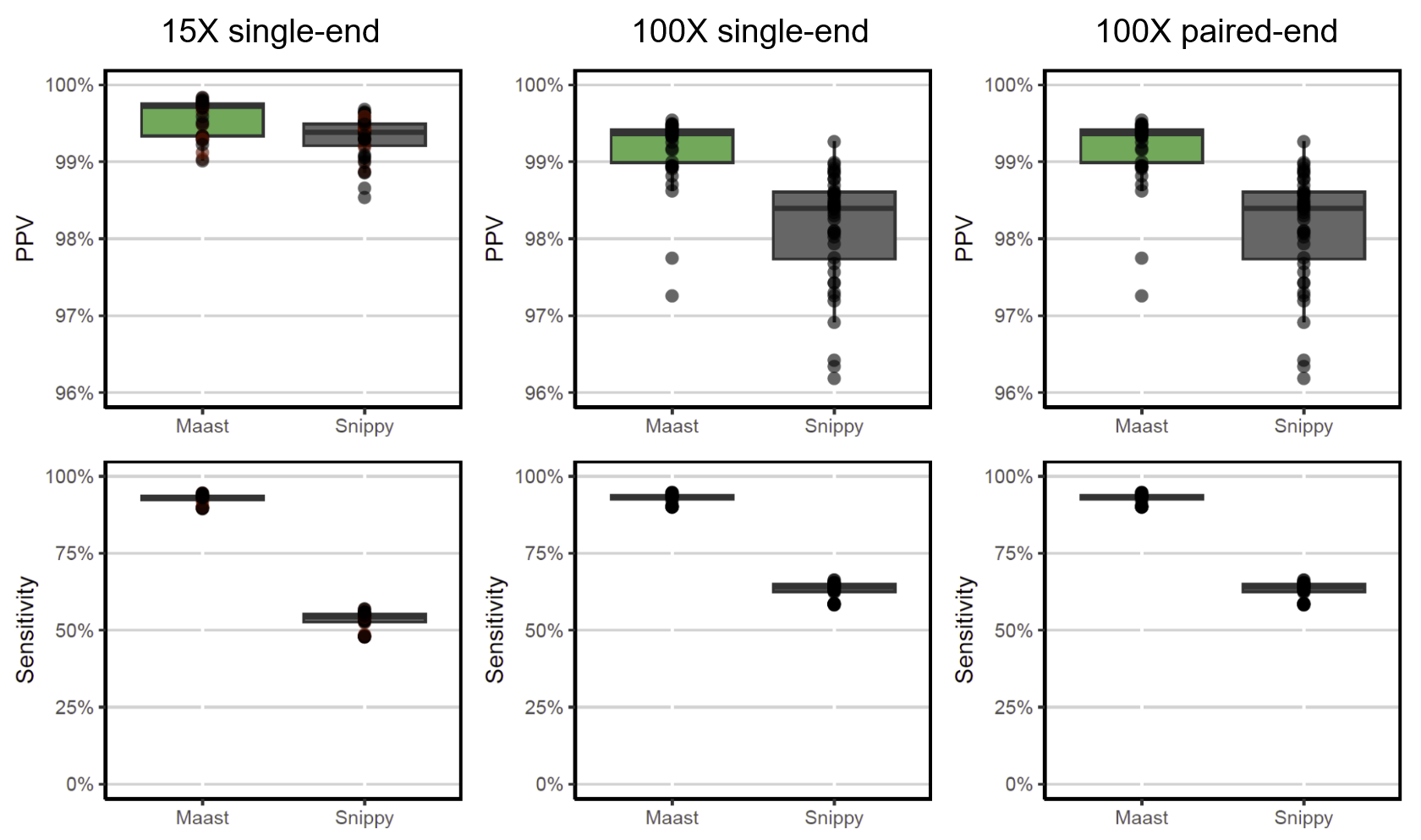


Figure S14


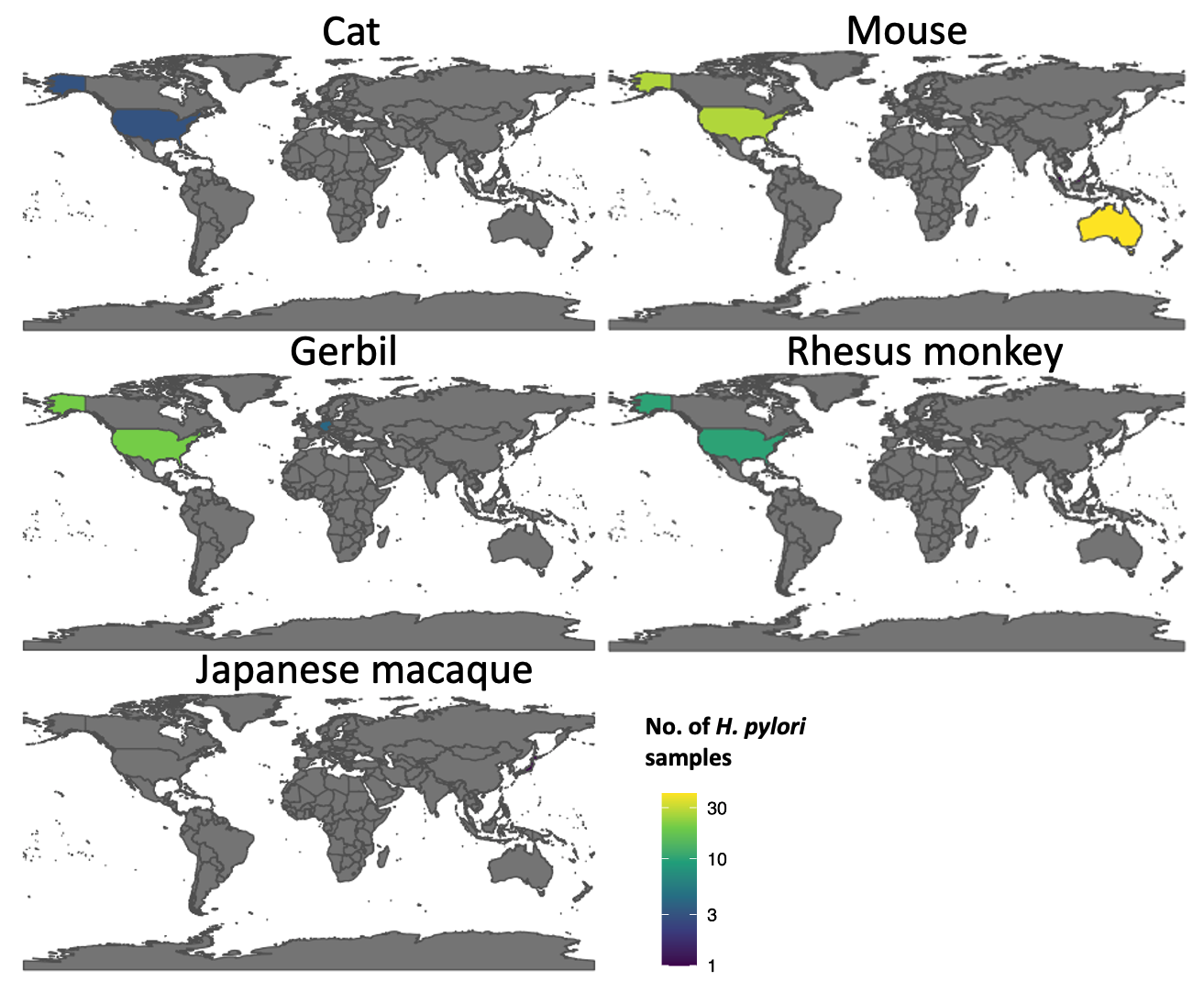


Figure S15


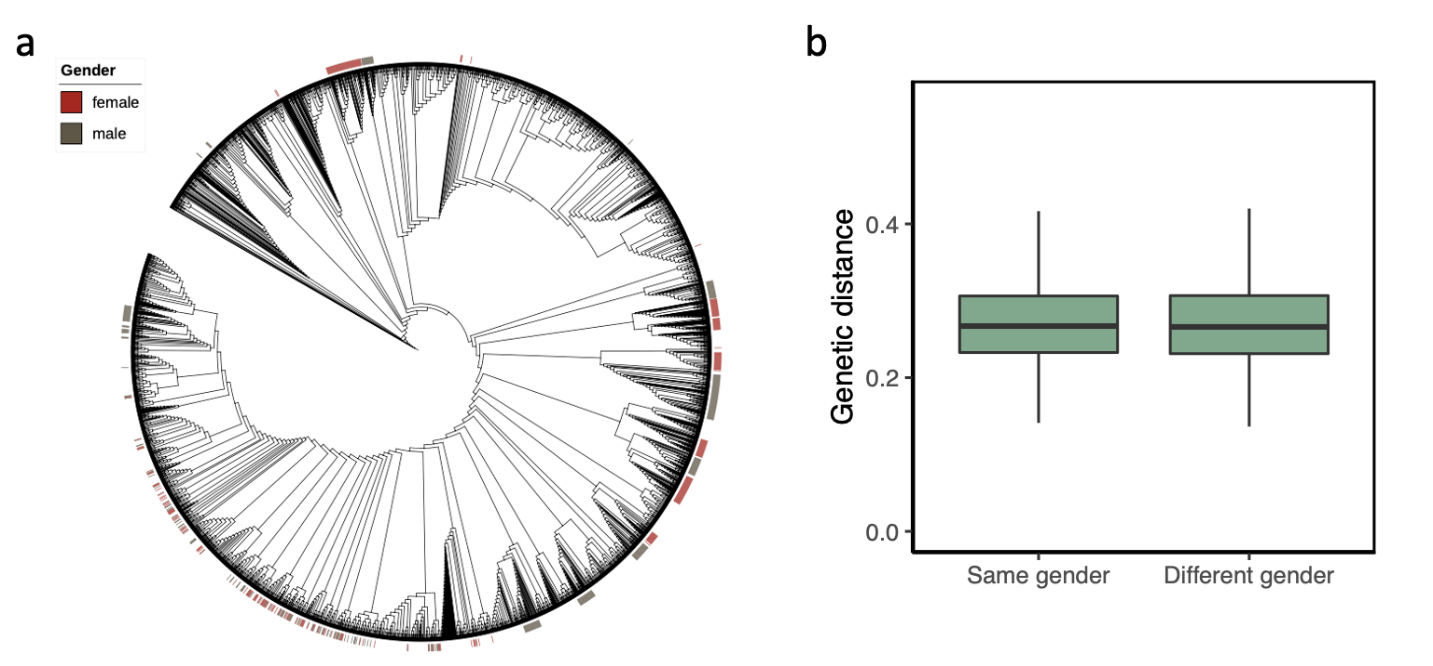


Figure S16


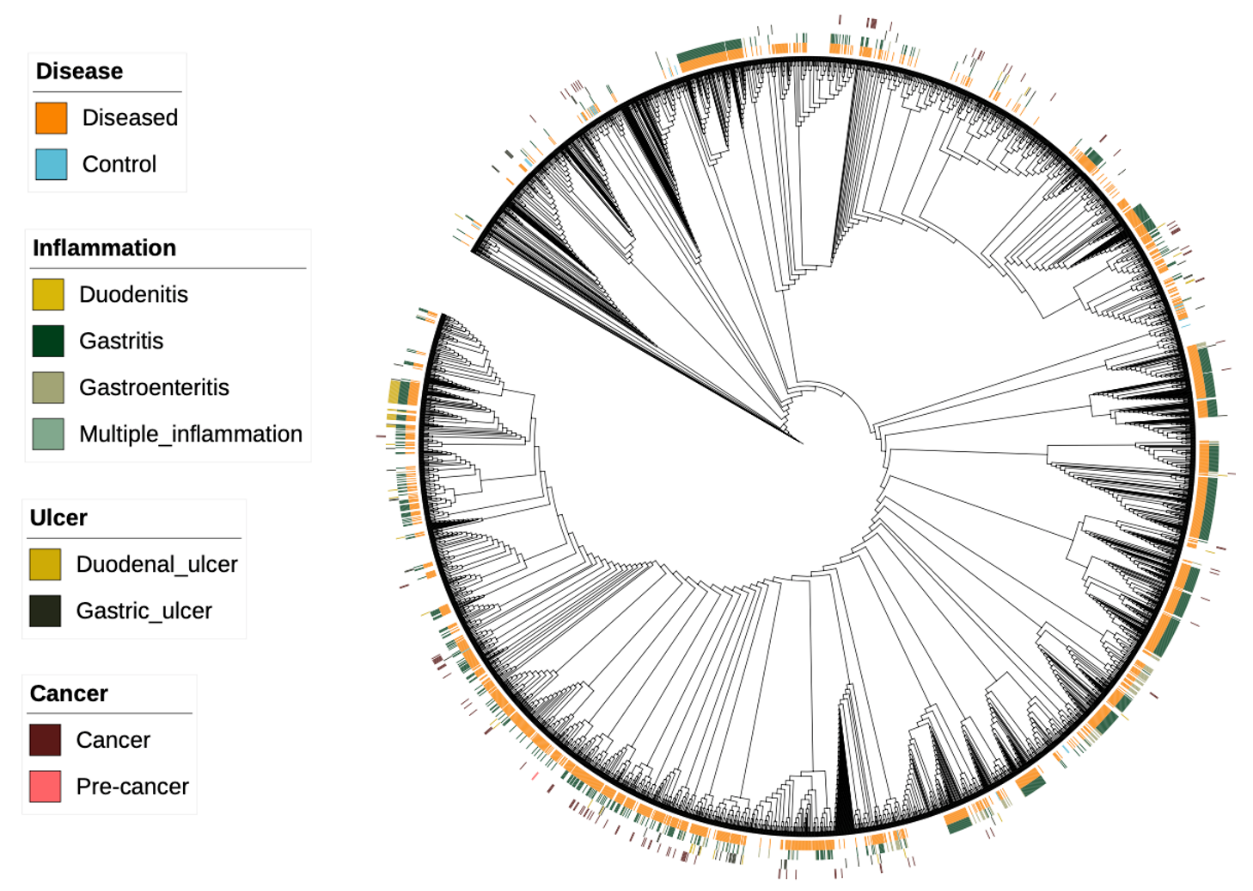


Figure S17


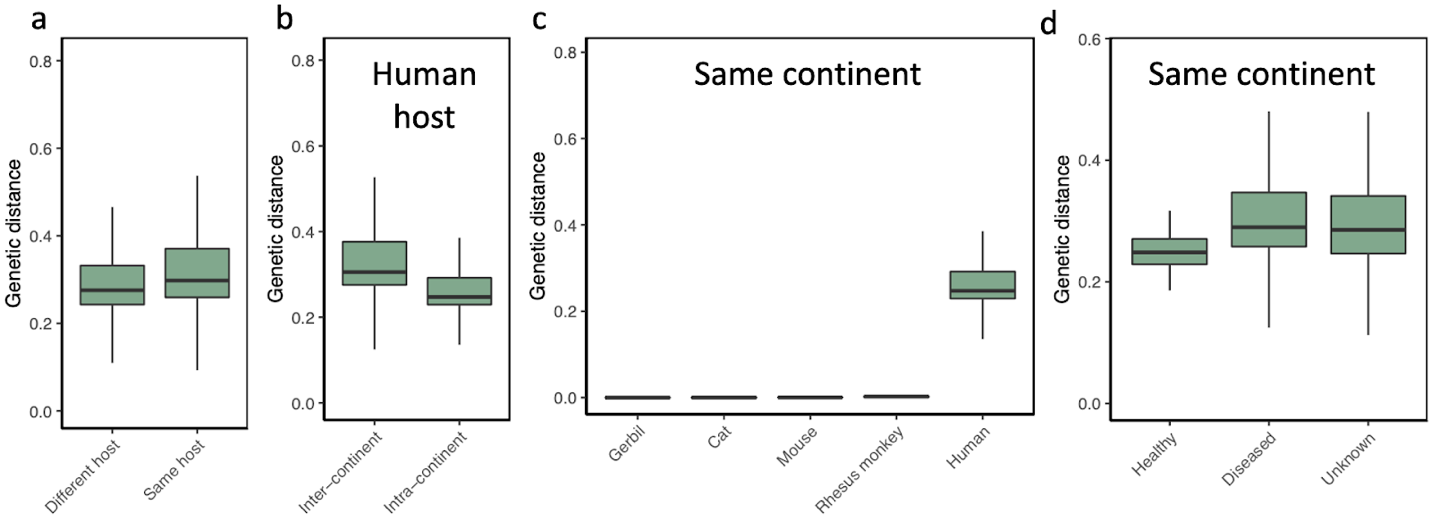


Figure S18


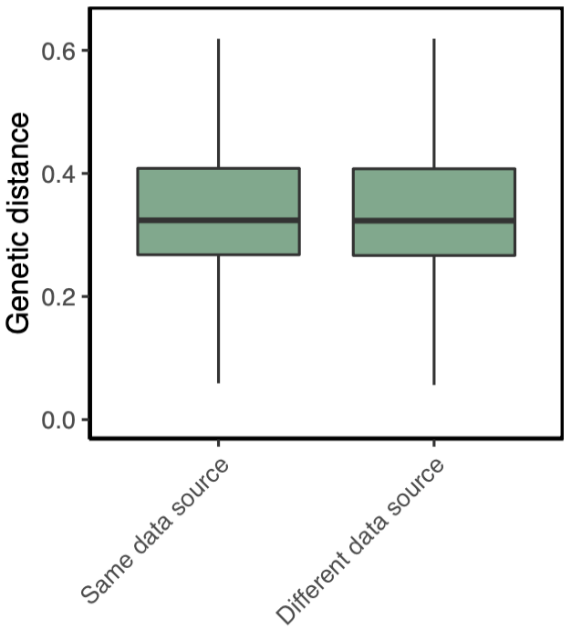


Figure S19


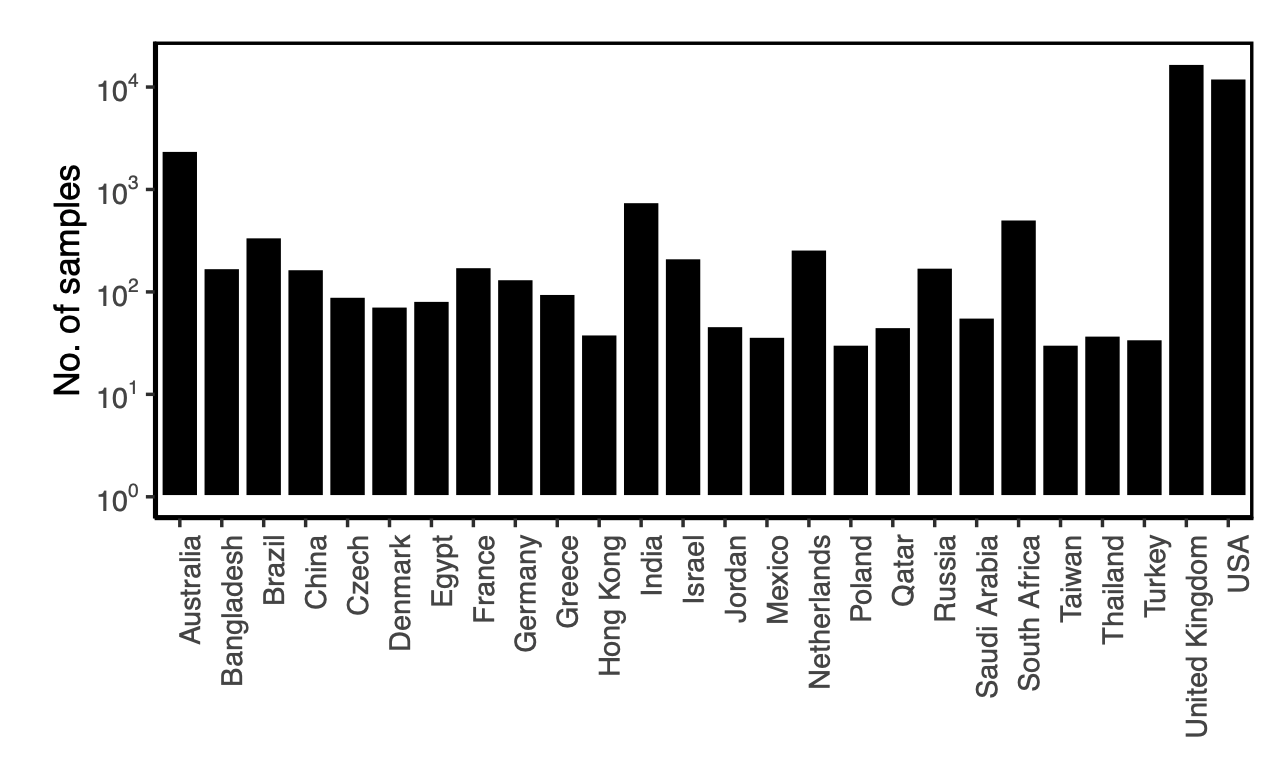


Figure S20


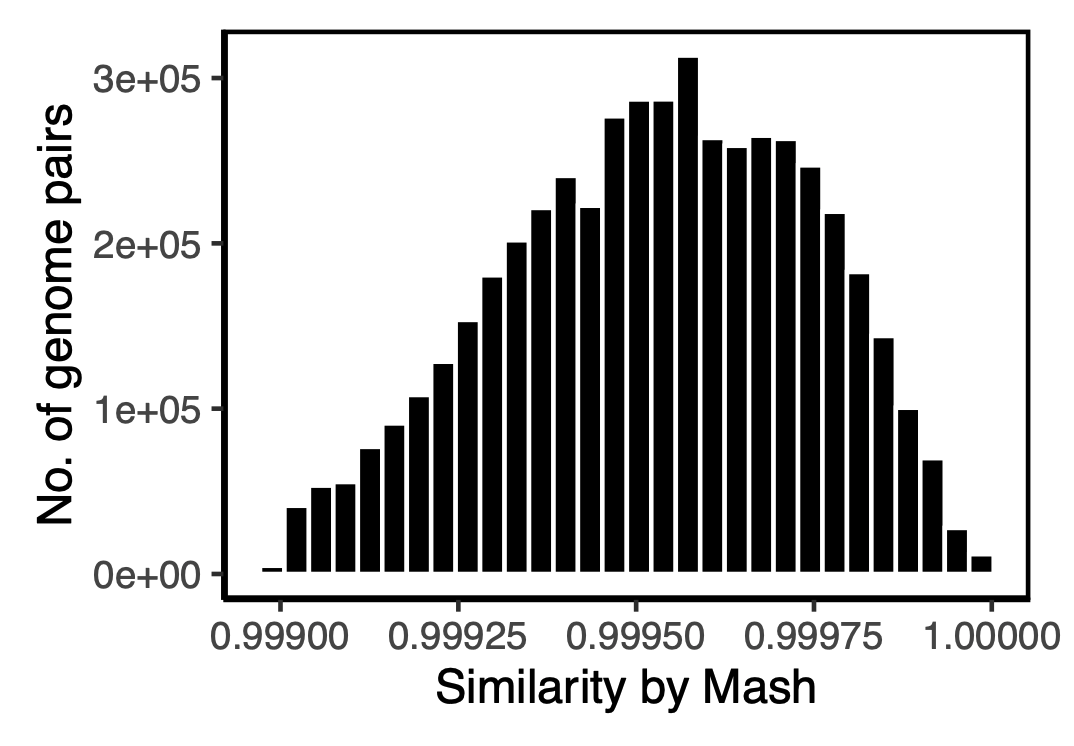


Figure S21


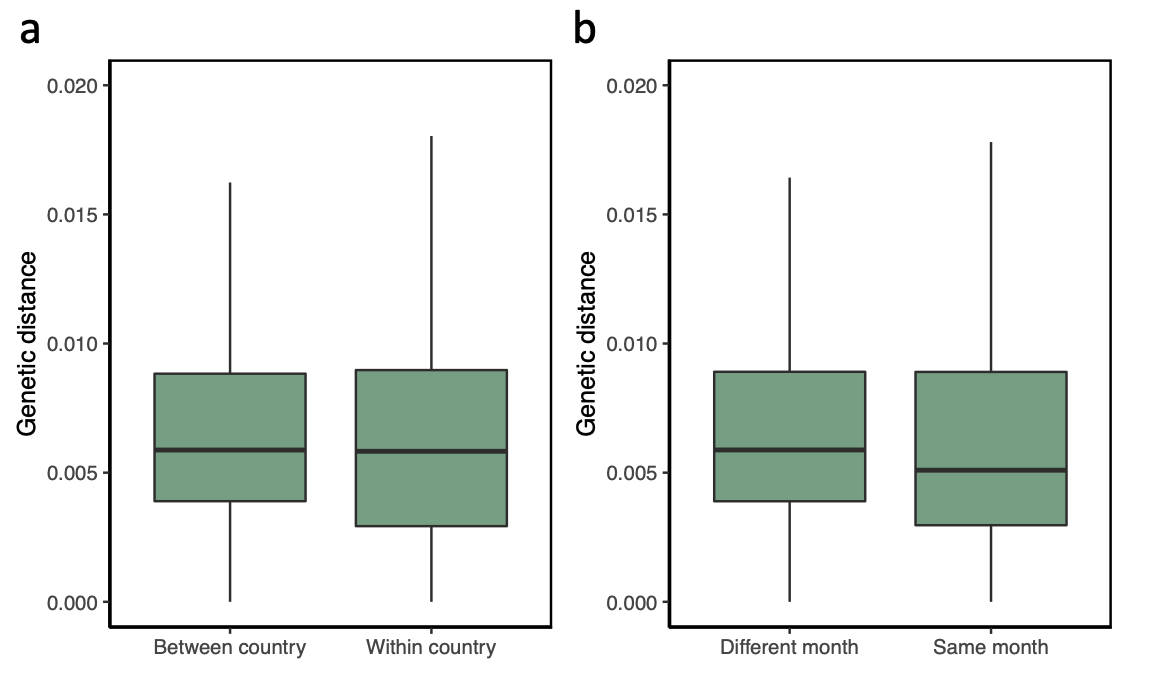


Figure S22


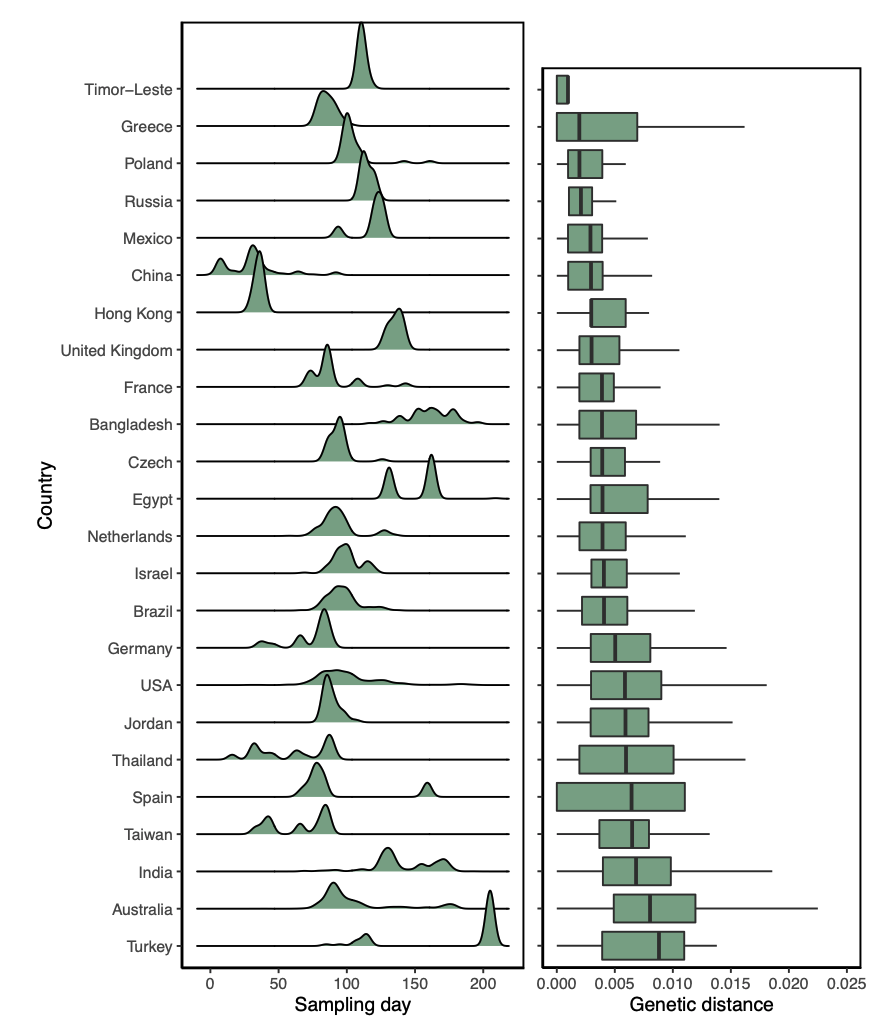

Supplement: Supplementary file 1 — Additional file 1. Supplementary Figures S1 to S22. [file 13059_2023_3030_MOESM1_ESM.docx]
