# Supplementary material for: Maast: genotyping thousands of microbial strains efficiently
Source: Genome Biol. 2023 Aug 10;24:186. doi: 10.1186/s13059-023-03030-8 (PMC10416524; doi:10.1186/s13059-023-03030-8)
Supplement: Supplementary file 11 — Additional file 11. Review history. [file 13059_2023_3030_MOESM11_ESM.docx]

Review History

**First round of review**

**Reviewer 1**

**Were you able to assess all statistics in the manuscript, including the appropriateness of statistical tests used?** Yes, and I have assessed the statistics in my report.

**Were you able to directly test the methods?** No.

**Comments to author:**

Shi et al. present Maast, a new approach for scalable genotyping of SNPs in microbial genomes, applicable to panels that comprise thousands of microbial genomes.

Maast implements a two-stage process. First, SNPs are identified based on a set of completely resolved microbial genome assemblies. Here, Maast uses the well-established MUMmer alignment algorithm for producing genome-to-genome alignments which SNPs are discovered from, but first employs a custom approach to reduce the input set of genomes to a representative set of "tag" genomes. Second, the identified SNP are genotyped in additional genomes (i.e. beyond the selected tag genomes), again employing the MUMmer-based alignment approach for completely resolved genomes or a k-mer-based SNP genotyping approach for genomes for which only short-read data are available.

In general I am very positive about this work and strongly support its publication. Maast is interesting from a methodological point of view, uses a clever implementation, and can contribute to comparing and systematizing large collections of microbial genomes, addressing an important need of the microbial genomics research community.

There are, however, some important points that should be addressed prior to publication.

- One intrinsic limitation of the Maast algorithm is that SNP discovery is carried out against a single reference genome, selected by the DynaCC algorithm. It is clear that this approach will fail to identify other potentially detectable variants from genomic regions not present in the selected genome. The approach chosen by Maast therefore sits somewhere between a core-genome-based method (which can detect only variants present in genomic regions that are present in almost *all* input genomes) and a full pangenomic method. First, this limitation should be made more explicit throughout the manuscript; second, it would be important to measure the extent of inter-genome variation actually lost due to the chosen approach in the various chosen experiments (see below for some suggestions); third, it would be important to at least try to include a "real" pangenomic method (e.g. potentially Cortex or the more recent developments coming out of Zam Iqbal's lab?) in the benchmarking experiments.

Metrics to measure the extent of inter-genome variability not captured by Maast could include a) the total number of variable positions identified in pairwise genome-to-genome alignments, stratified by whether they are present in the Maast SNP genotype reference panel or not; b) the average proportion of variant positions that distinguish between two randomly selected genomes present in the Maast SNP genotype reference panel. It would be important to report these statistics not only on the subset of genomes selected by DynaCC, but on the complete set of input genomes. To stratify individual positions according to a major allele frequency (MAF) criterion relative to a total set of input genome sequences S, one could, for a variable position with alleles x and y and positions p1 and p2 in genomes g1 and g2, independently check the proportion of genome sequences from S supporting x or y in g1 and g2 in genome-to-genome pairwise alignments.

- The authors claim, first, that many publicly available datasets exhibit a high level of "genome redundancy", biasing allele frequency estimates; and, second, that the allele frequencies in the subset of genomes selected using the DynaCC approach are somehow less biased. One example for this is the following sentence in the Discussion: "In fact, using these tag genomes improved our ability to identify SNPs at a given MAF and prevalence in the wider population by reducing bias due to uneven sampling of sequenced genomes".

While the first claim is highly plausible, the second claim seems to be only weakly substantiated. This point is also important because major allele frequency (MAF) is used for deciding which SNPs end up in the Maast SNP genotyping panel, and this set goes into the validation against e.g. Snippy.

The authors should make more explicit what they mean by "frequency bias"; how one would determine the "true" frequency of a variant; and provide evidence that allele frequencies in the DynaCC-selected set of genomes are actually closer to "true" allele frequencies than allele frequencies in the corresponding input datasets (i.e., pre-DynaCC).

This point could be supported by simulations, e.g. simulating genomes along a phylogenetic tree followed by biased sampling of particular branches of the tree; ideally, however, this point could also be shown with empirical data.

If this is not possible, it would be important to extend the validation experiments so that validation is also carried out based on the set of variants with 1% MAF in the original set of input genomes, and not limit the set of validated alleles to these meeting the 1% criterion only after DynaCC.

On a somewhat related note, in certain scenarios (e.g. genomic surveillance of hospital pathogens), capturing the full worldwide diversity of a species may be less important than the ability to differentiate between closely related strains, even if that entails using a "biased" frequency for allele selection.

- In multiple instances, the authors make claims along the lines of "Maast implements a novel algorithm to dynamically identify a minimum set of ... genomes ... that contains the maximum number of SNPs above a user-specified allele frequency". The objective function of the DynaCC algorithm, however, operates based on Mash distances, which correlate with ANIs/SNPs between genomes, but also capture other factors like structural variation between genomes. Even if this were not the case, however, the proposed algorithm would likely still be an approximation. It is therefore important to make clear that the presented algorithm is a heuristic which does not provably select the smallest set of genomes (or prove otherwise) .

- Validation experiments:

First, when processing real and simulated paired-end lllumina data, the authors apparently chose to only use the first read of each read pair; this is not a good choice because insert size information implicit in the read pairing information is used during mapping, in particular in repetitive regions; this could negatively affect e.g. Snippy performance. All experiments should be based on using paired-end data.

Second, for simulations, the authors use a coverage of 15X; this does not seem to be very realistic. In many settings, pathogen genomes are sequenced to about 100X. It would be important to either increase simulated coverage across the board, or alternatively carry out simulation at different coverage levels (in particular for the comparison against Snippy).

Third, the authors seem to benchmark Snippy on the reference genome chosen by DynaCC? I do not think this is necessarily how one would use Snippy - I assume one would probably just use the standard reference genome from RefSeq.

This latter point is also potentially relevant because it is not clear to me whether DynaCC may in general exhibit a bias for shorter genomes, i.e. genomes which potentially contain a smaller proportion of "accessory" genome sequence. If this is the case, then calling SNPs against the DynaCC-selected genome may result in a smaller number of discoverable variants compared to an approach that calls against the reference genome (it would also be great if the authors could comment on whether such an effect in DynaCC exists).

- Description of the method:

First, a slightly more formal description of how SNP positions are actually defined would be good. In the paper, the authors write: "Maast concatenates all qualified alignments and calls SNPs as sites with the following characteristics: 1) two or more nucleotides, ..". "Concatenation" here refers to a vertical concatenation? What happens in the case of individual alignments exhibiting INDELs around the putative SNP site? And does the algorithm only support bi-allelic SNPs (which I would infer e.g. from the description of the output format), or also SNPs with more than two alleles ("two or more nucleotides")?

Second, the authors do not describe the actual genotyping process, i.e. the process of going from k-mer-counts to a called genotype! What happens when both alleles receive some support? What happens when not all k-mers associated with an allele are found?

Third, regarding the k-mer-based genotyper: What happens when there SNPs within distance < k? Also, the paper says: "Maast extract sck-mers with the alternative allele ... selecting the most frequent 31-mer at each position". Which frequency specifically does this statement ("most frequent") refer to? Last, but not least, what happens in case of repeats? Is there a check that repetitive k-mers are not selected?

It would also be interesting to stratify the validation experiments by whether a variant is in a repetitive region or not - within these regions, one may expect Snippy to perform better, as the additional insert size information contained in paired-end reads should lead to improved mapping accuracy (as compared to relying on just k bases for establishing homology).

Fourth, accuracy metrics: "Sensitivity" is defined as "TP/(TP+FN) or the ratio of TP sites to all Maast sites". These two definitions do not seem equivalent to me - "all Maast sites" would also include FP, wouldn't it? Also, I would recommend renaming (1 - FDR) to PPV, as "discovery" may be understood in terms of "SNP allele discovery".

Fifth, the authors give an algorithm for counting how often the selected sck-mers appear in a set of input reads. I do not doubt the performance or accuracy of the algorithm, but a little bit more detail and intuition would be helpful. Could the authors define "colex order" and give an example? What are the advantages of this algorithm when compared to, say, a naive hashing-based algorithm?

**Reviewer 2**

**Were you able to assess all statistics in the manuscript, including the appropriateness of statistical tests used?** There are no statistics in the manuscript.

**Were you able to directly test the methods?** Yes.

**Comments to author:**

The authors describe and present Maast, an efficient and accurate microbial SNP genotyping software tool. Overall, the idea is innovative, the results are impressive, and the software tool quite promising. I commend the authors on the exhaustive analyses and experiments performed.

Maast fills a gap in current software for large-scale intraspecies microbial genome alignment. With few other methods in the last decade, there is room for improvement with respect to efficiency, accuracy, and usability. The workflow of Maast relies on popular and widely accepted tools for genomic distance measurements, pairwise genome alignment, and phylogeny reconstruction from core SNPs. Additionally, Maast allows some flexibility in trading off resolution for computational time by letting users choose the distance ranges for clusters. The results provided are very promising in terms of both accuracy and computational requirements. It is clear that Maast represents an important and timely contribution to the field.

Major comments

1. One of the major novelties of Maast is the clustering algorithm DynaCC, though it lacks some detail and additional motivation.

1a. For example, there is no mention of other non-graph based but sequence clustering algorithms like UCLUST, CD-HIT, MMSeqs2, etc, nor mention of any distance-based clustering algorithms. Without comparison or reason why DynaCC should outperform them, the novelty of the manuscript is unmotivated as it is unclear whether DynaCC even outperforms alternative methods.

2. Once clusters are identified, the reasoning behind the algorithms for selecting the representative (tag) and reference (centroid) genomes are lacking. In the case of tag genomes, the node with the most edges within the distance cutoff are selected as the tag genome for that component. It is not abundantly clear why the authors don't use one of the more studied and robust edge-centrality methods.

2a. Additionally, selecting the node with the most edges below some distance d seemingly discards the distance metric in favor of a binary presence/absence feature. Given that all pairwise distances have already been calculated, an approach which utilizes all pairwise distances seems like it would select a better representative at a marginal computational cost. Please clarify.

2b. In the case of the reference genome, it is not clear why the reference genome is picked as the tag genome w/ the lowest L-1 distance to all other tag genomes. E.g. why not instead the one that minimizes L-2 distance, or L-Inf distance? As the choice of reference has often been shown to influence alignment results, it is important to highlight robustness in reference selection via empirical results and/or theoretical reasoning.

3. This wasn't clear from the manuscript; was Maast benchmarked with or without the phylogeny computation step? Given that Parsnp seems to default to using Raxml for phylogeny, is it appropriate for the Maast alignment step to be compared to parsnp alignment+phylogeny? Even in the case where Maast does run phylogeny w/ fasttree, it seems like Parsnp should be run with -use-fasttree as well as opposed to default parameters.

4. I leave it to the authors discretion, but I strongly feel it would be helpful to see a comparison to multiple genome alignment tools capable of producing SNP genotyping info, such as Mugsy or Mauve. While runtimes for these tools can be prohibitive for large datasets, on smaller datasets it might help to shed light on pros and cons of Maast vs previous approaches based on multiple genome alignment.

Minor comments

5. It should be noted that Maast does not produce a multiple sequence alignment, and as far as I can tell, excludes insertion and deletion information.

6. When running on a set of 100 SARS-CoV-2 genomes, with the following command maast end_to_end --in-dir ./genomes-100/ --out-dir maast-out-100

the software crashed with the following error:

" cur_aln.ncols = len(cur_aln.seqs[0].seq)"

" IndexError: list index out of range"

7. Strongly encourage the authors to make the Maast software available on bioconda; it seems to be only available on their own conda channel

8. Maast should not default to using all available cores

9. It would be very informative and helpful to see a runtime breakdown of the pipeline per step

10. Axis labels and font size on figures 2-5 is quite small and difficult to read.

**Authors Response**

**Point-by-point responses to the reviewers’ comments:**

We are pleased that the reviewers are overall enthusiastic about our manuscript and the Maast software tool. To address their very helpful feedback, we provide below a point-by-point response detailing the changes we made to the manuscript and the new analyses that we performed, with our text in purple font. Changes in the manuscript documents are also highlighted by using purple font.

**Reviewer reports:

Reviewer #1:** Shi et al. present Maast, a new approach for scalable genotyping of SNPs in microbial genomes, applicable to panels that comprise thousands of microbial genomes.

Maast implements a two-stage process. First, SNPs are identified based on a set of completely resolved microbial genome assemblies. Here, Maast uses the well-established MUMmer alignment algorithm for producing genome-to-genome alignments which SNPs are discovered from, but first employs a custom approach to reduce the input set of genomes to a representative set of "tag" genomes. Second, the identified SNP are genotyped in additional genomes (i.e. beyond the selected tag genomes), again employing the MUMmer-based alignment approach for completely resolved genomes or a k-mer-based SNP genotyping approach for genomes for which only short-read data are available.

In general I am very positive about this work and strongly support its publication. Maast is interesting from a methodological point of view, uses a clever implementation, and can contribute to comparing and systematizing large collections of microbial genomes, addressing an important need of the microbial genomics research community.

Thanks, we are encouraged by the reviewer’s positive comments!

There are, however, some important points that should be addressed prior to publication.

- One intrinsic limitation of the Maast algorithm is that SNP discovery is carried out against a single reference genome, selected by the DynaCC algorithm. It is clear that this approach will fail to identify other potentially detectable variants from genomic regions not present in the selected genome. The approach chosen by Maast therefore sits somewhere between a core-genome-based method (which can detect only variants present in genomic regions that are present in almost *all* input genomes) and a full pangenomic method. First, this limitation should be made more explicit throughout the manuscript; second, it would be important to measure the extent of inter-genome variation actually lost due to the chosen approach in the various chosen experiments (see below for some suggestions); third, it would be important to at least try to include a "real" pangenomic method (e.g. potentially Cortex or the more recent developments coming out of Zam Iqbal's lab?) in the benchmarking experiments.

We thank the reviewer for their constructive feedback. We agree with them on this limitation of Maast, which is indeed a common limitation shared by methods that call variants using a single reference genome. Any method in this category will naturally miss out genetic variants that do not occur in the selected reference genome. This makes them a good fit for applications where capturing the majority of common variants is sufficient (e.g., building large phylogenetic trees, tracking pathogens and other strains that are not extremely closely related) and a less good fit for applications which require an exhaustive list of all variants or which focus on rare variants. Single reference dependent methods, like Maast, remain important and popular in practice because they tend to be computationally efficient and accurate.

To address the first point, we added comments to the Results and Discussion (lines 134-137, 296-305) noting that Maast cannot genotype sites that are absent from the reference genome. We agree with the reviewer that this is important.

The second point regarding assessing the extent of diversity lost with different methods is closely related with the comment immediately below, and we address it there.

For the last point, we agree that it would be fantastic to include a pangenome variant calling method in our comparisons. To execute on this, we made multiple attempts to run both cortex [1] and a more recent method called mccortex [2] from Zam Iqbal’s lab. Unfortunately, we could not successfully call pangenome variants on our benchmarking dataset of Illumina reads from 45 *B. uniformis* isolates simulated at 15x coverage. The error messages suggest that these methods would require a huge amount of memory (e.g., “Fatal error: Not enough memory for requested graph: require at least 7.8TB”, “Error: Full Hash table”). Upon running them on a smaller dataset of 5 only isolates, we again encountered memory issues (e.g., “Dear user - you have not allocated enough memory to contain your sequence data”, “Require at least 160GB memory for paths”). Our conclusion is that the tools for detecting pangenome variants are a promising direction, because they are designed to detect a higher number of variants, especially rare variants, compared to reference genome methods. But pangenome methods are much slower and require a lot of RAM, and hence they do not scale well in their current implementations to the increasingly large data sets being analyzed in the metagenomics field. We added new text (lines 300-305) about the variant discovery potential and the computational limitations of pangenome methods to the Discussion section.

Metrics to measure the extent of inter-genome variability not captured by Maast could include a) the total number of variable positions identified in pairwise genome-to-genome alignments, stratified by whether they are present in the Maast SNP genotype reference panel or not; b) the average proportion of variant positions that distinguish between two randomly selected genomes present in the Maast SNP genotype reference panel. It would be important to report these statistics not only on the subset of genomes selected by DynaCC, but on the complete set of input genomes. To stratify individual positions according to a major allele frequency (MAF) criterion relative to a total set of input genome sequences S, one could, for a variable position with alleles x and y and positions p1 and p2 in genomes g1 and g2, independently check the proportion of genome sequences from S supporting x or y in g1 and g2 in genome-to-genome pairwise alignments.

We thank the reviewer for these great suggestions! To address this comment, we decided to further dig into the eight example species representing two distinct groups: one with a high level of redundancy in their conspecific genomes and the other with low redundancy. We measured the two metrics suggested by the reviewer using genome-to-genome alignments across these species to tally the total number of variable sites between any pair of genomes and then stratifying these by whether or not they are in (i) the Maast SNP panel based on tag genomes (DynaCC, MAF threshold = 1%) or (ii) the Maast SNP panel based on all genomes (no DynaCC, MAF threshold = 1%). We also computed the MAF of all variable positions across all genomes.

The results for metric (a) are as follows. We observed that the Maast SNP panel (MAF threshold = 1%) captures <50% of the total number of variable positions present in pairwise alignments regardless of whether we run Maast with tag genomes or all genomes. The variable positions in pairwise alignments that are absent from both SNP panels (tag and all) have very low MAF across the set of input genomes (MAF < 1% across all genomes by definition, but in fact much less than 1% in most cases). Maast does not attempt to genotype rare variants, but we agree with the reviewer that it is useful for readers and users to know that more than half of all variable positions are rare and hence are not genotyped by Maast. The reviewer is perhaps more interested in differences between the SNP panels based on all genomes versus tag genomes. Consistent with our previously reported results, we found that more variable positions are captured using tag genomes and this is driven largely by high redundancy species. For two low redundancy species, the SNP panel based on all genomes captures somewhat more variable sites. Most of the tag-only SNPs have MAF in the full set of genomes that is between 0.1% and 1% (Figure 2d). We added these new findings to the Results section (lines 120-130) and Supplementary Figure S7.

**Figure S7. The number of SNPs identified in the same eight species as Figure 2c.** The total number of SNPs identified in pairwise genome-to-genome alignments and the number of these that are found in the SNP panel that was called with tag genomes (green) or with all genomes (grey). The color of the x axis label indicates the level of redundancy in the conspecific genomes of the species: high (red) versus low (blue).

The results for metric (b) are as follows. Overall, using tag genomes resulted in a somewhat lower proportion of variable sites being included in the SNP panel. However, when focusing on the most divergent pairs of genomes (i.e., the ones generating the most SNPs), we found that using tag genomes recovered a higher proportion of variants from pairwise alignments for species with high genome redundancy and a similar or somewhat lower proportion for species with low genome redundancy. These new results highlight that DynaCC is most useful from the perspective of SNP discovery when redundancy is present in the genome collection, and that this comes at the expense of missing a very small proportion of the SNPs present in pairwise alignments that could be discovered using all genomes. We added these new findings to the Results section (lines 120-130) and Supplementary Figure S8.

**Figure S8. The median proportion of SNPs from a genome pair that are present in a reference SNP panel.** Each genome pair consists of a reference genome and a conspecific genome. The reference SNP panel is generated with a similar SNP calling workflow using tag genomes (green) or all input genomes (gray). The left panel included all genome pairs and the right panel shows the top 20 most divergent pairs, which are the pairs generating the most SNPs. The color of the y axis label indicates the level of redundancy in the conspecific genomes of the species: high redundancy (red) or low redundancy (blue).

In sum, these metrics suggest that DynaCC and using tag genomes can mitigate the loss of SNPs due to low MAF estimates in species with high genome redundancy, while it may slightly reduce SNP discovery compared to using all genomes for species with low genome redundancy. This is an important consideration that we now explain to users. Combined with our other results, we conclude that DynaCC is undoubtedly helpful from a computational point of view (species with many genomes) and for mitigating bias from oversampled subpopulations (species with redundant genomes), while the SNPs that it misses tend to be rare (present in some pairwise alignments but not prevalent across genomes). Since increasingly many species have high redundancy and/or large numbers of genomes, Maast uses DynaCC and tag genomes by default. But users have the option to run Maast with all genomes if they have sufficient computing resources and are confident that they do not have high genome redundancy. We added this guidance to the Results and Methods sections (lines 120-130, 385-387). These new findings also underscore the fact that rare SNPs and SNPs in the accessory genome will not be genotyped by Maast regardless of whether one uses all genomes or tag genomes to design the SNP panel. We now cover Maast’s limitation regarding genotyping rare SNPs explicitly in the Discussion section (lines 296-305).

- The authors claim, first, that many publicly available datasets exhibit a high level of "genome redundancy", biasing allele frequency estimates; and, second, that the allele frequencies in the subset of genomes selected using the DynaCC approach are somehow less biased. One example for this is the following sentence in the Discussion: "In fact, using these tag genomes improved our ability to identify SNPs at a given MAF and prevalence in the wider population by reducing bias due to uneven sampling of sequenced genomes".
While the first claim is highly plausible, the second claim seems to be only weakly substantiated. This point is also important because major allele frequency (MAF) is used for deciding which SNPs end up in the Maast SNP genotyping panel, and this set goes into the validation against e.g. Snippy.
The authors should make more explicit what they mean by "frequency bias"; how one would determine the "true" frequency of a variant; and provide evidence that allele frequencies in the DynaCC-selected set of genomes are actually closer to "true" allele frequencies than allele frequencies in the corresponding input datasets (i.e., pre-DynaCC).
This point could be supported by simulations, e.g. simulating genomes along a phylogenetic tree followed by biased sampling of particular branches of the tree; ideally, however, this point could also be shown with empirical data.
If this is not possible, it would be important to extend the validation experiments so that validation is also carried out based on the set of variants with 1% MAF in the original set of input genomes, and not limit the set of validated alleles to these meeting the 1% criterion only after DynaCC.

We appreciate the reviewer’s thoughtful comments about DynaCC. By “frequency bias”, we mean under- or over-estimation of the frequency of an allele at any given genomic position due to the biased sampling of the true population, or more specifically, the over-representation of a subpopulation (e.g., clonal genomes from one single host or hosts with recent transmission). In many situations, determining the “true” frequency of a variant is unfortunately difficult, if not impossible, because the true population is inaccessible. But it is clear, nonetheless, that over-sampling a subpopulation will bias the estimate of MAF in the direction of whichever allele is present in the subpopulation. Consider an extreme example of a SNP with MAF=50% in a population. Suppose we sample 1,000 conspecific genomes, among which 991 genomes are clonal and have the identical sequence in this locus, while the remaining 9 genomes are from distinct subpopulations (say, 4 have the same allele at this site as the 991 clonal genomes and 5 have the other allele). When calling SNPs with a threshold of MAF>1% on this example genome set, this SNP will have an estimated MAF=0.5% even though the true MAF in the population is 50%. In real cases, although over-sampling may be less severe, presence of a large number of clonal genomes can result in biased estimation of the true population MAF in a similar way. In the previous submission, we demonstrated this point by showing that using tag genomes selected by DynaCC generally results in a higher number of variants compared to using a full set of genomes. We also showed that when this difference is large the species tends to have a large cluster of highly similar genomes (Figure 2d,f), which indirectly supported our hypothesis. The reviewer’s comments indicate that they generally agree with this first assertion.

Regarding the second claim, the reviewer is correct that we did not previously provide strong evidence. To address this, we performed both experiments suggested by the reviewer and added the results to the manuscript. In the first experiment, we compared what proportion of true variants were recovered by using tag genomes versus the full set of all genomes. We adopted the reviewer’s suggestion to construct a phylogenetic tree based on a multiple sequence alignment of the observed genomes, and we then defined true variants as those called with genomes sampled from distinct clades in the tree. We focused on four species with a relatively high proportion of discordant SNPs between tag genomes and all genomes. We found that using tag genomes results in more true variants consistently compared to using all genomes (Figure 2e), while the rate of false positive variants is higher in *Faecalibacterium prausnitzii_K* and *Phascolarctobacterium faecium* and lower in *Alistipes shahii* and *Akkermansia muciniphila_B*.

In the second experiment, we compared Maast and Snippy genotyping sensitivity using the SNPs (MAF >1%) called with the original set of all genomes as ground truth. We found that the sensitivity of Maast was decreased compared to the original benchmarking results in our initial submission due to differences between the Maast SNP panel and ground truth SNPs. However, Maast’s sensitivity remained higher than that of Snippy (median=87.6% versus 54.7% for simulated reads and 87.6% versus 61.9% for downloaded reads). The sensitivity of Snippy was essentially unchanged compared to the sensitivity before the switch (54.3% and 61.7% for simulated and downloaded reads). These results suggest that Maast provides improved sensitivity over Snippy regardless of the ground truth set of SNPs, while affirming that the Maast SNP panel misses some SNPs present in all genomes. As expected, Maast’s PPV is much lower using the original set of all genomes as ground truth compared to using Maast’s SNP panel as ground truth, because many SNPs genotyped by Maast are not in the ground truth and thus are counted as false positives. It is worth noting that those SNPs may not be false positives from the perspective of the (unknown) true population of genomes, for example, if they are absent from the ground truth set due to over-sampling of a subpopulation. Finally, PPV is essentially unchanged when the set of true SNPs is defined as the union of SNPs from the Maast SNP panel and the SNPs identified in all genomes, suggesting that indeed very few new false positives were introduced by switching the ground truth from the Maast SNP panel based on tag genomes to the one based on all genomes. We added these findings in Supplementary Figure S11.

Altogether, our new results using three sets of ground truth SNPs (pairwise genome comparisons, even phylogenetic sampling, and all genomes) consistently show that the main limitation of Maast is that it cannot genotype rare SNPs that are not in its tag SNP panel. Otherwise, Maast is very accurate. Furthermore, these results provide new support for our conclusion that Maast can effectively overcome MAF estimation biases due to redundant genomes.

**Figure 2e.** Total number of ground truth SNPs based on even phylogenetic sampling that controls bias from over-sampled subpopulations (Truth), number of true SNPs found in the SNP panel called with Maast tag genomes (Tag), and number of true SNPs found in the SNP panel called using all input genomes (All).

**Figure S10.** **Comparison of Maast and Snippy genotyping accuracy at non-reference alleles of SNPs based on simulated whole-genome sequencing.** Both Maast and Snippy were run with default settings. (a and b) Positive predictive value (PPV) comparison, where true positives are genotype calls that match the genome. (c and d) Sensitivity is the probability of detecting genotypes present in the SNP panel generated with Maast (tag genomes, left), all input genomes (middle), or the union of these two panels (right). The analysis is based on short reads (a and c) simulated at 15x coverage from isolate genomes with sequencing error and (b and d) downloaded from isolate whole-genome sequencing projects. Both Maast and Snippy were run with default settings. Left panels are identical to the corresponding panels in Figure 3c-f and are included here for side-by-side comparison.

On a somewhat related note, in certain scenarios (e.g. genomic surveillance of hospital pathogens), capturing the full worldwide diversity of a species may be less important than the ability to differentiate between closely related strains, even if that entails using a "biased" frequency for allele selection.

We agree and have added this point to the Discussion section (lines 299-300).

- In multiple instances, the authors make claims along the lines of "Maast implements a novel algorithm to dynamically identify a minimum set of ... genomes ... that contains the maximum number of SNPs above a user-specified allele frequency". The objective function of the DynaCC algorithm, however, operates based on Mash distances, which correlate with ANIs/SNPs between genomes, but also capture other factors like structural variation between genomes. Even if this were not the case, however, the proposed algorithm would likely still be an approximation. It is therefore important to make clear that the presented algorithm is a heuristic which does not provably select the smallest set of genomes (or prove otherwise) .

This comment makes a good point. To address it, we edited the abstract (lines 18-19), Results section (lines 103-106), and Methods section (lines 369-371) to clarify that Maast is a heuristic designed for rapid sampling of genomes from distinct subpopulations. We also added a comment in the Results section to note that the tag genomes have not been proved to be the smallest set of genomes (line 106).

- Validation experiments:

First, when processing real and simulated paired-end lllumina data, the authors apparently chose to only use the first read of each read pair; this is not a good choice because insert size information implicit in the read pairing information is used during mapping, in particular in repetitive regions; this could negatively affect e.g. Snippy performance. All experiments should be based on using paired-end data.

Thanks for pointing this out. Reflecting on the reviewer’s comment, we agree that only using forward reads could negatively affect Snippy’s performance. Since Maast does not use read pairing information (it processes a pair of reads the same as two single reads), its accuracy is not likely to be affected by whether input reads are paired or unpaired. To address the reviewer’s suggestion, we repeated the downloaded reads benchmark but this time ran Snippy using paired-end reads (forward and reverse reads were both supplied and aligned as pairs). We ran Maast on the same paired-end reads as a single merged file by simply concatenating the forward and reverse read files. We note that compared to running with only forward reads these analyses using paired-end reads have effectively double the sequencing coverage, so we also ran Snippy with the full set of forward and reverse reads but without processing them as paired-end reads in order to disentangle the effect of coverage from the effect of paired-end alignment. Consistent with our previous findings, the PPV of Maast was slightly higher (median=99.7%) compared to Snippy (median=98.2%), while the sensitivity of Maast is notably higher. Compared to genotyping with forward reads only, the sensitivity of Snippy improved slightly with paired-end reads. However, the PPV of both Maast and Snippy decreased a small amount, likely due to more false positives occurring by chance as the sequencing coverage gets higher. These findings have been added to the manuscript (lines 209-210, Figure S12). Similar analyses using simulated reads were also performed and are presented immediately below.

**Figure S12. Comparison of Maast and Snippy genotyping accuracy at non-reference alleles of SNPs based on downloaded whole-genome sequencing.** SNPs in the Maast SNP panel were called in short reads downloaded from isolate whole-genome sequencing projects based on forward reads only (left) and paired-end reads (right). Both Maast and Snippy were run with default settings. (upper) Positive predictive value (PPV) comparison, where true positive discoveries are genotype calls that match the genome. (lower) Sensitivity from simulations with the Maast SNP panel as reference. Sensitivity is the probability of detecting genotypes present in the genome. Left panels are the same as the corresponding panels in Figure 3e and f and are included here for side-by-side comparison.

Second, for simulations, the authors use a coverage of 15X; this does not seem to be very realistic. In many settings, pathogen genomes are sequenced to about 100X. It would be important to either increase simulated coverage across the board, or alternatively carry out simulation at different coverage levels (in particular for the comparison against Snippy).

Great suggestion! We would like to clarify that we chose 15X since we did not expect sequencing coverage to be a main determinant of performance. We now realize that 15X coverage may have a negative impact on Snippy, specifically resulting in lower sensitivity compared to higher coverage, since the tool applies a coverage filter by default to exclude the regions of reference genomes poorly covered by mapped reads for the purpose of accuracy. As suggested, we now simulated reads for each genome of our example species at 100X coverage and compared genotyping accuracy between Maast and Snippy with the simulated reads as in the previous analysis. The simulated reads were processed as both paired-end reads and single-end reads. As sequencing coverage increased from 15X to 100X, the PPV decreased slightly for both Maast (from 99.7% to 99.3%) and Snippy (from 99.4% to 98.4%). As postulated by the reviewer, Snippy’s sensitivity is somewhat higher at 100X coverage (64.0%) compared to that at 15X coverage (54.3%), while it remained largely unchanged for Maast. No difference was found for PPV or sensitivity between pair-end versus single-end reads. These findings support our previous conclusions that Maast has somewhat higher PPV (lower FDR) and much higher sensitivity compared to Snippy, including in the scenario of high sequencing coverage. We added these new benchmarking results to the Results section (lines 209-210) and Figure S13.

**Figure S13. Comparison of Maast and Snippy genotyping accuracy at non-reference alleles of SNPs at different simulated sequencing coverages.** SNPs in the Maast SNP panel were genotyped based on short reads simulated from isolate genomes with sequencing error at 15x (left) and 100x (middle and right) coverage. Short reads simulated at 100x coverage were processed as both single (middle) and paired end (right) reads. Both Maast and Snippy were run with default settings. (upper) Positive predictive value (PPV) comparison, where true positive discoveries are genotype calls that match the genome. (lower) Sensitivity from the simulations with the Maast SNP panel as reference. Sensitivity is the probability of detecting genotypes present in the genome. Left panels are the same as the corresponding panels in Figure 3c and d and are included in order to enable side-by-side comparisons.

Third, the authors seem to benchmark Snippy on the reference genome chosen by DynaCC? I do not think this is necessarily how one would use Snippy - I assume one would probably just use the standard reference genome from RefSeq. This latter point is also potentially relevant because it is not clear to me whether DynaCC may in general exhibit a bias for shorter genomes, i.e. genomes which potentially contain a smaller proportion of "accessory" genome sequence. If this is the case, then calling SNPs against the DynaCC-selected genome may result in a smaller number of discoverable variants compared to an approach that calls against the reference genome (it would also be great if the authors could comment on whether such an effect in DynaCC exists).

We apologize that it was not clear which genomes were used for benchmarking. We used the representative genomes from UHGG species, instead of the centroid genome identified by DynaCC, as the reference genomes for the benchmarking to control possible biases including those highlighted by the reviewer. Since the UHGG representative genomes were selected based on genome quality independently from the Maast workflow, our results should be very similar to what one would observe using RefSeq or other high-quality representative genomes. We edited the Methods section (lines 548-550) to clarify that UHGG representative genomes were used for the benchmarking experiments.

To comment here on possible biases of tag genomes, we agree DynaCC could favor shorter genomes in centroid genome picking for the exact reason pointed out by the reviewer. Since the reviewer brought up this interesting point, we wish to postulate that using tag genomes would not necessarily result in as much of a change as one might expect in the number of discoverable variants compared to a longer reference genome, because we have observed that common SNPs are less likely to be found in “accessory” genomic regions. Related to this point, in one of our recent studies [3], we showed for one example species that using the centroid genome increases the overall short read alignment rate compared to using the default species representative genome. We expect that there would be both pros and cons to using the tag genomes. Nonetheless, we stuck with UHGG representative genomes in the evaluations in order to avoid biases that would unfairly favor Maast.

Finally, we would like to note that in the main program of Maast, there is a flag “--skip-centroid” to turn off the default selection of the centroid genome as reference for SNP calling. If a reference genome is designated with the “--rep-fna” flag, the selection of the centroid genome will also be automatically turned off. Thus, users may supply their own reference genomes, such as RefSeq genomes.

- Description of the method:

First, a slightly more formal description of how SNP positions are actually defined would be good. In the paper, the authors write: "Maast concatenates all qualified alignments and calls SNPs as sites with the following characteristics: 1) two or more nucleotides, ..". "Concatenation" here refers to a vertical concatenation? What happens in the case of individual alignments exhibiting INDELs around the putative SNP site? And does the algorithm only support bi-allelic SNPs (which I would infer e.g. from the description of the output format), or also SNPs with more than two alleles ("two or more nucleotides")?

Thanks for these questions. We added more details in the Methods section to clarify the SNP calling process in Maast (see below). To answer the reviewer’s questions here:

*Two or more nucleotides, ..". "Concatenation" here refers to a vertical concatenation?*

“Concatenation” here refers to a horizontal concatenation. We edit the text (line 449) to clarify this.

*What happens in the case of individual alignments exhibiting INDELs around the putative SNP site?*

This is an important point. We extract as many k-mers as possible that cover a SNP site. We have considered three scenarios for INDELs near (within 30 bp of a SNP): no INDELs, INDELs at one side of a SNP site, and INDELs at both sides of a SNP site. When no INDELs are present, all 31 sck-mers will be extracted (SNP site can be at any base on a 31-mer). When INDELs only occur on one side of a SNP site, 1 to 30 sck-mers will be extracted, depending on the location of the nearest INDEL to the SNP site. In rare cases where two or more INDELs flank a SNP site, no sck-mers will be extracted. We added text with these details to the Methods section (lines 479-485).

*And does the algorithm only support bi-allelic SNPs (which I would infer e.g. from the description of the output format), or also SNPs with more than two alleles ("two or more nucleotides")?*

Correct - currently the algorithm only supports bi-allelic SNPs which are the vast majority of SNPs. We added this information to the Methods section (lines 516-517) and clarified in Results (lines 132-134) that we genotype common, bi-allelic SNPs only. In the Discussion section (lines 296-305), we now talk about pros and cons of this choice.

Second, the authors do not describe the actual genotyping process, i.e. the process of going from k-mer-counts to a called genotype! What happens when both alleles receive some support? What happens when not all k-mers associated with an allele are found?

Great point. We added clarification about the actual genotyping process with new text in the Methods section (lines 509-511). To briefly answer the specific questions posed here, if both alleles are detected, the counts of both alleles will be reported. The count of an allele will be increased by one as long as at least one k-mer is detected in the read.

Third, regarding the k-mer-based genotyper: What happens when there SNPs within distance < k? Also, the paper says: "Maast extract sck-mers with the alternative allele ... selecting the most frequent 31-mer at each position". Which frequency specifically does this statement ("most frequent") refer to? Last, but not least, what happens in case of repeats? Is there a check that repetitive k-mers are not selected? It would also be interesting to stratify the validation experiments by whether a variant is in a repetitive region or not - within these regions, one may expect Snippy to perform better, as the additional insert size information contained in paired-end reads should lead to improved mapping accuracy (as compared to relying on just k bases for establishing homology).

The reviewer points out that we need to provide more detail about how sck-mers are extracted, which is a good suggestion. Maast extracts sck-mers by sliding a fixed size window along the multiple sequence alignment. At any given position, the window allows extraction of n sck-mers, where n equals the number of rows in the multiple sequence alignment (i.e., number of aligned genomes). Next, Maast reduces these n sck-mers to m unique sck-mers (m$\leq$n) and selects the one from these m with the highest frequency across the n genomes. We added this key information to the Methods section (lines 474-477).

Great question about repetitive k-mers! Maast indeed was built with an important step designed to rule out all repetitive k-mers. Specifically, every candidate sck-mer is searched in all input genomes, not only tag genomes, and it is removed if it occurs anywhere else in any genome. We now thoroughly describe this step in the Methods section (lines 485-487) and refer to it in the Results section (lines 141-142).

Fourth, accuracy metrics: "Sensitivity" is defined as "TP/(TP+FN) or the ratio of TP sites to all Maast sites". These two definitions do not seem equivalent to me - "all Maast sites" would also include FP, wouldn't it? Also, I would recommend renaming (1 - FDR) to PPV, as "discovery" may be understood in terms of "SNP allele discovery".

We apologize for the confusion and agree that “all Maast sites” could have been interpreted to mean all positives (TP + FP). We intended it to mean all true SNPs that can be genotyped by Maast (TP+FN). We clarified the definition of sensitivity by using the text “TP/(TP+FN) or the ratio of TP sites to the total number of sites in the Maast SNP panel”. In other words, the world of possible TPs is any site that can be genotyped, and sensitivity is the proportion of these that are detected by Maast. This has been changed in the Methods section (lines 635-637). We also renamed (1 - FDR) to PPV as suggested. Sensitivity is similarly defined for the other ground truth SNP sets in the manuscript. Thanks for helping us provide clear definitions.

Fifth, the authors give an algorithm for counting how often the selected sck-mers appear in a set of input reads. I do not doubt the performance or accuracy of the algorithm, but a little bit more detail and intuition would be helpful. Could the authors define "colex order" and give an example? What are the advantages of this algorithm when compared to, say, a naive hashing-based algorithm?

Thanks for this fantastic suggestion. We realize now that we did not provide enough detail about colex ordering and the exact matching algorithm. Colex order is essentially a reflection of lexicographic order which reads a sequence from the right to the left (versus left to right). This enables efficient searching of string suffixes. We clarified the term “colex order” and provided an example. The matching algorithm takes advantage of the observation that most query k-mers have no chance to match sck-mers in the database. These can be identified by lack of a matching subsequence (similar to a Bloom filter). We implemented this strategy by applying a smaller and denser hash table than the table that would be used for a full hash table approach. This table is made up of suffix subsequences (i.e., l-mers with l<m). We then use the l-mers to screen query k-mers, and only those with an l-mer exact match move forward to full length k-mer exact matching. This two-step algorithm is faster and more RAM efficient than using a full hash table to identify exactly matching k-mers. We added this information to the Results section (lines 143-145) with additional new details in the Methods section as well (lines 491-498).

**Reviewer #2:** The authors describe and present Maast, an efficient and accurate microbial SNP genotyping software tool. Overall, the idea is innovative, the results are impressive, and the software tool quite promising. I commend the authors on the exhaustive analyses and experiments performed.

Maast fills a gap in current software for large-scale intraspecies microbial genome alignment. With few other methods in the last decade, there is room for improvement with respect to efficiency, accuracy, and usability. The workflow of Maast relies on popular and widely accepted tools for genomic distance measurements, pairwise genome alignment, and phylogeny reconstruction from core SNPs. Additionally, Maast allows some flexibility in trading off resolution for computational time by letting users choose the distance ranges for clusters. The results provided are very promising in terms of both accuracy and computational requirements. It is clear that Maast represents an important and timely contribution to the field.

We would like to thank the reviewer for positive assessment!

Major comments
1.    One of the major novelties of Maast is the clustering algorithm DynaCC, though it lacks some detail and additional motivation.

1a.    For example, there is no mention of other non-graph based but sequence clustering algorithms like UCLUST, CD-HIT, MMSeqs2, etc, nor mention of any distance-based clustering algorithms. Without comparison or reason why DynaCC should outperform them, the novelty of the manuscript is unmotivated as it is unclear whether DynaCC even outperforms alternative methods.

Thanks for this helpful comment! We would like to note two main differences between DynaCC and other clustering algorithms. First, DynaCC leverages a fast approximation of ANI, making it better suited for processing a large number of whole genome sequences. To explore this point, we ran DynaCC, CD-HIT[4], vsearch (UCLUST equivalent) [5] and MMSeqs2 [6] on 3,646 whole genomes of *Alistipes putredinis* (Species ID: 101302). All programs were run in an identical computing environment (AWS EC2 r5.16xlarge) for a maximum of 48h. DynaCC successfully divided these genomes into 110 clusters in ~3 minutes, while the same task took MMSeqs2 ~4h. Both CD-HIT and vsearch ultimately did not run through the task: the CD-HIT main program was terminated by signal 11 (reached 498GB RAM cap) and vsearch did not complete within 48h. Second, DynaCC is more efficient at dividing whole genome sequences into a fixed number of clusters. Other algorithms will usually take one single distance (or similarity) cutoff as input and use this same cutoff across all species to divide genome sequences into a smaller untargeted number of clusters. DynaCC automatically searches a large space of cutoffs around a target minimum number of clusters (i.e., tag genomes) and finds a different optimal cutoff per species to generate at least the targeted number of clusters. For example, DynaCC automatically divided genomes of *A. putredinis* (n=3,646) and *A. rectalis* (n=5,214), two example species in Figure S5, into ~100 clusters (110 clusters for *A. putredinis* and 106 for *A. rectalis*), while using a static genomic distance cutoff of 0.01 resulted in 46 genome clusters for *A. putredinis* and 2,650 clusters for *A. rectalis*. In contrast, MMSeqs2 by default generated 3,646 *A. putredinis* clusters and 5,214 *A. rectalis* clusters, barely collapsing any redundant genomes. Thus, DynaCC rapidly prunes a large set of genomes to a consistent target size across diverse and heterogeneous species.

We implemented a method with this behavior because generating approximately the same number of clusters across species (i.e., same order of magnitude number) is an important component of Maast’s computational efficiency in downstream SNP calling. We added this information to the Methods section (lines 406-428).

We would also like to clarify that DynaCC only accounts for one part of the novelty that Maast presents. The key innovations of Maast are its efficiency and novel workflow. The main workflow of Maast implements a catalog-first-genotype-later scheme. It was designed in order to enable genotyping to be scaled up for very large populations. Maast also synchronizes microbial genotyping for both genome assemblies and sequencing reads (isolates or metagenomes). We agree with the reviewer that it is important to note that the underlying algorithms in the Maast workflow leverage previously existing methods for whole genome alignment and k-mer exact matching. To our knowledge, the Maast workflow is a novel combination of these methods (i.e., no other tools implement a similar workflow), and other tools are not as efficient as Maast. We included text clarifying what is and is not novel about Maast in the updated Methods section (lines 337-342).

2.    Once clusters are identified, the reasoning behind the algorithms for selecting the representative (tag) and reference (centroid) genomes are lacking. In the case of tag genomes, the node with the most edges within the distance cutoff are selected as the tag genome for that component. It is not abundantly clear why the authors don't use one of the more studied and robust edge-centrality methods.

Thanks for the great suggestion. We chose to use the genome with the most edges (degree centrality after removing edges below the distance cutoff), because it is intuitive and computationally efficient. We agree that there are other well-studied and suitable methods to estimate the centrality of a node. We addressed this comment by implementing multiple popular methods for centrality calculation in addition to degree centrality in the newest version of Maast. These methods include eigenvector, closeness, information, betweenness and load centrality, which can be freely chosen by users using the flag “--tag-centrality”. We noted this change in the updated Methods section (lines 381-387).

2a.    Additionally, selecting the node with the most edges below some distance d seemingly discards the distance metric in favor of a binary presence/absence feature. Given that all pairwise distances have already been calculated, an approach which utilizes all pairwise distances seems like it would select a better representative at a marginal computational cost. Please clarify.

We thank the reviewer for this great suggestion. We binarized edges mainly for simplicity, and we agree that binarizing the distance throws away already computed distance information. We now added a flag “--edge-weighted” to the Maast program to enable the use of actual distance information for representative selection. This change is noted in the updated Methods section (lines 383).

2b.    In the case of the reference genome, it is not clear why the reference genome is picked as the tag genome w/ the lowest L-1 distance to all other tag genomes. E.g. why not instead the one that minimizes L-2 distance, or L-Inf distance? As the choice of reference has often been shown to influence alignment results, it is important to highlight robustness in reference selection via empirical results and/or theoretical reasoning.

This is a great suggestion. To address it, we added L-2 and L-Inf distance as user options for selecting reference genomes (along with the default L-1 distance). Users can select a specific distance metric using the flag “--centroid-distance”. We added this updated option to the Methods section (lines 434-436).

3.    This wasn't clear from the manuscript; was Maast benchmarked with or without the phylogeny computation step? Given that Parsnp seems to default to using Raxml for phylogeny, is it appropriate for the Maast alignment step to be compared to parsnp alignment+phylogeny? Even in the case where Maast does run phylogeny w/ fasttree, it seems like Parsnp should be run with -use-fasttree as well as opposed to default parameters.

Thanks for this comment. We agree with the reviewer that using Raxml versus Fasttree could result in performance differences. However, we did not include the phylogeny computation step in our benchmark, which focuses on SNP calling. We edited the manuscript to clarify this important point in the Results section (lines 154-155) and Methods section (lines 592-594).

4.    I leave it to the authors discretion, but I strongly feel it would be helpful to see a comparison to multiple genome alignment tools capable of producing SNP genotyping info, such as Mugsy or Mauve. While runtimes for these tools can be prohibitive for large datasets, on smaller datasets it might help to shed light on pros and cons of Maast vs previous approaches based on multiple genome alignment.

Slow speed is indeed the main reason that we did not include Mugsy and Mauve in our comparisons. Another reason is that both Mugsy and Mauve have been compared to ParSNP in the ParSNP paper [7], and they were reported to have similar accuracy to ParSNP. To verify the previous results, we ran both Mugsy and Mauve with default parameters on a small subset (n=50) of the simulated genomes used in this study. We found that Mugsy and Mauve finished building the multiple sequence alignment in ~1.5h and ~3h, respectively. Both Mugsy and Mauve showed perfect TPR and FPR in the task of recovering the simulated SNPs. Thus, while these tools are accurate, they are not computationally efficient enough to include in the large benchmarks in this study. We edited the Methods section (lines 538-540) to clarify these reasons for choosing to use ParSNP.

Minor comments

5.    It should be noted that Maast does not produce a multiple sequence alignment, and as far as I can tell, excludes insertion and deletion information.

We would like to clarify that Maast does include a multiple sequence alignment in multi-FASTA format as part of its default outputs. However, Maast does not identify insertion and deletion variants (INDELs) or structural variants. The Methods section (lines 454-456) has been edited to emphasize these points.

6.    When running on a set of 100 SARS-CoV-2 genomes, with the following command maast end_to_end --in-dir ./genomes-100/ --out-dir maast-out-100
the software crashed with the following error:

   "    cur_aln.ncols = len(cur_aln.seqs[0].seq)"
   "    IndexError: list index out of range"

Thanks for reporting this error. We received a report of a similar issue from an independent user, and we identified a minor bug related to the file path parsing. The bug is fixed now.

7.    Strongly encourage the authors to make the Maast software available on bioconda; it seems to be only available on their own conda channel

We still anticipate frequent updates at the current stage, but we are highly interested in making Maast available on bioaconda upon the publication of the paper.

8.    Maast should not default to using all available cores

We agree and have now changed the default number of cores that Maast uses to be one core.

9.    It would be very informative and helpful to see a runtime breakdown of the pipeline per step

Great idea! Here is a runtime breakdown for an example species (*Bacteroides uniformis*): ~7 mins for picking tag genomes and generating SNP panel, ~13 mins for building sck-mer database, and ~19 mins for genotyping 2,746 whole-genome assemblies or ~90 secs for 63 samples of whole-genome sequencing short reads. To address this comment, we added this information in the Methods section (lines 601-604).

10.    Axis labels and font size on figures 2-5 is quite small and difficult to read.

Thanks for bringing this issue to our attention. We remade all these figures (Figures 2, 3, 4 and 5) with the label and font sizes increased.

**References**

1. Iqbal Z, Turner I, McVean G. High-throughput microbial population genomics using the Cortex variation assembler. Bioinformatics. 2013;29:275–6.

2. Turner I, Garimella KV, Iqbal Z, McVean G. Integrating long-range connectivity information into de Bruijn graphs. Bioinformatics. 2018;34:2556–65.

3. Zhao C, Shi ZJ, Pollard KS. Pitfalls of genotyping microbial communities with rapidly growing genome collections. Cell Systems. 2023;14:160-176.e3.

4. Li W, Godzik A. Cd-hit: a fast program for clustering and comparing large sets of protein or nucleotide sequences. Bioinformatics. Oxford University Press; 2006;22:1658–9.

5. Rognes T, Flouri T, Nichols B, Quince C, Mahé F. VSEARCH: a versatile open source tool for metagenomics. Hrbek T, editor. PeerJ. 2016;4:e2584.

6. Steinegger M, Söding J. MMseqs2 enables sensitive protein sequence searching for the analysis of massive data sets. Nature Biotechnology. 2017;35:1026–8.

7. Treangen TJ, Ondov BD, Koren S, Phillippy AM. The Harvest suite for rapid core-genome alignment and visualization of thousands of intraspecific microbial genomes. Genome Biology. 2014;15:524.

**Second round of review**

**Reviewer 1**

Shi et al. have now submitted a revised version of their manuscript describing the Maast microbial SNP genotyping algorithm. In the revised version, the authors comprehensively address my comments and questions from the first round of reviews. The revised version adds helpful details on many important aspects.

Based on the revised version and the response-to-reviewers document, a few follow-up questions have come up that I would recommend be addressed prior to publication.

Major:

- The authors report that they attempted to run Cortex on a dataset comprising 45 bacterial genomes, failing, however, due to memory- and performance-related issues. The authors conclude that pangenome methods "are much slower and require a lot of RAM, and hence they do not scale well in their current implementations to the increasingly large datasets being analyzed in the metagenomics field".

I do not doubt that the authors made a bona fide attempt to run Cortex on their data; however, based on the Iqbal et al. Bioinformatics app note from 2013 (and the fact that Cortex has been successfully applied in a large number of genome analysis projects), I strongly suspect that something has gone wrong with the authors’ Cortex experiment. Specifically, Iqbal et al. demonstrate the analysis of 72 S. aureus genomes with 10 GB of RAM, and estimate that the analysis of 1000 Staph aureus genomes would take 106 GB of RAM – i.e. the 45 genomes case tried by the authors should be comfortably within the limits of the hardware the authors use for their experiments.

Given the conceptional similarities between Maast and Cortex - catalog-first-genotype-later as well as kmer-based genotyping - I would encourage the authors to make an additional attempt at getting Cortex to run on their data, potentially following the workflow described in the 2013 Bioinformatics papers. In case this proves not successful, I would recommend the authors reach out to the Iqbal lab.

- The definitions of TP and FP still seem to be not quite right - "True positives (TP) and false positives (FP) are correct and incorrect genotypes compared to the ground truth; false negatives (FN) are sites with no genotype or an incorrect genotype" - that is, as stated, incorrect genotypes count towards both TP and FP. In addition, "These values were calculated for each method on non-reference sites only as Snippy only reports them" - "non-reference sites" as defined in the ground truth, or as output by the evaluated method? Does this mean that positions which carry the reference allele are ignored in the comparative evaluation? I am not sure this is sufficient, as Maast will provide an explicit call at these positions, and - at least assumedly - the absence of a call in a region with sufficient coverage in the Snippy output could be interpreted as a "reference" call. I would therefore recommend that the authors add (this could be in the Supplement) an evaluation in which they report the PPV of all calls explicitly appearing in the output of each algorithm (reference or not), as well as sensitivity for reference and non-reference alleles; furthermore, I would recommend first including Snippy as-is, and complemented this by an evaluation in which non-calls are interpreted as reference calls.

Minor:

- The description of how Maast constructs a multiple sequence alignment of the tag reference genomes does still not seem quite correct from a formal point of view? A set of pairwise sequence alignments between a reference and additional sequences does not by itself induce a valid multiple sequence alignment between the reference and the additional sequences, so some further processing would seem to be required. In addition (this is more about an intuitive understanding of the process, not about formal correctness), the different pairwise alignments identified by MUMmer exhibit different start and end coordinates along the centroid reference genome - is the right way to think about these pairwise alignments as a "pileup" along the centroid reference genome? Perhaps a small figure may be helpful here.

- Regarding the genotyping process: The authors state that "If both alleles are detected, the counts of both alleles with be reported" - i.e. in this case the user will receive a "pseudo-diploid" call? In the context of a haploid bacterial genomes, what would be the interpretation of that? Furthermore, how are such calls evaluated in the reported benchmarking studies - independently per allele?

**Reviewer 2**

great job with the revisions, no further comments

**Authors Response**

**Point-by-point responses to the reviewers’ comments:**

Reviewer #1: Shi et al. have now submitted a revised version of their manuscript describing the Maast microbial SNP genotyping algorithm. In the revised version, the authors comprehensively address my comments and questions from the first round of reviews. The revised version adds helpful details on many important aspects.

We are happy that the revision addressed the prior round of comments.

Based on the revised version and the response-to-reviewers document, a few follow-up questions have come up that I would recommend be addressed prior to publication.

Major:

- The authors report that they attempted to run Cortex on a dataset comprising 45 bacterial genomes, failing, however, due to memory- and performance-related issues. The authors conclude that pangenome methods "are much slower and require a lot of RAM, and hence they do not scale well in their current implementations to the increasingly large datasets being analyzed in the metagenomics field".

I do not doubt that the authors made a bona fide attempt to run Cortex on their data; however, based on the Iqbal et al. Bioinformatics app note from 2013 (and the fact that Cortex has been successfully applied in a large number of genome analysis projects), I strongly suspect that something has gone wrong with the authors’ Cortex experiment. Specifically, Iqbal et al. demonstrate the analysis of 72 S. aureus genomes with 10 GB of RAM, and estimate that the analysis of 1000 Staph aureus genomes would take 106 GB of RAM – i.e. the 45 genomes case tried by the authors should be comfortably within the limits of the hardware the authors use for their experiments.

Given the conceptional similarities between Maast and Cortex - catalog-first-genotype-later as well as kmer-based genotyping - I would encourage the authors to make an additional attempt at getting Cortex to run on their data, potentially following the workflow described in the 2013 Bioinformatics papers. In case this proves not successful, I would recommend the authors reach out to the Iqbal lab.

With extraordinary help from Dr. Iqbal via dozens of emails and a zoom call, we finally were able to successfully run Cortex on the 45 strains used in our previous comparisons. We note that Maast relies on a single reference genome per species for all variant calls, whereas Cortex can use multiple genomes as a reference and call variants that are outside any individual genome indexed. Therefore, we ran Cortex with a reference index we built from five conspecific genomes per species, specifically four randomly selected genomes plus the original reference genome used in our previous comparisons. Using the resulting Cortex outputs, we mounted the requested comparisons.

The running of Cortex took >8 hours to finish (>250X longer compared to Maast), and produced a total of 373,611 variants, which is ~2.46X more than what Maast called with a single reference genome (n=151,602). Reassuringly, the genotyping results of Maast and Cortex are highly concordant as >93% of the genotypes (n=141,410) called by Maast are also called by Cortex. We summarized these results as follows: “To compare Maast to a pangenome SNP calling method, we ran Cortex [21] on the 45 *B. uniformis* strains. We used the Maast reference genome plus four additional, randomly selected genomes to generate the Cortex index. We observed that Cortex calls ~2.5 times more variants than does Maast. However, the runtime of Cortex was 250 times longer than Maast. We also found that genotyping results of Maast and Cortex are highly concordant: >93% of the SNPs called by Maast are also called by Cortex. Thus, more SNPs can be discovered with pangenome methods compared to methods like Maast that use a single reference genome. But this additional power for SNP discovery comes at a large computational cost that prohibits applications to genotyping thousands of strains.”

These results are consistent with our expectation and the original statement in the discussion section, “Pangenome methods have potential to overcome this limitation because they utilize more of the accessory genome. However, the compute and memory requirements of current implementations do not permit analysis with datasets of the scale explored in this study”. To be more accurate, we revised the original statement to “Pangenome methods [21,22] have potential to overcome this limitation, because they utilize more of the accessory genome. However, our benchmark using the Cortex algorithm demonstrated that the computing requirements of current pangenome implementations do not permit analysis with datasets of the scale explored in this study. Accelerating these algorithms is an exciting area for future research.”

- The definitions of TP and FP still seem to be not quite right - "True positives (TP) and false positives (FP) are correct and incorrect genotypes compared to the ground truth; false negatives (FN) are sites with no genotype or an incorrect genotype" - that is, as stated, incorrect genotypes count towards both TP and FP. In addition, "These values were calculated for each method on non-reference sites only as Snippy only reports them" - "non-reference sites" as defined in the ground truth, or as output by the evaluated method? Does this mean that positions which carry the reference allele are ignored in the comparative evaluation? I am not sure this is sufficient, as Maast will provide an explicit call at these positions, and - at least assumedly - the absence of a call in a region with sufficient coverage in the Snippy output could be interpreted as a "reference" call. I would therefore recommend that the authors add (this could be in the Supplement) an evaluation in which they report the PPV of all calls explicitly appearing in the output of each algorithm (reference or not), as well as sensitivity for reference and non-reference alleles; furthermore, I would recommend first including Snippy as-is, and complemented this by an evaluation in which non-calls are interpreted as reference calls.

To be more accurate, we change the sentence to “True positives (TP) are correct genotypes compared to the ground truth, and false positives (FP) are incorrect genotypes;”. No, we did not include the reference alleles in the original analyses to ensure a fair comparison with Snippy, since Snippy only reports variant calls and does not report reference calls. As suggested, we now report the PPV and sensitivity of Maast calculated using non-reference alleles for direct comparison with Snippy and using all alleles (both reference and non-reference) in a new figure (Figure S11b). We left Snippy out of the latter comparison, because it does not report reference calls. Compared to what is calculated based on only non-reference alleles, the PPV of Maast calculated based on all alleles is slightly higher and near perfect, and the sensitivity is essentially unchanged. These results suggest that the genotyping accuracy of Maast is high regardless of the allele type.

**Figure S11. (b)** Comparison of Maast genotyping accuracy with and without including reference alleles. We compared performance estimated two ways: using both reference and non-reference alleles (all) versus using only non-reference alleles (variant). Comparisons were performed based on both simulated (left) and downloaded (right) whole-genome sequencing reads. SNPs in the Maast SNP panel were called in forward reads only by running Maast with default settings. (upper) Positive predictive value (PPV) comparison, where true positive discoveries are genotype calls that match the genome. (lower) Sensitivity from simulations with the Maast SNP panel as reference. Sensitivity is the probability of detecting genotypes present in the genome.

Minor:

- The description of how Maast constructs a multiple sequence alignment of the tag reference genomes does still not seem quite correct from a formal point of view? A set of pairwise sequence alignments between a reference and additional sequences does not by itself induce a valid multiple sequence alignment between the reference and the additional sequences, so some further processing would seem to be required. In addition (this is more about an intuitive understanding of the process, not about formal correctness), the different pairwise alignments identified by MUMmer exhibit different start and end coordinates along the centroid reference genome - is the right way to think about these pairwise alignments as a "pileup" along the centroid reference genome? Perhaps a small figure may be helpful here.

Yes, the multiple sequence alignment was generated by piling up pairwise alignments along the reference genome. We now added a small figure for the visualization of this step (Figure S2a).

**Figure S2a. Scheme whereby a Multiple Sequence Alignment is built from pairwise genome alignments.** Each line represents a conspecific genome assembly. Each rectangle represents an aligned genomic region identified through pairwise genome alignments. The lightning bolt symbol indicates SNP sites.

- Regarding the genotyping process: The authors state that "If both alleles are detected, the counts of both alleles with be reported" - i.e. in this case the user will receive a "pseudo-diploid" call? In the context of a haploid bacterial genomes, what would be the interpretation of that? Furthermore, how are such calls evaluated in the reported benchmarking studies - independently per allele?

Thanks for the question. The detection of both alleles could be due to the presence of more than one strain, de novo mutations, or sequencing errors in the sample. We design the output of Maast to report the alleles as detected and delegate further processing of the alleles to downstream analysis. In this study, we performed postprocessing by assuming there was only one dominant strain per sample for a vast majority of the samples, therefore when both major and minor alleles were detected for a SNP, we proceeded with the major allele (allele frequency > 0.5) and ignored the minor allele for all analyses unless otherwise mentioned. We now note this important information in the methods section.

Reviewer #2: great job with the revisions, no further comments

Thanks!
